# Visible-Light Photocatalytic Functionalization of Isocyanides for the Synthesis of Secondary Amides and Ketene Aminals

**DOI:** 10.1021/acs.joc.0c01946

**Published:** 2020-10-19

**Authors:** Rolando Cannalire, Jussara Amato, Vincenzo Summa, Ettore Novellino, Gian Cesare Tron, Mariateresa Giustiniano

**Affiliations:** † Department of Pharmacy, University of Naples Federico II, via D. Montesano 49, 80131 Napoli, Italy; ‡ Department of Drug Science, University of Piemonte Orientale, Largo Donegani 2, 28100 Novara, Italy

## Abstract

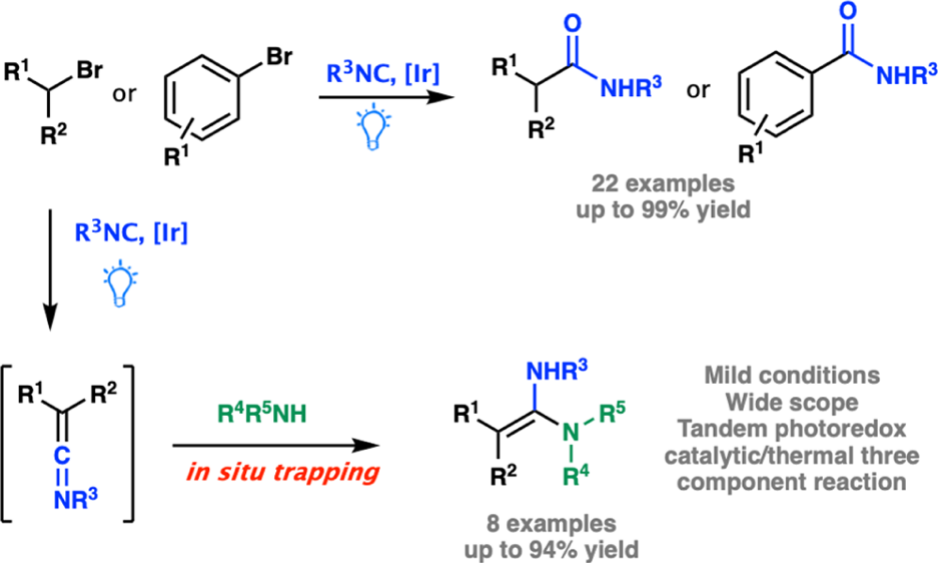

A new visible light-induced photocatalytic protocol enabling the formation of secondary amides from electron-poor organic bromides and isocyanides was developed. In addition, the in situ interception of ketenimine intermediates with nitrogen nucleophiles such as amines, hydrazines, and TMSN_3_ afforded, in a one-pot two-step procedure, valuable scaffolds such as ketene aminals, pyrazolones, and tetrazoles. Mechanistic evidence confirmed a radical pathway where isocyanides acted as radical geminal acceptors generating key imidoyl radical species.

## Introduction

**Scheme 1:**
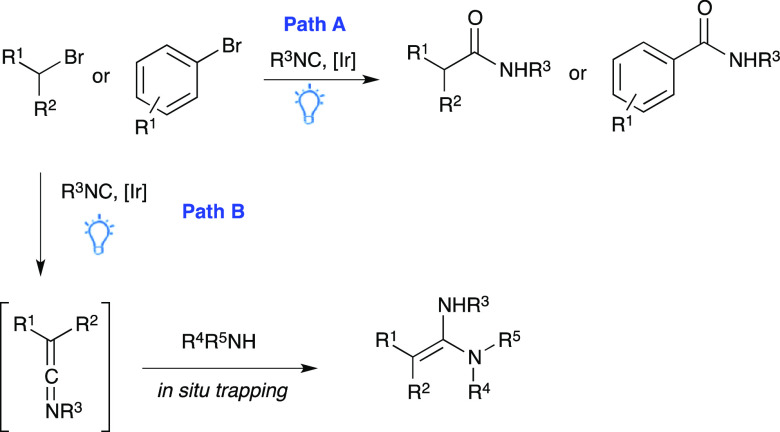
Photocatalytic Synthetic Protocol Developed for the Synthesis of Amides and Amidines

The amide bond is one of the most important linkages in biological molecules and is present in 25% of marketed drugs.^1^ Synthetic methods to achieve amide bond formation conventionally rely on coupling of carboxylic acids with amines in the presence of stoichiometric or excess condensing agents,^2^ whereas catalytic procedures exploiting boric acid and boron-based reagents as well as transition-metal-catalyzed^3^ and *N*-heterocyclic carbine-catalyzed oxidative couplings of aldehydes with amines^4^ represent less conventional approaches. On the other hand, photoredox catalysis is emerging as a powerful tool for chemists as it allows us to exploit visible-light-absorbing photocatalysts, which upon electron or energy transfer processes are able to sensitize organic molecules and trigger a photochemical reaction.^5−8^ In this context, some visible-light-mediated strategies have been reported,^9−11^ and in particular, the ability of isocyanides to act as geminal radical acceptors to yield amides was recently demonstrated by Rohe et al. describing the UV-light generation of alkyl radicals from bromoalkanes in the presence of a dimeric gold(I) photoredox catalyst.^12^ Following our interest in the study of isocyanides as radical acceptors,^13^ we envisaged that visible-light photoredox generation of C-centered radicals from simple precursors such as bromoalkanes, followed by a somophilic isocyanide insertion, could advantageously lead to amide derivatives (Scheme 1, path A). During the preparation of this article, Huang et al. reported a nickel-catalyzed aminocarbonylation of alkyl iodides with isocyanides, which, however, was limited to tertiary isocyanides and aliphatic iodines.^14^

As an added value, the protocol herein reported enabled a photoinduced multicomponent one-pot-two-step synthesis of ketene aminals. Indeed, the treatment of strong α-acidic starting alkyl bromides (such as diethyl bromomalonate) under standard reaction conditions afforded valuable ketenimines intermediates, which were intercepted in situ with both aliphatic and aromatic amines (Scheme 1, path B).

**Scheme 2:**
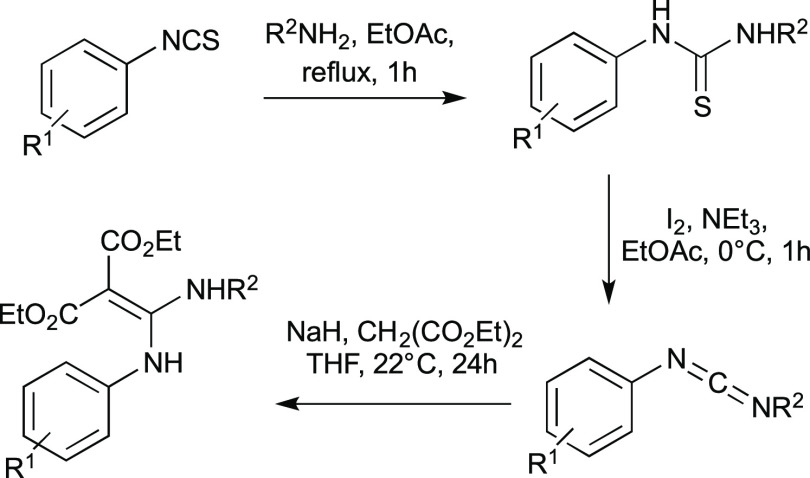
Literature-Reported Synthesis of Disubstituted Ketene Aminals

Ketene aminals, structurally related to amidines, behave as bioisosteres of thiourea, amidine, and guanidine moieties, with improved pharmacokinetic properties, as shown in histamine H2-receptor antagonists (e.g., ranitidine)^15^ and FXa inhibitors.^16^ Their synthesis is usually accomplished in three reaction steps starting from an isothiocyanate^17^ (Scheme 2) and is associated with several drawbacks such as the use of toxic and hazardous reagents (e.g., molecular iodine and sodium hydride) and the need for cooling (0 °C) or refluxing the reaction mixture. Even more importantly, starting from an isothiocyanate limited this synthetic approach to disubstituted ketene aminals with both nitrogen atoms bearing one substituent, whereas the use of formamidinium salts can lead to tetrasubstituted symmetrical ketene aminals.^18^ Accordingly, the obtainment of unsymmetrical trisubstituted products is still considered a challenging task.

## Results and Discussion

We preliminarily explored the feasibility of amides’ formation by reacting ethyl bromoacetate **1** (1.5 equiv) and *tert*-butyl isocyanide **2** as test substrates, in the presence of *fac*-Ir(ppy)_3_ (1%) as the photocatalyst, Na_2_CO_3_ as the base (1.5 equiv), in acetonitrile, and in the presence of H_2_O (20 equiv) (Table 1—entry 1).

**Table 1:**
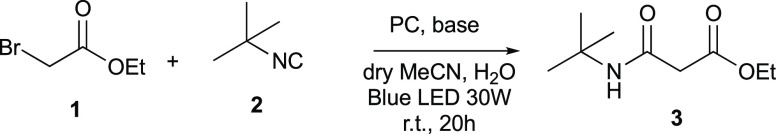
Optimization of Reaction Conditions^a^

entry	equiv of **1**^a^	PC	DABCO (equiv)	base (equiv)	H_2_O (equiv)	yield (%)
1	1.5	*fac*-Ir(ppy)_3_ (1%)^b^		Na_2_CO_3_ (1.5)	20	54
2	1.5	*fac*-Ir(ppy)_3_ (1%)^b^	1	Na_2_CO_3_ (1.5)	20	traces
3	3	*fac*-Ir(ppy)_3_ (1%)^b^	1	Na_2_CO_3_ (3)	20	97
4	1.5	*fac*-Ir(ppy)_3_ (1%)^b^	0.5	Na_2_CO_3_ (1.5)	20	96
5	1.5	Ru(bpy)_3_(PF_6_)_2_ (1%)^b^	0.5	Na_2_CO_3_ (1.5)	20	traces^c^
6	1.5	Ru(bpy)_3_·6H_2_O^b^ (1%)	0.5	Na_2_CO_3_ (1.5)	20	traces^c^
7	1.5	Ru(bpy)_3_PF_6_ (1%)^b^		Na_2_CO_3_ (1.5)	20	traces^c^
8	1.5	eosin Y (5%)^d^	0.5	Na_2_CO_3_ (1.5)	20	ND
9	1.5	eosin Y (5%)^d^		Na_2_CO_3_ (1.5)	20	ND
10	1.5	rose bengal (1%)^e^^,^^f^				ND
11	1	*fac*-Ir(ppy)_3_ (1%)^b^	0.33	Na_2_CO_3_ (1)	20	55^c^
12	1	*fac*-Ir(ppy)_3_ (1%)^b^	0.33	Na_2_CO_3_ (1)	MeCN/H_2_O 1:1	73^c^
13	1.5	*fac*-Ir(ppy)_3_ (1%)^b^	0.5	Na_2_CO_3_ (1.5)	MeCN/H_2_O 1:1	84^c^
14	1.5	*fac*-Ir(ppy)_3_ (1%)^b^	0.5	Na_2_CO_3_ (1.5)	MeCN/H_2_O 1:3	78^c^
15	1.5	*fac*-Ir(ppy)_3_ (1%)^b^	0.5	Na_2_CO_3_ (1.5)	100%	62
16	1.5	*fac*-Ir(ppy)_3_ (1%)^b^	0.5	Na_2_CO_3_ (1.5)		ND
17	1.5	*fac*-Ir(ppy)_3_ (1%)^f^	0.5	Na_2_CO_3_ (1.5)	20	98%
18	1.5	*fac*-Ir(ppy)_3_ (1%)^g^	0.5	Na_2_CO_3_ (1.5)	20	ND
19	1.5	*fac*-Ir(ppy)_3_ (1%)^h^	0.5	Na_2_CO_3_ (1.5)	20	ND
20	1.5	*fac*-Ir(ppy)_3_ (1%)	0.5		20	25%
21	1.5	*fac*-Ir(ppy)_3_ (1%)	0.5	Na_2_HPO_4_ (1.5)	20	96%
22	1.5	*fac*-Ir(ppy)_3_ (1%)	0.5	Na_2_HPO_4_ (1.5)	20	46%
23	1.5		0.5	Na_2_CO_3_ (1.5)	20	ND

a Compound **2** 0.25 mmol in MeCN 0.1 M.

b Degassing by freeze–pump–thaw.

c Yield by ^1^H NMR.

d Green LED 16 W.

e EtOAc as the solvent.

f No degassing under an Ar atmosphere.

g Open flask.

h Under a O_2_ atmosphere.

After 20 h under blue light-emitting diodes (LEDs) irradiation (30 W), at room temperature, we were able to isolate the desired product **7a** in a fair 54% yield. In order to improve the reaction yield, the equivalents of the starting alkyl halide, the catalyst, the equivalents and the nature of the base, and different amounts of water were screened. The addition of a sacrificial electron donor, such as diazabicyclo[2.2.2]octane (DABCO) (1 equiv), led to a dramatic drop in the yield (Table 1—entry 2), while the use of the same amount of DABCO (1 equiv) along with the 2-fold increase of both bromide **1** and Na_2_CO_3_ led to an excellent yield of 97% (Table 1—entry 3). Interestingly, a comparable high yield (96%) was obtained halving both DABCO, **1**, and Na_2_CO_3_ (Table 1—entry 4). Ruthenium-based catalysts proved to be unable to promote the reaction both in the presence and in the absence of DABCO (Table 1—entries 5–7). Similarly, when the reaction was performed with photoactive organic dyes, such as Eosin Y and Rose Bengal, only starting materials were detectable (Table 1—entries 8–10). Attempted further optimization of reaction conditions (Table 1—entries 11–16) led to decreased yields. Very interestingly, the quantitative yield (Table 1—entry 4) was retained also without rigorous degassing under an inert atmosphere (Ar balloon) (entry 17). The presence of the inorganic base Na_2_CO_3_ was also essential (Table 1—entry 20), while it can be efficiently replaced by Na_2_HPO_4_ (Table 1—entry 21) but not by NaH_2_PO_4_ (Table 1—entry 22), indicating that at least a base with p*K*_a_ > 7 is required to retain high yields. Finally, the photoredox nature of the transformation was undoubtedly demonstrated (Table 1—entry 23).

**Figure 1:**
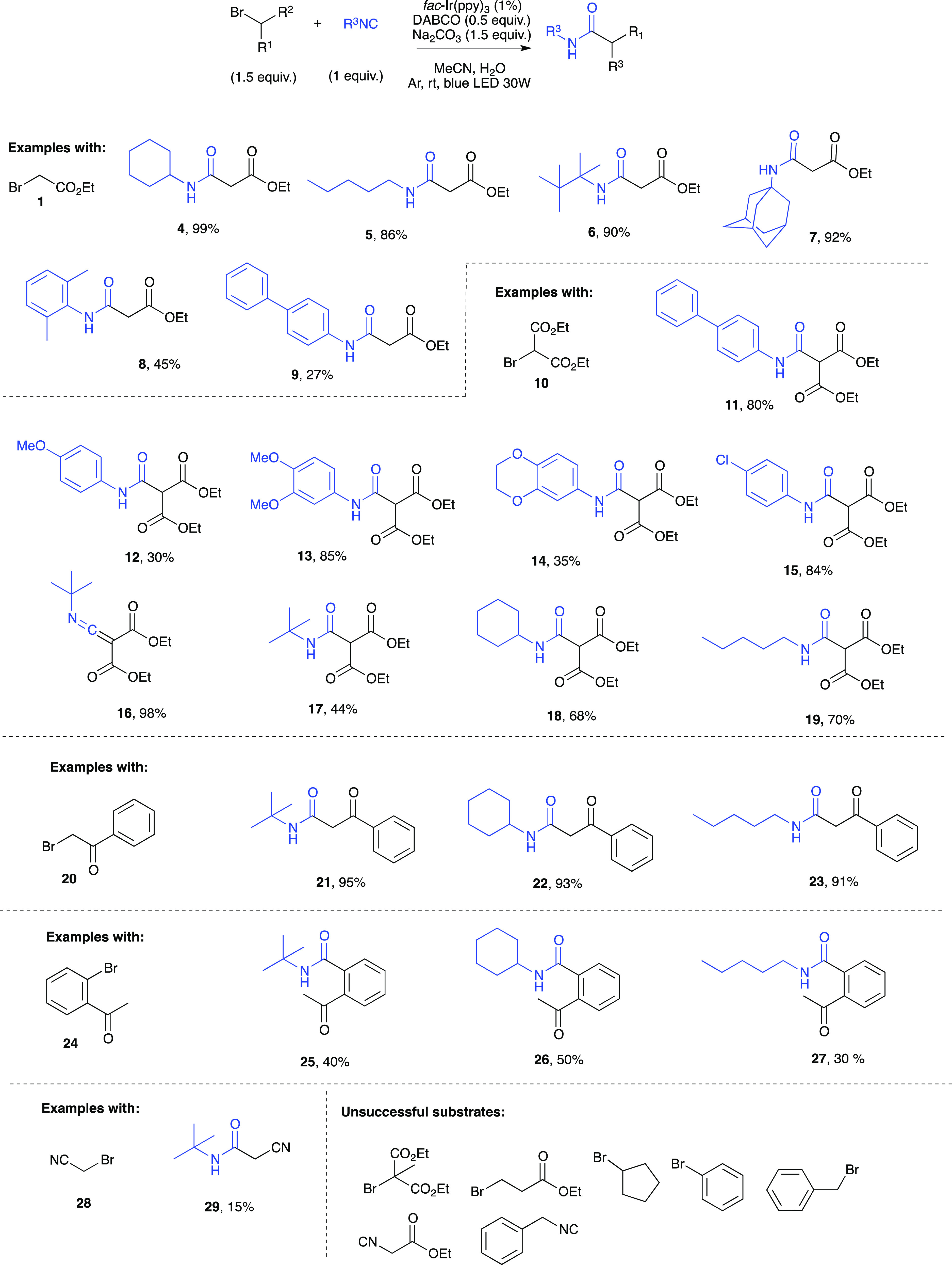
Reaction scope (standard reaction conditions as in Table 1, entry 17 performed on a 0.25 mmol scale).

By adopting the optimized reaction conditions (Table 1—entry 17), we then investigated the generality of this transformation by evaluating a variety of isocyanides and both alkyl and aryl bromides as alkyl and aryl radical precursors, respectively. As depicted in Figure 1, keeping ethyl bromoacetate **1** constant, excellent yields (**4–7**, 86–99%) were obtained by reacting different aliphatic primary, secondary, and tertiary isocyanides, regardless the presence of either cyclic or linear bulky α-branched substituents. Yet, aromatic isocyanides (ArNC) were less efficient substrates affording aryl amides **8** and **9** in moderate yields. Remarkably, by changing the alkyl bromide counterpart (e.g., diethyl 2-bromomalonate **10**), both electron-rich and electron-poor aryl isocyanides afforded compounds **11–15** in moderate to high yields (30–85%). On the other hand, reaction of aliphatic isocyanides under standard conditions led to the quantitative formation of the ketenimine **16**. We reasoned that its formation could depend on the strength of the inorganic base. Thus, different weaker bases such as NaHCO_3_, Na_2_HPO_4_, and NaH_2_PO_4_ were screened, with NaH_2_PO_4_ (p*K*_a_ = 2.1) able to afford amide **17**, although in a moderate yield of 44%. Still, this reoptimized reaction conditions proved to be general as compounds **18** and **19** were obtained in 68 and 70% yields, respectively.

Further experiments indicated that the outcome of the reaction was strongly influenced by the electronic nature of the bromide reagent. Actually, the presence of an electron-withdrawing group demonstrated to be essential, as 2-bromoacetophenone **20** gave products **21–23** in excellent yields, while more electron-rich alkyl bromides failed to give the desired amides. Still, aryl bromides with strong electron-withdrawing substituents in the ortho position such as 2′-bromoacetophenone **24** afforded the corresponding amides **25–27**, while deactivated bromobenzene did not react at all. Finally, the switch from the ester moiety of alkyl bromide **1** to a cyano-functional group in **28**, albeit still exerting an electron-withdrawing effect, showed to be detrimental (**29**).

**Scheme 3:**
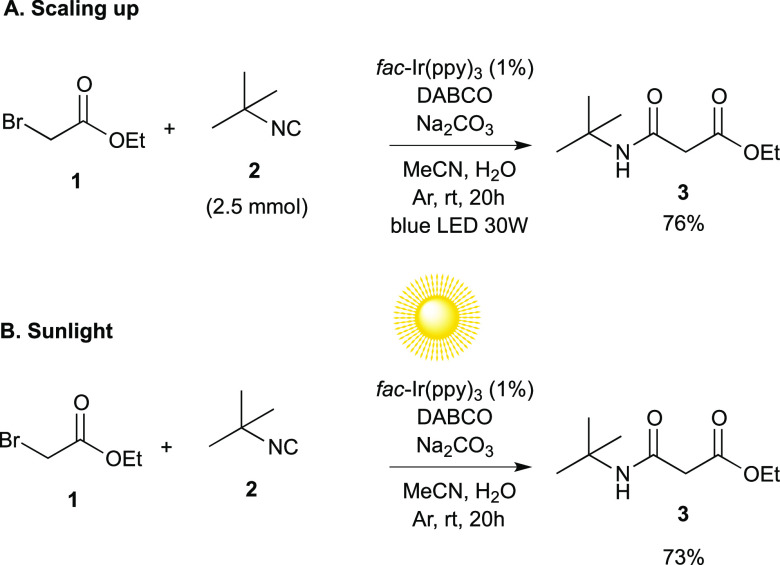
(A) Scale-Up Reaction; (B) Natural Sunlight-Induced Reaction

To further demonstrate the preparative utility of this methodology, a scale-up synthesis of **3** was successfully carried out (76% yield) by using 2.5 mmol of *t*BuNC **2** under the standard reaction conditions (Scheme 3A). Noteworthy, performing the reaction under sunlight irradiation provided the target compound **3** in a good 73% yield (Scheme 3B).

**Figure 2:**
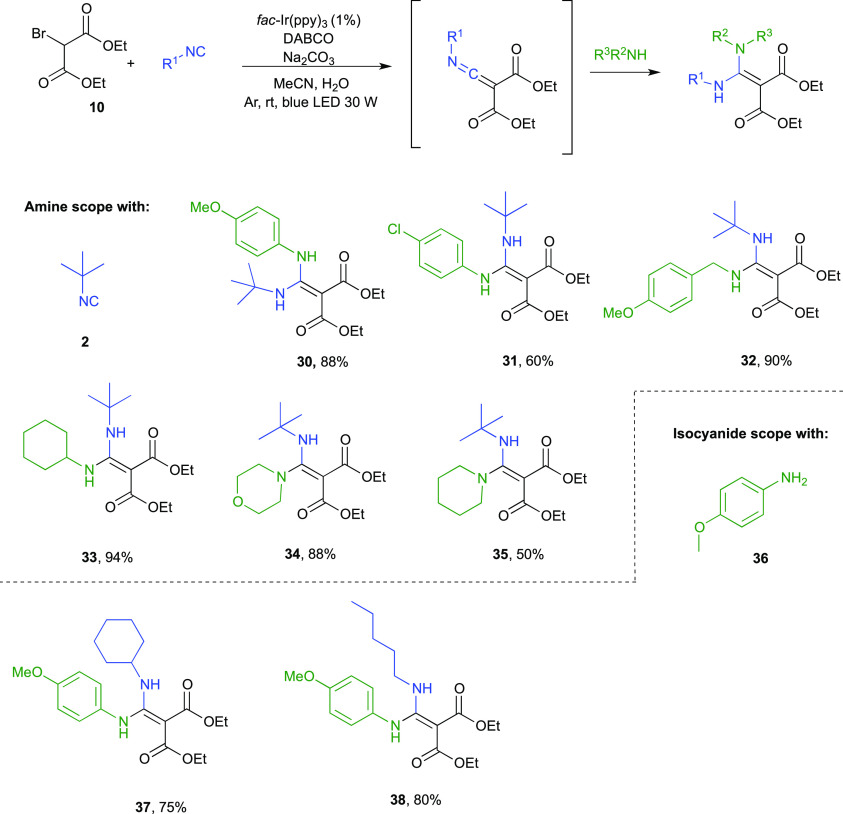
One-pot two-steps visible light photocatalytic synthesis of amidines (yields are calculated over the two steps).

As depicted in Figure 1, diethyl bromomalonate **10** and *t*BuNC **2** quantitatively afforded the ketenimine **16** under our reaction conditions in presence of Na_2_CO_3_. As a matter of fact, there are few methods reported in the literature to produce such compounds, and Zhu and co-workers^19^ recently published the thermal palladium-catalyzed reaction of α-haloketones with isocyanides using toluene as the solvent. We herein provide a mild photocatalytic protocol in a green solvent system and at room temperature. Moreover, the relatively stable ketenimines can be intercepted in situ by different nucleophiles, thereby acting as a chemical platform for divergent synthesis. Thus, we decided to explore such reactivity in a multicomponent one-pot two-steps procedure. After the starting *t*BuNC **2** and diethyl bromomalonate **10** were converted into ketenimine **16**, an amine was added in situ, while kept under stirring without further irradiation overnight, to afford the corresponding ketene aminal.^20^ As shown in Figure 2, both aliphatic and aromatic amines proved to be competent substrates (ketene aminals **30–35**). Besides *t*BuNC, secondary and primary isocyanides such as cyclohexyl- and *n*-pentylisocyanide also proved to be suitable substrates as shown with products **37** and **38** (75 and 80% yield, respectively; Figure 2).

**Scheme 4:**
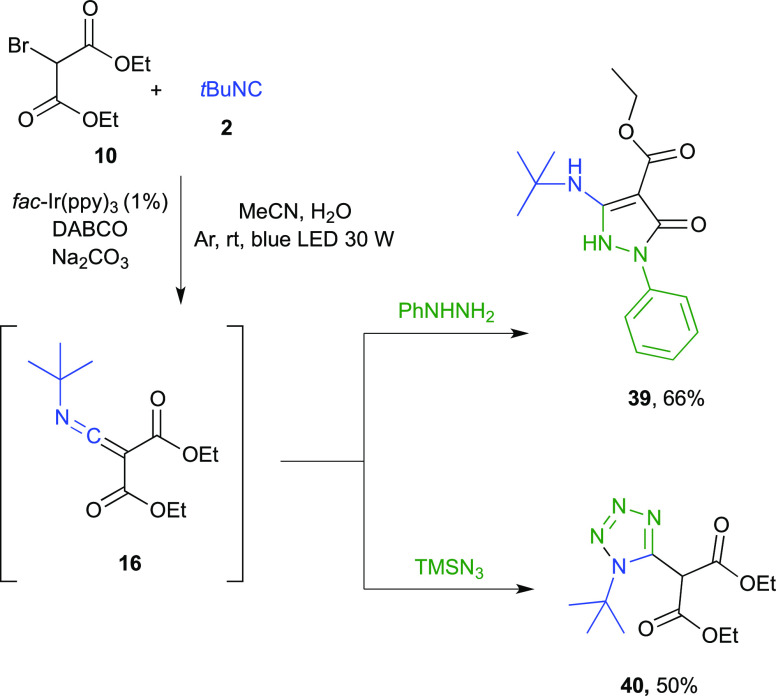
Photocatalytic Generation of Ketenimine **16** and Its Use as a Chemical Platform

Furthermore, the in situ-generated ketenimine could be considered as a linchpin to gain access to diversely functionalized heterocycles such as pyrazolones and tetrazoles (Scheme 4). Actually, the addition of phenylhydrazine in situ led to product **39** in a 66% overall yield through a domino nucleophilic addition/intramolecular transamidation, while the use of TMSN_3_ afforded, via a [3 + 2] cycloaddition, the tetrazole derivative **40** in good yield (50% overall yield).

**Figure 3:**
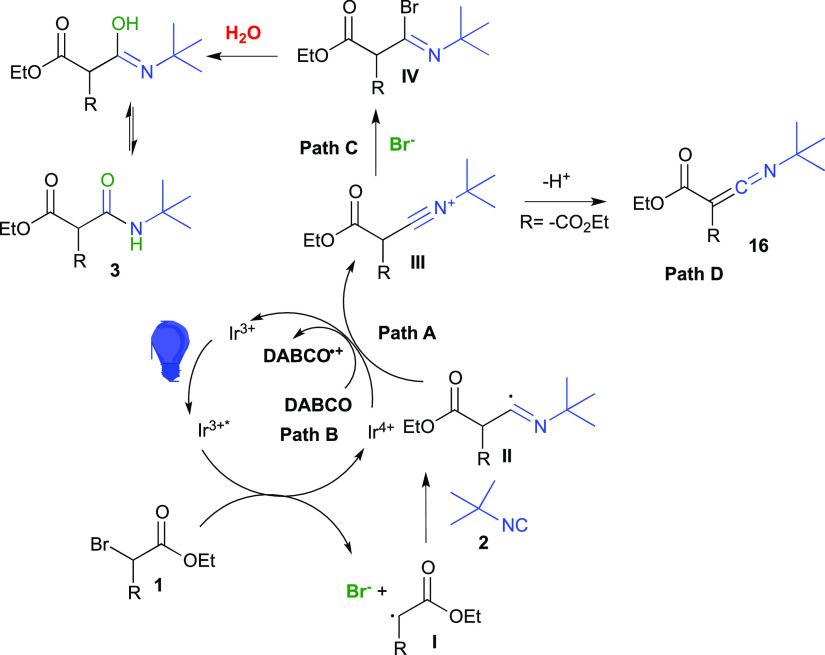
Mechanistic hypothesis.

A plausible mechanism for the reaction entails an oxidative quenching cycle (Figure 3).^6,20^ Under visible-light irradiation, the photocatalyst *fac*-Ir(ppy)_3_ undergoes a metal-to-ligand charge transfer populating the excited state Ir^III^* (*E*(Ir^IV^/Ir^III^* = −1.73 V vs SCE)),^21^ which is quenched by bromoalkane **1** (*E*^RED^(**1**/**1**^**•–**^ = −1.08 V vs SCE)),^22,23^ generating Ir^IV^ and the corresponding alkyl radical **I**^**•**^ after loss of a bromide anion. Subsequently, **I**^**•**^ is able to give a somophilic insertion into the isocyanide **2** to form the imidoyl radical **II**^**•**^. The latter can be oxidized to nitrilium ion **III** by the Ir^IV^ catalyst generated in the first step (*E*(Ir^IV^/Ir^III^ = +0.77 V vs SCE))^21^ (path A, Figure 3). Alternatively, DABCO could act as a sacrificial electron donor to regenerate the Ir^III^ photocatalyst (path B). The nitrilium ion **III** is then intercepted by the bromide anion (path C) to form imidoyl bromide **IV** that is eventually hydrolyzed to amide **3**. Alternatively, when diethyl bromomalonate is used as the starting alkyl bromide, the presence of a stronger acidic α-hydrogen led to the base-mediated formation of ketenimine **16** (path D).

**Figure 4:**
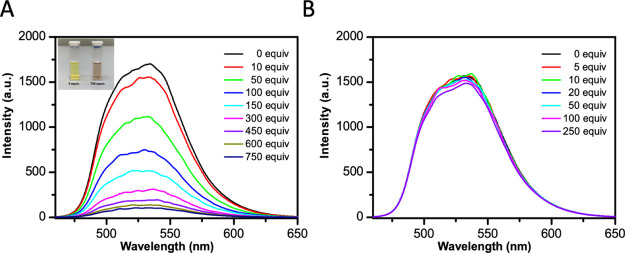
Fluorescence emission spectra of a *fac*-Ir(ppy)_3_ solution (200 μM) in the absence and presence of stepwise addition of (A) diethyl bromomalonate (**10**) and (B) DABCO at 20 °C.

This mechanistic hypothesis was further supported by Stern–Volmer quenching experiments (Figure 4). Accordingly, diethylbromomalonate **10** was able to quench a 4:1 MeCN/H_2_O solution of *fac*-Ir(ppy)_3_ (Figure 4A), whereas DABCO showed to be inefficient (Figure 4B), thus indicating a poor probability of a reductive quenching cycle.

**Scheme 5:**
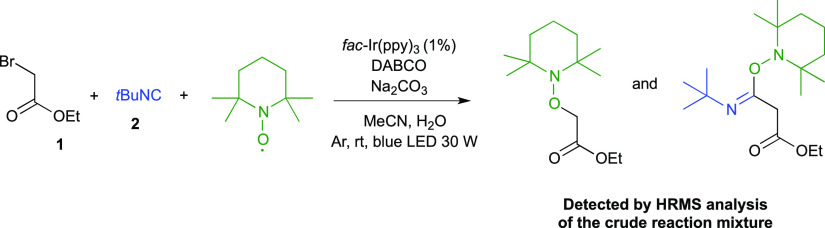
Control Experiment: Reaction Performed in the Presence of TEMPO

In order to provide further experimental evidences about the proposed mechanism, we carried out the reaction in the presence of (2,2,6,6-tetramethylpiperidin-1-yl)oxyl (TEMPO), capable of behaving as a radical trap; the resulting crude reaction mixture was analyzed by high-resolution mass spectrometry (HRMS), and two key radical intermediate–TEMPO adducts (i.e., with alkyl radical **I**^**•**^ and with imidoyl radical **II**^**•**^) were detected (Scheme 5 and Figures S1 and S2, Supporting Information).

## Conclusions

In conclusion, by combining easily available starting materials, such as organic halides and isocyanides, we developed a new visible light induced photocatalytic protocol enabling the formation of secondary amides under mild conditions.

A range of electron-poor aliphatic and aromatic halides smoothly underwent amidation with primary, secondary, and tertiary aliphatic isocyanides as well as with aromatic ones, thus demonstrating a wide scope and a good functional group tolerance. Additionally, the successful in situ interception of ketenimine intermediates with nitrogen nucleophiles such as aromatic and both primary and secondary aliphatic amines provided a new protocol to access challenging unsymmetrical trisubstituted ketene aminals. Still, ketenimine intermediates could be used as a chemical platform to synthesize, in a 3-component-2-steps procedure, valuable drug-like structural frameworks such as pyrazolones and tetrazoles. In sum, these applications further validated the robustness of the developed photoredox catalytic system, which did not interfere with nor prevented the domino transformations attempted, thus highlighting the potential for future progresses in the identification of new tandem photocatalytic/thermal processes, a valuable but underexplored class of multicomponent reactions.

## Experimental Section

### General Methods

Commercially available reagents and solvents were used without further purification. When necessary, the reactions were performed in oven-dried glassware under a positive pressure of dry nitrogen. Photochemical reactions were carried out using a PhotoRedOx Box (EvoluChem) with a 30 W blue LED (EvoluChem, model: HCK1012-01-008, wavelength 450 nm, LED: CREE XPE. A holder suitable for 4 mL scintillation vials (45 × 14.7 mm) has been fitted with the Schlenk flask: this allows a fixed sample placement distance from the light source). All NMR spectra were obtained with a Bruker Avance NEO 400 MHz instrument. Experiments for structure elucidation were performed in CDCl_3_ at 25 °C with a RT-DR-BF/1H-5mm-OZ SmartProbe. High-resolution electrospray ionization (ESI)-MS spectra were performed on a Thermo LTQ Orbitrap XL mass spectrometer. The spectra were recorded by infusion into the ESI source using MeOH as the solvent. Chemical shifts (δ) are reported in part per million (ppm) relative to the residual solvent peak. Column chromatography was performed on silica gel (70–230 mesh ASTM) using the reported eluents. Thin layer chromatography was carried out on 5 × 20 cm plates with a layer thickness of 0.25 mm (silica gel 60 F254) to monitor the reaction by using UV and/or KMnO_4_ as the revelation methods.

### General Procedure for the Preparation of Compounds **3–9**, **11–16**, **21–23**, **25–27**, and **29** (Method A)

To a 4 mL color-less screw-cap glass vial equipped with a magnetic stir bar were added the isocyanide (0.25 mmol), Na_2_CO_3_ (0.375 mmol, 1.5 equiv), *fac*-Ir(ppy)_3_ (0.0025 mmol, 1% mol), the organobromide (0.375 mmol, 1.5 equiv), and DABCO (0.125 mmol, 0.5 equiv). Then, 2.5 mL MeCN (0.1 M) and H_2_O 95 μL (20 equiv) were added into the reaction vial via a syringe. The resulting mixture was purged with nitrogen and then stirred under 30 W blue LED irradiation at room temperature for 20 h. Then, the reaction mixtures were poured into water, extracted with EtOAc (3 times), the collected organic layers were washed with 1 N HCl (1 time) and brine (1 time), dried over dry Na_2_SO_4_, and evaporated under vacuum. The reaction crude was purified by chromatography on silica-gel.

### General Procedure for the Preparation of Compounds **17–19** (Method B)

To a 4 mL color-less screw-cap glass vial equipped with a magnetic stir bar were added the isocyanide (0.25 mmol), NaH_2_PO_4_ (0.375 mmol, 1.5 equiv), *fac*-Ir(ppy)_3_ (0.0025 mmol, 1% mol), the organobromide (0.375 mmol, 1.5 equiv), and DABCO (0.125 mmol, 0.5 equiv). Then, 2.5 mL MeCN (0.1 M) and H_2_O 95 μL (20 equiv) were added into the reaction vial via a syringe. The resulting mixture was purged with nitrogen and then stirred under 30 W blue LED irradiation at room temperature for 20 h. Then, the reaction mixtures were poured into water, extracted with EtOAc (3 times), the collected organic layers were washed with 1 N HCl (1 time) and brine (1 time), dried over dry Na_2_SO_4_, and evaporated under vacuum. The reaction crude was purified by chromatography on silica-gel.

#### Ethyl 3-(*tert*-Butylamino)-3-oxopropanoate (**3**)

The title compound was prepared following the general procedure method A. The crude material was purified by column chromatography (*n*-hexane/ethyl acetate 1:5%) to give the product as pale-yellow oil (45 mg, 96% yield; 355.8 mg, 76% for the reaction performed on 2.5 mmol of *t*BuNC **2**). ^1^H NMR (400 MHz, CDCl_3_): δ 6.89 (br s, 1H, −N*H*), 4.19 (q, *J* = 7.1 Hz, 2H), 3.21 (s, 2H), 1.35 (s, 9H), 1.27 (t, *J* = 7.2 Hz, 3H); ^13^C{^1^H} NMR (100 MHz, CDCl_3_): δ 170.0, 164.0, 61.4, 51.3, 42.2, 28.6, 14.0; HRMS (ESI) *m*/*z*: calcd [M + H]^+^ for C_9_H_18_NO_3_^+^, 188.1281; found [M + H]^+^, 188.1273.

#### Ethyl 3-(Cyclohexylamino)-3-oxopropanoate (**4**)

The title compound was prepared following the general procedure method A. The crude material was purified by column chromatography (*n*-hexane/ethyl acetate 1:10%) to give the product as a white solid (52.8 mg, 99% yield); ^1^H NMR (400 MHz, CDCl_3_): δ 7.01 (br s, 1H, −N*H*), 4.20 (q, *J* = 7.1 Hz, 2H), 3.81 (ddd, *J* = 14.4, 10.3, 4.2 Hz, 1H), 3.28 (s, 2H), 1.96–1.84 (m, 2H), 1.75–1.65 (m, 2H), 1.64–1.55 (m, 2H), 1.44–1.13 (m, 7H); ^13^C{^1^H} NMR (100 MHz, CDCl_3_): δ 169.8, 163.9, 61.5, 48.1, 41.2, 32.8, 25.5, 24.6, 14.0; HRMS (ESI) *m*/*z*: calcd [M + H]^+^ for C_11_H_20_NO_3_^+^, 214.1438; found [M + H]^+^, 214.1430.

#### Ethyl 3-Oxo-3-(pentylamino)propanoate (**5**)

The title compound was prepared following the general procedure method A. The crude material was purified by column chromatography (*n*-hexane/ethyl acetate 1:10%) to give the product as colorless oil (43.3 mg, 86% yield). ^1^H NMR (400 MHz, CDCl_3_): δ 7.12 (br s, 1H, −N*H*), 4.20 (q, *J* = 7.1 Hz, 2H), 3.33–3.23 (m, 4H), 1.63–1.48 (m, 2H), 1.38–1.21 (m, 7H), 0.90 (t, *J* = 6.9 Hz, 3H); ^13^C NMR (100 MHz, CDCl_3_): δ ^13^C{^1^H} NMR (101 MHz, CDCl_3_): δ 169.9, 164.8, 61.5, 41.0, 39.6, 29.0 (2C), 22.3, 14.0 (2C); HRMS (ESI) *m*/*z*: calcd [M + H]^+^ for C_10_H_20_NO_3_^+^, 202.1437; found [M + H]^+^, 202.1423.

#### Ethyl 3-Oxo-3-((2,4,4-trimethylpentan-2-yl)amino)propanoate (**6**)

The title compound was prepared following the general procedure method A. The crude material was purified by column chromatography (*n*-hexane/ethyl acetate 1:4%) to give the product as a white crystal solid (54.8 mg, 90% yield): mp 53–55 °C; ^1^H NMR (400 MHz, CDCl_3_): δ 6.92 (br s, 1H, −N*H*), 4.19 (q, *J* = 7.1 Hz, 2H), 3.21 (s, 2H), 1.75 (s, 2H), 1.42 (s, 6H), 1.28 (7, *J* = 6.9 Hz, 3H), 1.00 (s, 9H); ^13^C{^1^H} NMR (100 MHz, CDCl_3_): δ 168.5, 162.0, 59.9, 53.7, 49.9, 40.9, 30.1, 29.9, 27.6, 12.5; HRMS (ESI) *m*/*z*: calcd [M + H]^+^ for C_13_H_26_NO_3_^+^, 244.1907; found [M + H]^+^, 244.1904.

#### Ethyl 3-(((3*s*,5*s*,7*s*)-Adamantan-1-yl)amino)-3-oxopropanoate (**7**)

The title compound was prepared following the general procedure method A. The crude material was purified by column chromatography (*n*-hexane/ethyl acetate 1:5%) to give the product as a white crystal solid (61 mg, 92% yield). ^1^H NMR (400 MHz, CDCl_3_): δ 6.71 (br s, 1H, −N*H*), 4.19 (q, *J* = 7.1 Hz, 2H), 3.21 (s, 2H), 2.13–1.98 (m, 9H), 1.68 (s, 6H), 1.28 (t, *J* = 7.0, Hz, 3H); ^13^C{^1^H} NMR (101 MHz, CDCl_3_): δ 168.7, 162.4, 60.1, 50.8, 41.1, 40.2, 35.1, 28.1, 12.8; HRMS (ESI) *m*/*z*: calcd [M + H]^+^ for C_15_H_24_NO_3_^+^, 266.1750; found [M + H]^+^, 266.1731.

#### Ethyl 3-((2,6-Dimethylphenyl)amino)-3-oxopropanoate (**8**)

The title compound was prepared following the general procedure method A. The crude material was purified by column chromatography (*n*-hexane/ethyl acetate 1:5%) to give the product as a pale yellow oil (26.5 mg, 45% yield). ^1^H NMR (400 MHz, CDCl_3_): δ 9.07 (br s, 1H, −N*H*), 7.20–7.16 (m, 2H), 6.79–6.75 (m, 1H), 4.26 (q, *J* = 7.1 Hz, 2H), 3.45 (s, 2H), 2.30 (s, 6H), 1.32 (t, *J* = 7.0 Hz, 3H); ^13^C{^1^H} NMR (100 MHz, CDCl_3_): δ 170.1, 162.7, 138.7, 137.3, 126.3, 117.9, 61.9, 41.5, 29.7, 21.4, 14.1; HRMS (ESI) *m*/*z*: calcd [M + H]^+^ for C_13_H_18_NO_3_^+^, 236.1281; found [M + H]^+^, 236.1262.

#### Ethyl 3-([1,1′-Biphenyl]-4-ylamino)-3-oxopropanoate (**9**)

The title compound was prepared following the general procedure method A. The crude material was purified by column chromatography (*n*-hexane/ethyl acetate 1:8%) to give the product as a yellow solid (19.1 mg, 27% yield): mp 108–110 °C; ^1^H NMR (400 MHz, CDCl_3_): δ 9.31 (br s, 1H, −N*H*), 7.69–7.63 (m, 2H), 7.61–7.55 (m, 4H), 7.47–7.41 (m, 2H), 7.38–7.33 (m, 1H), 4.28 (q, *J* = 7.2 Hz, 2H), 3.50 (s, 2H), 1.34 (t, *J* = 7.2 Hz, 3H); ^13^C{^1^H} NMR (100 MHz, CDCl_3_): δ 169.0, 161.8, 139.4, 136.4, 135.7, 127.7, 126.5, 126.1, 125.8, 119.3, 60.9, 40.3, 13.0; HRMS (ESI) *m*/*z*: calcd [M + H]^+^ for C_17_H_18_NO_3_^+^, 284.1281; found [M + H]^+^, 284.1264.

#### Diethyl 2-([1,1′-Biphenyl]-4-ylcarbamoyl)malonate (**11**)

The title compound was prepared following the general procedure method A. The crude material was purified by column chromatography (*n*-hexane/ethyl acetate 1:15%) to give the product as pale-yellow solid (71.1 mg, 80% yield): mp 113–116 °C; ^1^H NMR (400 MHz, CDCl_3_): δ 9.38 (br s, 1H, −N*H*), 7.68–7.62 (m, 2H), 7.60–7.55 (m, 4H), 7.46–7.41 (m, 2H), 7.36–7.31 (m, 1H), 4.47 (s, 1H), 4.32 (q, *J* = 7.0 Hz, 4H), 1.34 (t, *J* = 7.1 Hz, 6H); ^13^C{^1^H} NMR (100 MHz, CDCl_3_): δ 165.8, 159.9, 140.4, 137.8, 136.5, 128.8, 127.6, 127.2, 126.9, 120.5, 63.0, 59.6, 13.9; HRMS (ESI) *m*/*z*: calcd [M + H]^+^ for C_20_H_22_NO_5_^+^, 356.1492; found [M + H]^+^, 356.1477.

#### Diethyl 2-((4-Methoxyphenyl)carbamoyl)malonate (**12**)

The title compound was prepared following the general procedure method A. The crude material was purified by column chromatography (*n*-hexane/ethyl acetate 1:15%) to give the product as a white solid (23.2 mg, 30% yield); ^1^H NMR (400 MHz, CDCl_3_): δ 9.17 (br s, 1H, −N*H*), 7.49–7.43 (m, 2H), 6.90–6.83 (m, 2H), 4.43 (s, 1H), 4.30 (q, *J* = 8.6 Hz, 4H), 3.80 (s, 3H), 1.34 (t, *J* = 8.6 Hz, 6H); ^13^C{^1^H} NMR (100 MHz, CDCl_3_): δ 165.9, 159.7, 156.8, 130.3, 121.9, 114.2, 62.9, 59.5, 55.5, 13.9; HRMS (ESI) *m*/*z*: calcd [M + H]^+^ for C_15_H_20_NO_6_^+^, 310.1285; found [M + H]^+^, 310.1283.

#### Diethyl 2-((3,4-Dimethoxyphenyl)carbamoyl)malonate (**13**)

The title compound was prepared following the general procedure method A. The crude material was purified by column chromatography (*n*-hexane/ethyl acetate 1:15%) to give the product as white solid (72.1 mg, 85% yield): mp 120–122 °C; ^1^H NMR (400 MHz, CDCl_3_): δ 9.23 (br s, 1H, −N*H*), 7.32 (d, *J* = 2.4 Hz, 1H), 6.99 (dd, *J* = 8.6, 2.4 Hz, 1H), 6.82 (d, *J* = 8.6 Hz, 1H), 4.43 (s, 1H), 4.31 (q, *J* = 7.1, 4H), 3.88 (d, *J* = 8.2 Hz, 6H), 1.33 (t, *J* = 7.1 Hz, 6H); ^13^C{^1^H} NMR (100 MHz, CDCl_3_): δ 165.9, 159.7, 149.0, 146.2, 130.8, 112.1, 111.2, 104.9, 62.9, 59.5, 56.0, 56.0, 13.9; HRMS (ESI) *m*/*z*: calcd [M + H]^+^ for C_16_H_22_NO_7_^+^, 340.1390; found [M + H]^+^, 340.1371.

#### Diethyl 2-((2,3-Dihydrobenzo[*b*][1,4]dioxin-6-yl)carbamoyl)malonate (**14**)

The title compound was prepared following the general procedure method A. The crude material was purified by column chromatography (*n*-hexane/ethyl acetate 1:15%) to give the product as a white solid (29.5 mg, 35% yield): mp 124–125 °C; ^1^H NMR (400 MHz, CDCl_3_): δ 9.12 (br s, 1H, −N*H*), 7.21 (d, *J* = 2.4 Hz, 1H), 6.94 (dd, *J* = 8.7, 2.4 Hz, 1H), 6.81 (d, *J* = 8.7 Hz, 1H), 4.41 (s, 1H), 4.35–4.21 (m, 8H), 1.32 (t, *J* = 7.1 Hz, 6H); ^13^C{^1^H} NMR (100 MHz, CDCl_3_): δ 165.8, 159.6, 143.5, 140.8, 130.9, 117.2, 113.8, 110.0, 64.4, 64.3, 62.9, 59.5, 13.9; HRMS (ESI) *m*/*z*: calcd [M + H]^+^ for C_16_H_20_NO_7_^+^, 338.1234; found [M + H]^+^, 338.1221.

#### Diethyl 2-((4-Chlorophenyl)carbamoyl)malonate (**15**)

The title compound was prepared following the general procedure method A. The crude material was purified by column chromatography (*n*-hexane/ethyl acetate 1:15%) to give the product as pale-yellow solid (65.9 mg, 84% yield); ^1^H NMR (400 MHz, CDCl_3_): δ 9.38 (br s, 1H, −N*H*), 7.56–7.49 (m, 2H), 7.34–7.28 (m, 2H), 4.44 (s, 1H), 4.31 (q, *J* = 7.1 Hz, 4H), 1.33 (t, *J* = 7.1 Hz, 6H); ^13^C{^1^H} NMR (100 MHz, CDCl_3_): δ 165.7, 159.9, 135.8, 129.9, 129.1, 121.4, 63.1, 59.4, 13.9; HRMS (ESI) *m*/*z*: calcd [M + H]^+^ for C_14_H_17_ClNO_5_^+^, 314.0790; found [M + H]^+^, 314.0784.

#### Diethyl 2-((*tert*-Butylimino)methylene)malonate (**16**)

The title compound was prepared following the general procedure method A. The crude material was purified was purified by chromatography on silica-gel (*n*-hexane/EtOAc 90:10) to provide the title compound **16** (59.1 mg, 98%) as yellow oil. ^1^H NMR (400 MHz, CDCl_3_): δ 4.19 (q, *J* = 7.1 Hz, 4H), 1.54 (s, 9H), 1.26 (t, *J* = 7.1 Hz, 6H); ^13^C{^1^H} NMR (100 MHz, CDCl_3_): δ 165.3 (2C), 63.2, 63.1, 60.0, 30.4, 14.4. HRMS (ESI) *m*/*z*: calcd [M + H]^+^ for C_12_H_20_NO_4_^+^, 242.1386; found [M + H]^+^, 242.1373.

#### Diethyl 2-(*tert*-Butylcarbamoyl)malonate (**17**)

The title compound was prepared following the general procedure method B. The crude material was purified by column chromatography (*n*-hexane/ethyl acetate 1:10%) to give the product as a white solid (27.0 mg, 44% yield). ^1^H NMR (400 MHz, CDCl_3_): δ 7.10 (br s, 1H, −N*H*), 4.29–4.20 (m, 5H), 1.37 (s, 9H), 1.30 (t, *J* = 7.1 Hz, 6H); ^13^C{^1^H} NMR (100 MHz, CDCl_3_): δ 166.1, 161.0, 62.5, 60.0, 51.7, 28.5, 13.9; HRMS (ESI) *m*/*z*: calcd [M + H]^+^ for C_12_H_22_NO_5_^+^, 260.1492; found [M + H]^+^, 260.1479.

#### Diethyl 2-(Cyclohexylcarbamoyl)malonate (**18**)

The title compound was prepared following the general procedure method B. The crude material was purified by column chromatography (*n*-hexane/ethyl acetate 1:10%) to give the product as a white solid (48.5 mg, 68% yield). ^1^H NMR (400 MHz, CDCl_3_): δ 7.17 (d, *J* = 5.2 Hz, 1H), 4.35–4.19 (m, 5H), 3.88–3.74 (m, 1H), 1.96–1.85 (m, 2H), 1.77–1.65 (m, 2H), 1.42–1.18 (m, 12H); ^13^C{^1^H} NMR (100 MHz, CDCl_3_): δ 165.9, 161.1, 62.5, 59.2, 48.5, 32.5, 25.5, 24.4, 13.9; HRMS (ESI) *m*/*z*: calcd [M + H]^+^ for C_14_H_24_NO_5_^+^, 286.1649; found [M + H]^+^, 286.1633.

#### Diethyl 2-(Pentylcarbamoyl)malonate (**19**)

The title compound was prepared following the general procedure method B. The crude material was purified by column chromatography (*n*-hexane/ethyl acetate 1:10%) to give the product as colorless oil (47.8 mg, 70% yield). ^1^H NMR (400 MHz, CDCl_3_): δ 7.29 (br s, 1H, −N*H*), 4.38–4.19 (m, 5H), 3.35-3-25 (m, 2H), 1.60–1.48 (m, 2H), 1.38–1.23 (m, 10H), 0.90 (t, *J* = 6.8 Hz, 3H); ^13^C{^1^H} NMR (100 MHz, CDCl_3_): δ 165.9, 161.9, 62.6, 59.1, 39.9, 28.9, 28.9, 22.3, 14.0, 13.9; HRMS (ESI) *m*/*z*: calcd [M + H]^+^ for C_13_H_24_NO_5_^+^, 274.1649; found [M + H]^+^, 274.1635.

#### *N*-(*tert*-Butyl)-3-oxo-3-phenylpropanamide (**21**)

The title compound was prepared following the general procedure method A. The crude material was purified by column chromatography (*n*-hexane/ethyl acetate 5:15%) to give the product as pale-brown oil (54.2 mg, 95% yield). ^1^H NMR (400 MHz, CDCl_3_): δ 8.04–7.97 (m, 2H), 7.63-7-58 (m, 1H), 7.54–7.45 (m, 2H), 6.86 (br s, 1H, −N*H*), 3.89 (s, 2H), 1.40 (s, 9H); ^13^C{^1^H} NMR (100 MHz, CDCl_3_): δ 196.6, 164.7, 136.3, 134.0, 128.8, 128.6, 51.5, 46.7, 28.7; HRMS (ESI) *m*/*z*: calcd [M + H]^+^ for C_13_H_18_NO_2_^+^, 220.1332; found [M + H]^+^, 220.1320.

#### *N*-Cyclohexyl-3-oxo-3-phenylpropanamide (**22**)

The title compound was prepared following the general procedure method A. The crude material was purified by column chromatography (*n*-hexane/ethyl acetate 5:15%) to give the product as a pale-brown solid (57.0 mg, 93% yield). ^1^H NMR (400 MHz, CDCl_3_): δ 8.03–7.98 (m, 2H), 7.62–7.57 (m, 1H), 7.52–7.45 (m, 3H), 7.00 (br s, 1H, −N*H*), 3.93 (s, 2H), 3.88–3.78 (m, 1H), 1.95–1.85 (m, 2H), 1.75–1.65 (m, 2H), 1.45–1.13 (m, 6H); ^13^C{^1^H} NMR (100 MHz, CDCl_3_): δ 196.4, 164.7, 136.2, 133.9, 128.8, 128.6, 48.3, 46.5, 32.8, 25.5, 24.7; HRMS (ESI) *m*/*z*: calcd [M + H]^+^ for C_15_H_20_NO_2_^+^, 246.1488; found [M + H]^+^, 246.1480.

#### 3-Oxo-*N*-pentyl-3-phenylpropanamide (**23**)

The title compound was prepared following the general procedure method A. The crude material was purified by column chromatography (*n*-hexane/ethyl acetate 5:15%) to give the product as pale-yellow oil (53.1 mg, 91% yield). ^1^H NMR (400 MHz, CDCl_3_): δ 8.05–7.98 (m, 2H), 7.65–7.59 (m, 1H), 7.54–7.46 (m, 2H), 7.09 (br s, 1H, −N*H*), 3.95 (s, 2H), 3.33–2.98 (m, 2H), 1.58–1.49 (m, 2H), 1.39–1.27 (m, 4H), 0.93–0.85 (m, 3H); ^13^C{^1^H} NMR (100 MHz, CDCl_3_): δ 196.3, 165.5, 136.2, 134.0, 128.8, 128.6, 45.3, 39.6, 29.9, 29.7, 22.3, 14.0; HRMS (ESI) *m*/*z*: calcd [M + H]^+^ for C_14_H_20_NO_2_^+^, 234.1488; found [M + H]^+^, 234.1476.

#### 2-Acetyl-*N*-(*tert*-butyl)benzamide (**25**)

The title compound was prepared following the general procedure method A. The crude material was purified by column chromatography (*n*-hexane/ethyl acetate 1:10%) to give the product as a white solid (21.9 mg, 40% yield; partial conversion). ^1^H NMR (400 MHz, CDCl_3_): δ 7.70 (d, *J* = 7.5 Hz, 1H), 7.60–7.55 (m, 1H), 7.50–7.45 (m, 3H), 1.70 (s, 3H), 1.05 (s, 9H); ^13^C{^1^H} NMR (100 MHz, CDCl_3_): δ 201.6, 168.5, 137.2, 132.2, 130.9, 129.7, 127.9, 127.4, 52.0, 29.2, 28.6; HRMS (ESI) *m*/*z*: calcd [M + H]^+^ for C_13_H_18_NO_2_^+^, 220.1332; found [M + H]^+^, 220.1322.

#### 2-Acetyl-*N*-cyclohexylbenzamide (**26**)

The title compound was prepared following the general procedure method A. The crude material was purified by column chromatography (*n*-hexane/ethyl acetate 1:10%) to give the product as a white solid (30.7 mg, 50% yield; partial conversion); ^1^H NMR (400 MHz, CD_3_OD): δ 7.57 (d, *J* = 7.8 Hz, 1H), 7.53–7.45 (m, 3H), 7.38 (dt, *J* = 7.8, 1.3 Hz, 1H), 3.43–3.33 (m, 1H), 1.95–1.85 (m, 2H), 1.75–1.55 (m, 5H), 1.45–1.13 (m, 6H); ^13^C{^1^H} NMR (100 MHz, CD_3_OD): δ 167.1, 148.5, 132.00, 131.2, 129.0., 122.1, 89.1, 29.9, 29.8, 26.0, 25.1; HRMS (ESI) *m*/*z*: calcd [M + H]^+^ for C_15_H_20_NO_2_^+^, 246.1488; found [M + H]^+^, 246.1469.

#### 2-Acetyl-*N*-pentylbenzamide (**27**)

The title compound was prepared following the general procedure method A. The crude material was purified by column chromatography (*n*-hexane/ethyl acetate 1:10%) to give the product as colorless oil (17.5 mg, 30% yield; partial conversion). ^1^H NMR (400 MHz, CDCl_3_): δ 7.68 (d, *J* = 7.5 Hz, 1H), 7.57–7.53 (m, 2H), 7.49–7.41 (m, 1H), 3.4 (ddd, *J* = 14.1, 10.3, 5.6 Hz, 1H), 3.25 (ddd, *J* = 14.1, 10.2, 5.7 Hz, 1H), 2.87 (br s, 1H), 1.82–1.55 (m, 5H), 1.45–1.21 (m, 4H), 0.90 (t, *J* = 6.9 Hz, 3H); ^13^C{^1^H} NMR (100 MHz, CDCl_3_): δ 166.8, 148.0, 132.2, 129.5, 123.2, 121.5, 88.8, 38.7, 29.7, 29.6, 29.0, 24.2, 22.4, 14.0; HRMS (ESI) *m*/*z*: calcd [M + H]^+^ for C_14_H_20_NO_2_^+^, 234.1488; found [M + H]^+^, 234.1477.

#### *N*-(*tert*-Butyl)-2-cyanoacetamide (**29**)

The title compound was prepared following the general procedure method A. The crude material was purified by column chromatography (*n*-hexane/ethyl acetate 1:10%) to give the product as colou=rless oil (5.3 mg, 15% yield). ^1^H NMR (400 MHz, CDCl_3_): δ 5.95 (br s, 1H, −N*H*), 3.30 (s, 2H), 1.35 (s, 9H); ^13^C{^1^H} NMR (100 MHz, CDCl_3_): δ 160.0, 115.0, 51.5, 28.0, 26.6; HRMS (ESI) *m*/*z*: calcd [M + H]^+^ for C_7_H_13_N_2_O^+^, 141.1022; found [M + H]^+^, 141.1021.

### General One-Pot Two-Steps Procedure for the Preparation of Compounds (**30–35** and **37–40**) (Method C)

To a 4 mL colorless screw-cap glass vial equipped with a magnetic stir bar were added the isocyanide (0.25 mmol), Na_2_CO_3_ (0.375 mmol, 1.5 equiv), *fac*-Ir(ppy)_3_ (0.0025 mmol, 1% mol), diethylbromomalonate (0.375 mmol, 1.5 equiv), and DABCO (0.125 mmol, 0.5 equiv). Then, 2.5 mL MeCN (0.1 M) and H_2_O 95 μL (20 equiv) were added into the reaction vial via a syringe. The resulting mixture was purged with nitrogen and then stirred under 30 W blue LED irradiation at room temperature for 20 h. After turning-off irradiation, the nucleophile (0.25 mmol) was added, and the reaction mixture was stirred at room temperature for additional 20–40 h. Then, the reaction mixture was poured into water, extracted with EtOAc (3 times), the collected organic layers were washed with brine (1 time), dried over dry Na_2_SO_4_, and evaporated under vacuum. The reaction crude was purified by chromatography on silica-gel.

#### Diethyl 2-((*tert*-Butylamino)-((4-methoxyphenyl)amino)methylene)-malonate (**30**)

The title compound was prepared following the general procedure method C. The crude material was purified by column chromatography (*n*-hexane/EtOAc 80:20) to give the product as pale-brown oil (72.9 mg, 88% yield). ^1^H NMR (400 MHz, DMSO-*d*_6_): δ 8.25–8.18 (m, 2H), 7.05–6.99 (m, 1H), 6.89–6.81 (m, 1H), 3.84–3.69 (m, 7H), 1.32 (s, 9H), 1.09 (t, *J* = 7.1 Hz, 6H); ^13^C{^1^H} NMR (100 MHz, DMSO-*d*_6_): δ 168.3, 162.8, 156.0, 134.2, 123.9, 114.2, 80.9, 58.6, 55.7, 54.3, 30.4, 14.8; HRMS (ESI) *m*/*z*: calcd [M + H]^+^ for C_19_H_29_N_2_O_5_^+^, 365.2071; found [M + H]^+^, 365.2083.

#### Diethyl 2-((*tert*-Butylamino)-((4-chlorophenyl)amino)methylene)-malonate (**31**)

The title compound was prepared following the general procedure method C. The crude material was purified by column chromatography (*n*-hexane/EtOAc 80:20) to give the product as pale-brown oil (55.3 mg, 60% yield). ^1^H NMR (400 MHz, DMSO-*d*_6_): δ 8.23–8.16 (m, 2H), 7.38–7.24 (m, 2H), 6.89–6.83 (m, 2H), 3.75 (q, *J* = 7.1 Hz, 4H), 1.33 (s, 9H), 1.04 (t, *J* = 7.1 Hz, 6H); ^13^C{^1^H} NMR (100 MHz, DMSO-*d*_6_): δ 168.3, 168.0, 134.2, 123.9, 122.0, 114.2, 80.9, 58.8, 54.3, 30.4, 14.8; HRMS (ESI) *m*/*z*: calcd [M + H]^+^ for C_18_H_26_ClN_2_O_4_^+^, 369.1575; found [M + H]^+^, 369.1565.

#### Diethyl 2-((*tert*-Butylamino)-((4-methoxybenzyl)amino)methylene)-malonate (**32**)

The title compound was prepared following the general procedure method C. The crude material was purified by column chromatography (CH_2_Cl_2_/MeOH 99:1) to give the product as a white solid (85.2 mg, 90% yield): mp 158–160 °C; ^1^H NMR (400 MHz, CDCl_3_); δ 8.05 (br s, 1H), 7.46 (br s, 1H), 7.22–7.15 (m, 2H), 6.90–6.82 (m, 2H), 4.33 (d, *J* = 5.1 Hz, 2H), 4.15 (q, *J* = 7.1 Hz, 4H), 3.80 (s, 3H), 1.34 (s, 9H), 1.27 (t, *J* = 7.1 Hz, 6H); ^13^C{^1^H} NMR (100 MHz, CDCl_3_): δ 170.3, 166.4, 159.2, 130.2, 129.2, 114.2, 80.7, 59.7, 55.3, 54.7, 50.7, 30.6, 14.4; HRMS (ESI) *m*/*z*: calcd [M + H]^+^ for C_20_H_31_N_2_O_5_^+^, 379.2227; found [M + H]^+^, 379.2224.

#### Diethyl 2-((*tert*-Butylamino)-(cyclohexylamino)methylene)malonate (**33**)

The title compound was prepared following the general procedure method C. The crude material was purified by column chromatography (CH_2_Cl_2_/MeOH 97:3) to give the product as a white solid (80.0 mg, 94% yield): mp 154–156 °C; ^1^H NMR (400 MHz, CDCl_3_): δ 8.01 (br s, 1H), 7.86 (d, *J* = 8.6 Hz, 1H), 4.15 (q, *J* = 7.1 Hz, 4H), 3.53–3.44 (m, 1H), 2.00–1.93 (m, 2H), 1.78–1.70 (m, 2H), 1.35 (s, 9H), 1.32–1.13 (m, 12H); ^13^C{^1^H} NMR (100 MHz, CDCl_3_): δ 170.9, 166.0, 81.6, 59.6, 55.5, 54.8, 33.3, 30.4, 25.5, 25.0, 14.4; HRMS (ESI) *m*/*z*: calcd [M + H]^+^ for C_18_H_33_N_2_O_4_^+^, 341.2434; found [M + H]^+^, 341.2421.

#### Diethyl 2-((*tert*-Butylamino)-(morpholino)methylene)malonate (**34**)

The title compound was prepared following the general procedure method C. The crude material was purified by column chromatography (CH_2_Cl_2_/MeOH 95:5) to give the product as a white solid (72.2 mg, 88% yield): mp 156–158 °C; ^1^H NMR (400 MHz, CDCl_3_): δ 6.12 (br s, 1H), 4.10 (d, *J* = 7.1 Hz, 4H), 3.80–3.71 (m, 4H), 3.42–3.30 (m, 4H), 1.35 (s, 9H), 1.27 (t, *J* = 7.1 Hz, 6H); ^13^C{^1^H} NMR (101 MHz, CDCl_3_): δ 168.0 (2C), 77.2, 66.2, 59.3, 56.0, 49.8, 30.3, 14.7; HRMS (ESI) *m*/*z*: calcd [M + H]^+^ for C_16_H_29_N_2_O_5_^+^, 329.2071; found [M + H]^+^, 329.2058.

#### Diethyl 2-((*tert*-Butylamino)-(piperidin-1-yl)methylene)malonate (**35**)

The title compound was prepared following the general procedure method C. The crude material was purified by column chromatography (CH_2_Cl_2_/MeOH 95:5) to give the product as a white solid (40.8 mg, 50% yield): mp 155–157 °C; ^1^H NMR (400 MHz, CDCl_3_): δ 5.69 (br s, 1H), 4.10 (q, *J* = 7.1 Hz, 4H), 3.40–3.33 (m, 4H), 1.37 (s, 9H), 1.32–1.14 (m, 12H); ^13^C{^1^H} NMR (101 MHz, CDCl_3_): δ 167.7 (2C), 77.3, 58.7, 55.8, 50.2, 30.2, 25.3, 24.3, 14.8; HRMS (ESI) *m*/*z*: calcd [M + H]^+^ for C_17_H_31_N_2_O_4_^+^, 327.2278; found [M + H]^+^, 327.2269.

#### Diethyl 2-((Cyclohexylamino)-((4-methoxyphenyl)amino)methylene)-malonate (**37**)

The title compound was prepared following the general procedure method C. The crude material was purified by column chromatography (hexane/EtOAc 80:20) to give the product as pale-brown oil (40.8 mg, 75% yield). ^1^H NMR (400 MHz, CDCl_3_): δ 10.70 (br s, 1H), 9.34 (d, *J* = 8.6 Hz, 1H), 7.04–6.96 (m, 2H), 6.82–6.73 (m, 2H), 4.10 (q, *J* = 7.1 Hz, 4H), 3.73 (s, 3H), 2.92–2.77 (m, 1H), 1.67–1.56 (m, 2H), 1.55–1.44 (m, 2H), 1.37–1.15 (m, 10H), 1.10–1.00 (m, 2H); ^13^C{^1^H} NMR (101 MHz, CDCl_3_): δ 171.6, 162.8, 157.0, 133.0, 124.6, 114.4, 78.6, 63.2, 59.7, 55.5, 52.2, 42.4, 32.6, 25.4, 24.3, 14.4, 13.9; HRMS (ESI) *m*/*z*: calcd [M + H]^+^ for C_21_H_31_N_2_O_5_^+^, 391.2227; found [M + H]^+^, 391.2213.

#### Diethyl 2-(((4-Methoxyphenyl)amino)-(pentylamino)methylene)-malonate (**38**)

The title compound was prepared following the general procedure method C. The crude material was purified by column chromatography (hexane/EtOAc 80:20) to give the product as pale brown oil (40.8 mg, 80% yield). ^1^H NMR (400 MHz, CDCl_3_): δ ^1^H NMR (400 MHz, CDCl_3_): δ 10.84 (br s, 1H), 9.58 (br s, 1H), 7.07–6.99 (m, 2H), 6.88–6.80 (m, 2H), 4.18 (q, *J* = 7.1 Hz, 4H), 3.80 (s, 3H), 2.74 (dt, *J* = 7.0, 5.2 Hz, 2H), 1.50–1.38 (m, 2H), 1.31 (t, *J* = 7.1 Hz, 6H), 1.25–1.12 (m, 4H), 0.83 (t, *J* = 6.9 Hz, 3H). ^13^C{^1^H} NMR (101 MHz, CDCl_3_): δ 171.5, 163.6, 156.8, 133.0, 124.5, 114.4, 78.1, 59.7, 55.5, 45.4, 29.4, 28.8, 22.2, 14.4, 13.9; HRMS (ESI) *m*/*z*: calcd [M + H]^+^ for C_20_H_31_N_2_O_5_^+^, 379.2227; found [M + H]^+^, 379.2214.

#### Ethyl 5-(*tert*-Butylamino)-3-oxo-2-phenyl-2,3-dihydro-1*H*-pyrazole-4-carboxylate (**39**)

The title compound was prepared following the general procedure method C. The crude material was purified by column chromatography (CH_2_Cl_2_/MeOH 98:2) to give the product as an orange solid (50.0 mg, 66% yield): mp 80–82 °C. ^1^H NMR (400 MHz, CDCl_3_): δ 1H NMR (400 MHz, DMSO-*d*_6_): δ 7.80 (br s, 1H), 7.65–7.55 (m, 2H), 7.48–7.40 (m, 2H), 7.20–7.10 (m, 1H), 4.18 (q, *J* = 6.9 Hz, 2H), 1.30–1.18 (m, 12H); ^13^C{^1^H} NMR (101 MHz, DMSO-*d*_6_): δ 165.8, 163.1, 151.6, 139.5, 129.0 (2C), 120.1, 59.0, 52.0, 50.3, 29.5, 15.0; HRMS (ESI) *m*/*z*: calcd [M + H]^+^ for C_16_H_22_N_3_O_3_^+^, 304.1655; found [M + H]^+^, 304.1655.

#### Diethyl 2-(1-(*tert*-Butyl)-1*H*-tetrazol-5-yl)malonate (**40**)

The title compound was prepared following the general procedure method C. The crude material was purified by column chromatography (hexane/EtOAc 8:2) to give the product as a white solid (35.5 mg, 50% yield): mp 50–51 °C; ^1^H NMR (400 MHz, CDCl_3_): δ 4.29–4.20 (m, 5H), 1.37 (s, 9H), 1.30 (t, *J* = 7.1 Hz, 6H); ^13^C{^1^H} NMR (101 MHz, CDCl_3_): δ 166.1, 161.0, 62.5, 60.0, 51.7, 28.5, 13.9; MS (ESI) *m*/*z*: calcd [M + H]^+^ for C_12_H_21_N_4_O_4_^+^, 285.15; found [M + H]^+^, 285.00; HRMS analysis for compound **40** gave peaks derived from fragmentation of tetrazole ring, as described in literature.^24^

### Stern–Volmer Fluorescence Quenching Experiments

Fluorescence titration experiments were performed at 20 °C on a FP-8300 spectrofluorometer (Jasco) equipped with a Peltier temperature controller system (Jasco PCT-818). A sealed quartz cuvette with a path length of 1 cm was used. Titrations were carried out by stepwise addition of diethyl bromomalonate (**10**) or DABCO to the cell containing a fixed concentration of *fac*-Ir(ppy)_3_ solution (200 μM) in MeCN/H_2_O (4:1). The Ir(III) complex was excited at 450 nm, and emission spectra were recorded between 460 and 650 nm at 100 nm/min scan speed. Both excitation and emission slit widths were set at 5 nm.

## Supporting Information Available

HRMS spectra for TEMPO quenching experiments; references for NMR data of known compounds; and copies of ^1^H and ^13^C NMR spectra for all the compounds reported in the experimental section (PDF)
