# Supplementary material for: Visible-Light Photocatalytic Functionalization of Isocyanides for the Synthesis of Secondary Amides and Ketene Aminals
Source: J Org Chem. 2020 Oct 19;85(21):14077–86. doi: 10.1021/acs.joc.0c01946 (PMC8015228; doi:10.1021/acs.joc.0c01946)

SUPPLEMENTARY INFORMATION

Visible-Light Photocatalytic Functionalization of Isocyanides for the Synthesis of Secondary Amides and Ketene Aminals

Rolando Cannalire,[a] Jussara Amato, [a] Vincenzo Summa,[a] Ettore Novellino,[a] Gian Cesare Tron,[b] and Mariateresa Giustiniano*[a]

[a] *Department of Pharmacy, University of Naples Federico II, via D. Montesano 49, 80131, Napoli, Italy*

[b] *Department of Pharmaceutical Sciences, University of Piemonte Orientale, Largo Donegani 2, 28100, Novara, Italy*

Contents:

- HRMS spectra for control experiments S2
- Copies of ^{1}H and ^{13}C spectra S4

HRMS spectra for control experiments

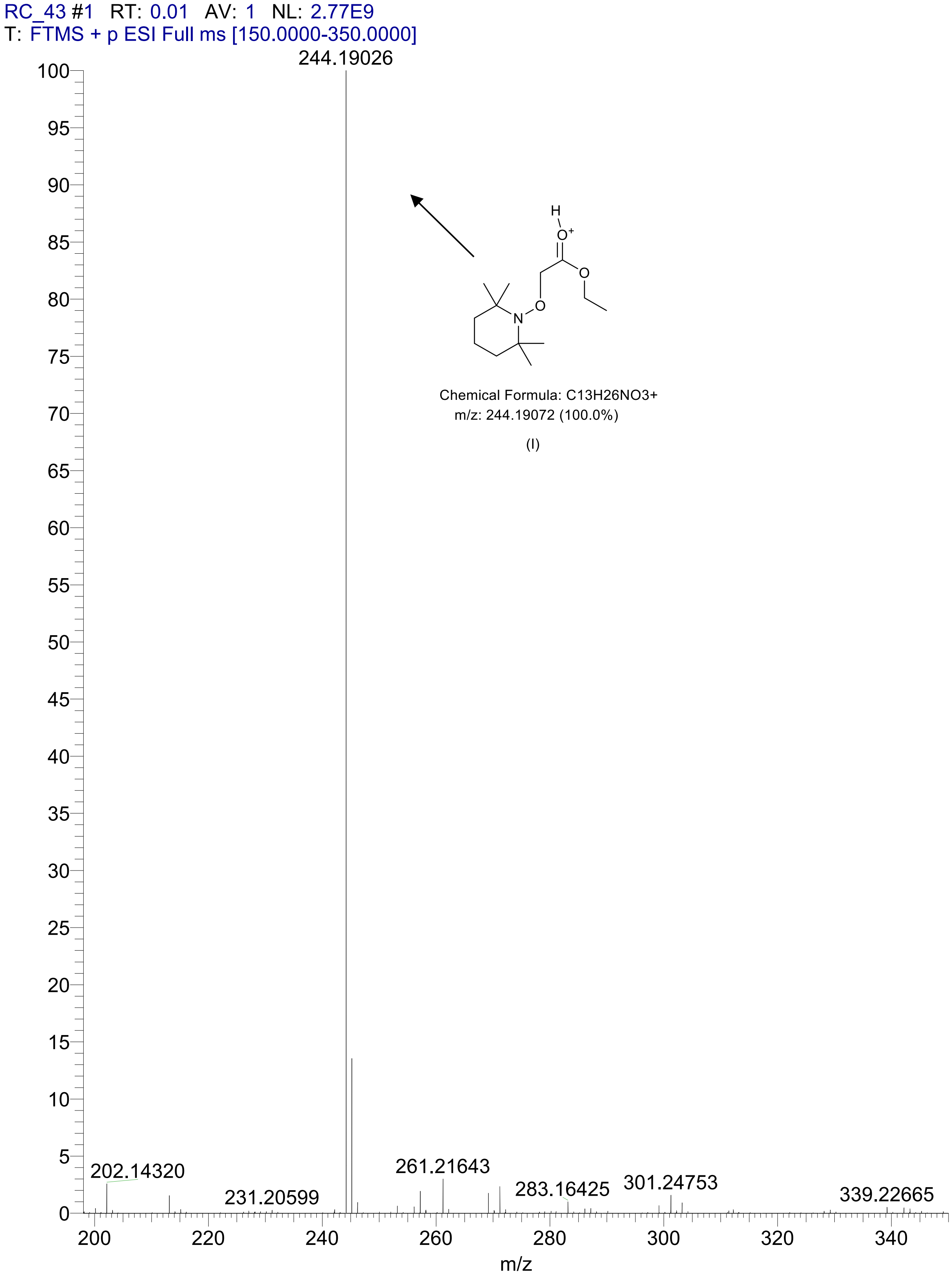

Figure S1. TEMPO adduct with an alkyl radical intermediate.

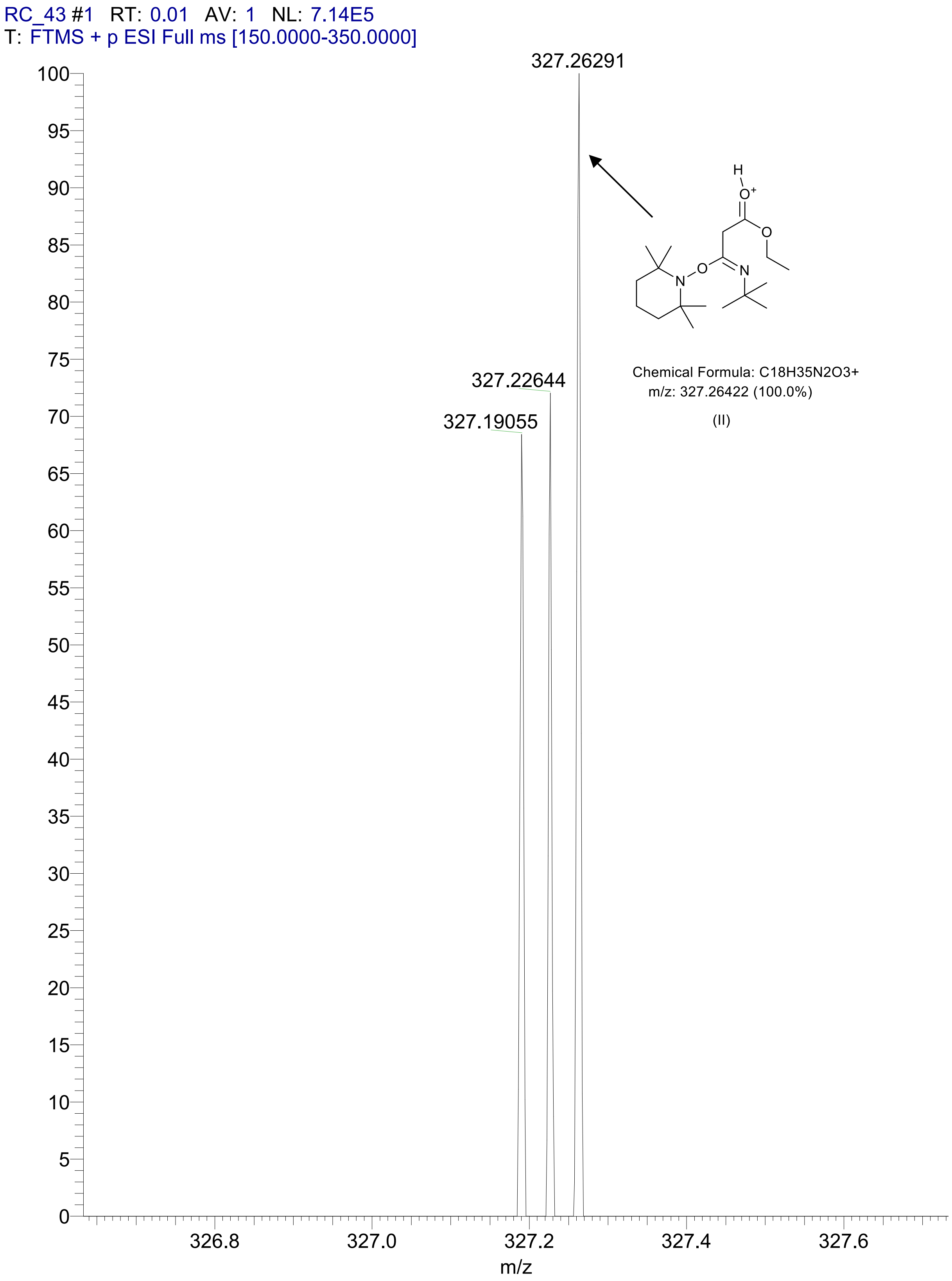

Figure S2. TEMPO adduct with an imidoyl radical intermediate.

The NMR data of known compounds (**3**, **4**, **5**, **7**, **8**, **9**, **12**, **15**, **17**, **18**, **21**, **22**, **23**, **25**, **26** and **29**) are consistent to literature data.[1-15]

References

1 S. Rohe, T. McCallum, A. O. Morris and L. Barriault, *J. Org. Chem.*, 2018, **83**, 10015–10024.

2 Z. W. Wicks and K.-J. Wu, *J. Org. Chem.*, 2002, **45**, 2446–2448.

3 1992 P. Jakobsen, A. Kanstrup, Lundbeck, M. Jane, WO Pat. 9201672 A1 19920206, .

4 R. K. Rapolu, B. Nabamukul, S. R. Bommineni, R. Potham, N. Mulakayala and S. Oruganti, *RSC Adv.*, 2013, **3**, 5332–5337.

5 S. Sumino, A. Fusano, T. Fukuyama and I. Ryu, *Synlett*, 2012, **23**, 1331–1334.

6 WO 2009117659 A1 20090924, 2009.

7 S. Pelliccia, A. I. Alfano, P. Luciano, E. Novellino, A. Massarotti, G. C. Tron, D. Ravelli and M. Giustiniano, *J. Org. Chem.*, 2020, **85**, 1981–1990.

8 H. Stamm and L. Schneider, *Chem. Ber.*, 1975, **108**, 500–510.

9 A. P. Gurevich, A. I.; Kolosov, M. N.; Konnova, G. S.; Nametkina, L. N.; Prudnikova, *Zhurnal Org. Khimii*, 1969, **5**, 1766–1771.

10 Z. W. Wicks and P. P. Patel, *J. Org. Chem.*, 1981, **46**, 4068–4069.

11 D. J. Woodman and A. I. Davidson, *J. Org. Chem.*, 1973, **38**, 4288–4295.

12 J. K. Vandavasi, C.-T. Hsiao, W.-P. Hu, S. S. K. Boominathan and J.-J. Wang, *European J. Org. Chem.*, 2015, **2015**, 3171–3177.

13 A. G. Neo, J. Delgado, C. Polo, S. Marcaccini and C. F. Marcos, *Tetrahedron Lett.*, 2005, **46**, 23–26.

14 T. Nishio and H. Yamamoto, *J. Heterocycl. Chem.*, 1995, **32**, 883–891.

15 H.-R. Müller and M. Seefelder, *Justus Liebigs Ann. Chem.*, 1969, **728**, 88–98.

^{1}H NMR
400 MHz, $CDCl_3$

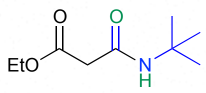

3

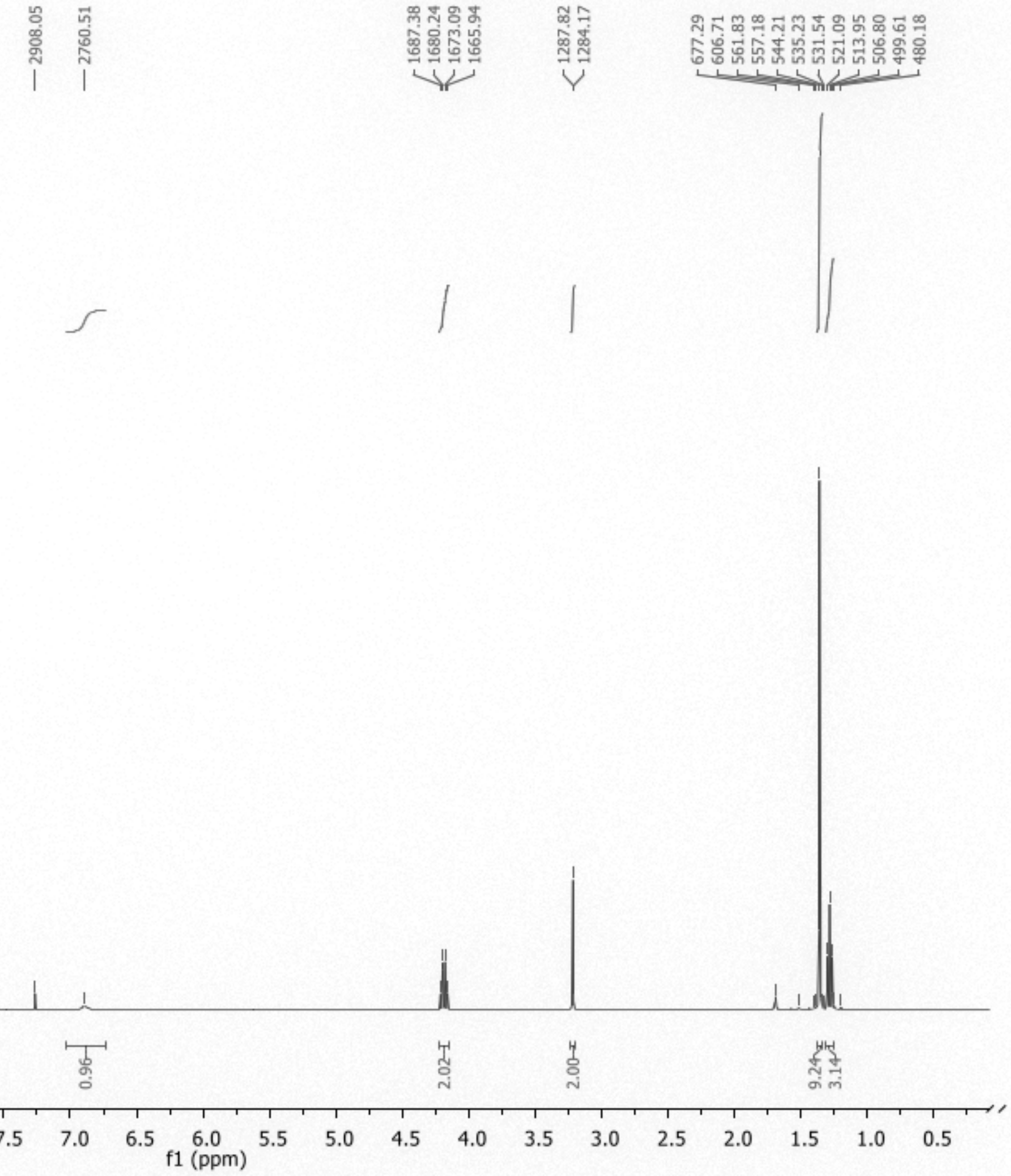

^{13}C NMR
100 MHz, $CDCl_3$

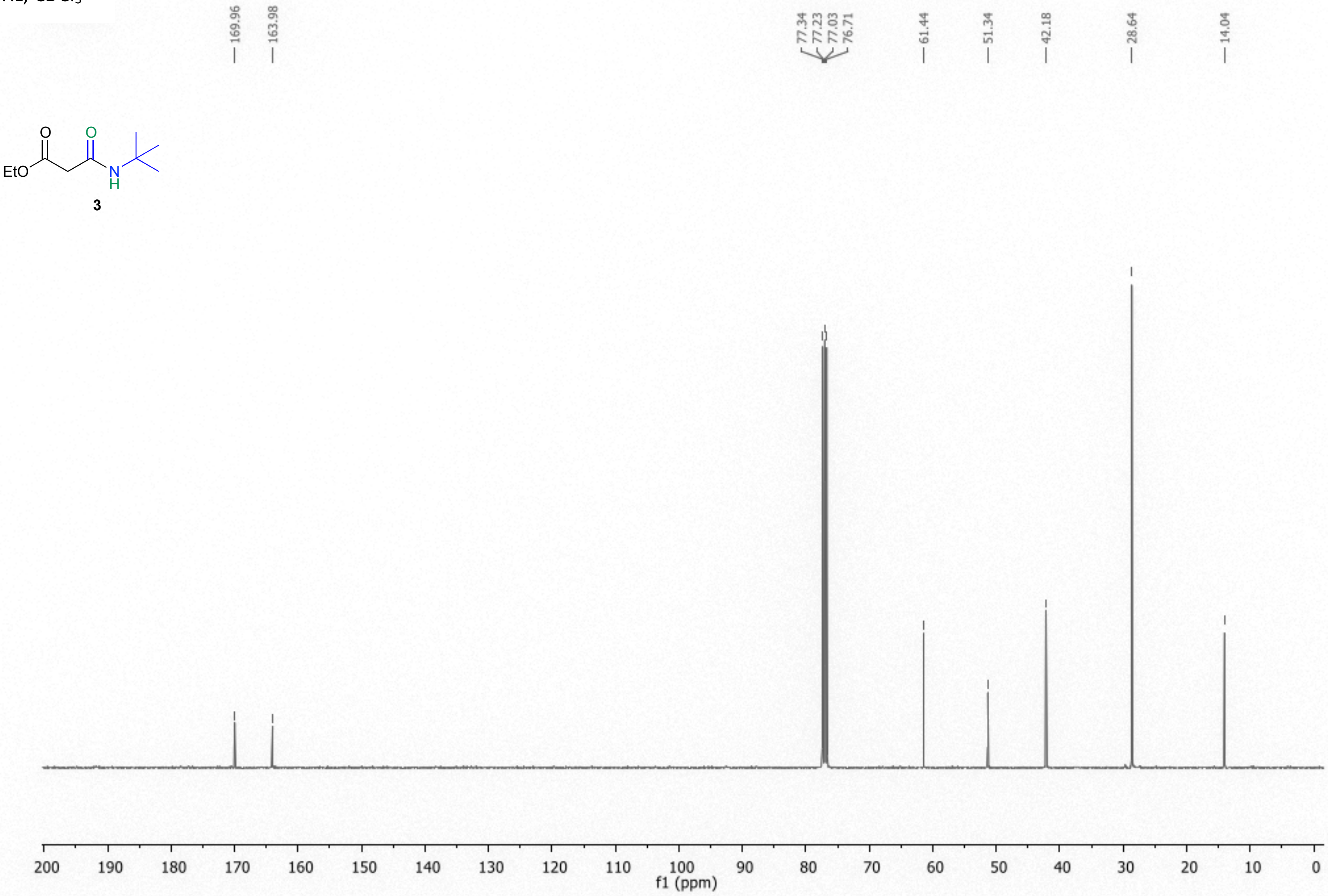

^{1}H NMR
400 MHz, $CDCl_3$

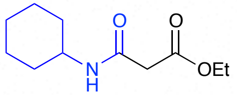

4

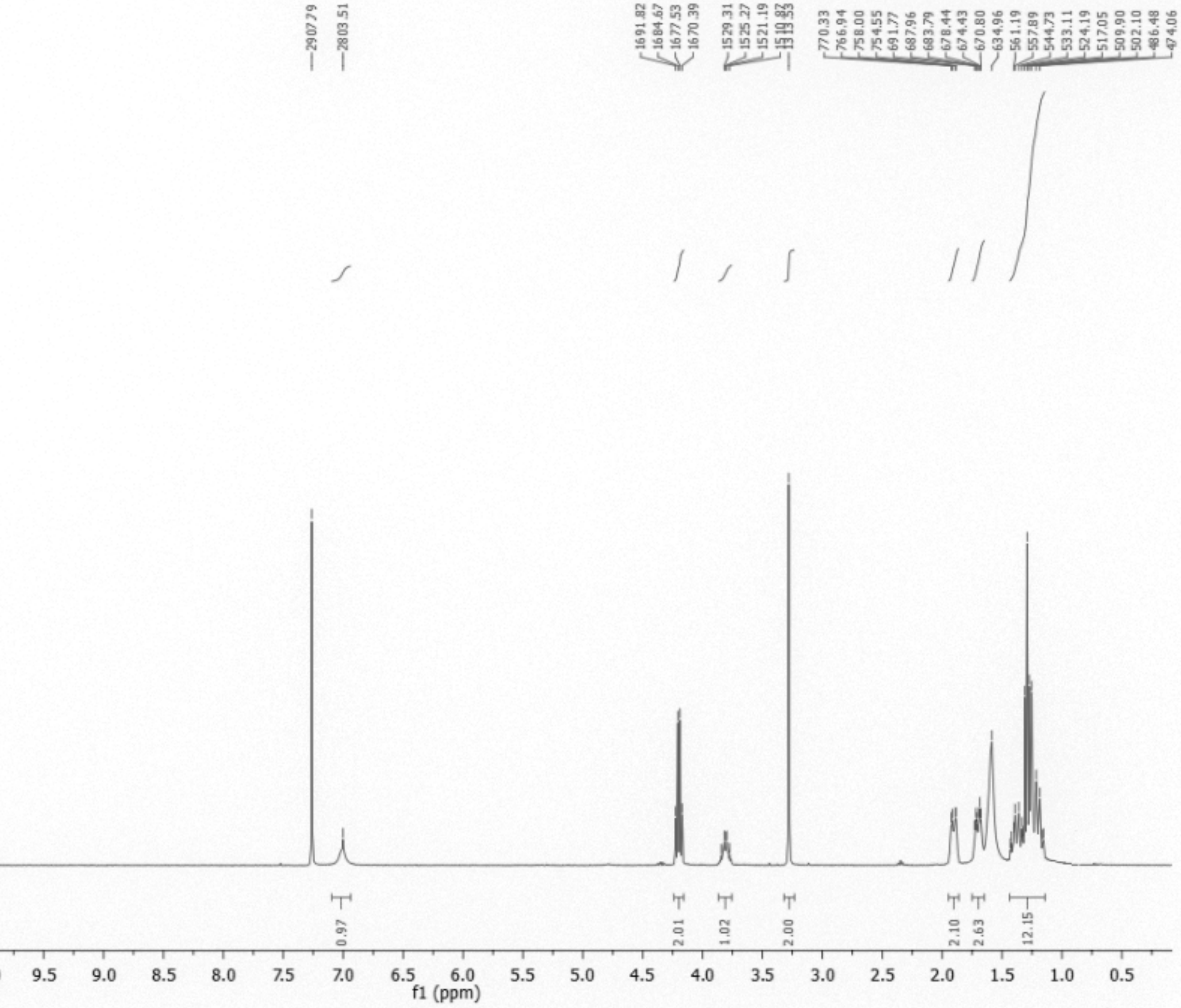

^{13}C NMR
100 MHz, $CDCl_3$

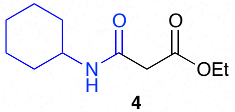

4

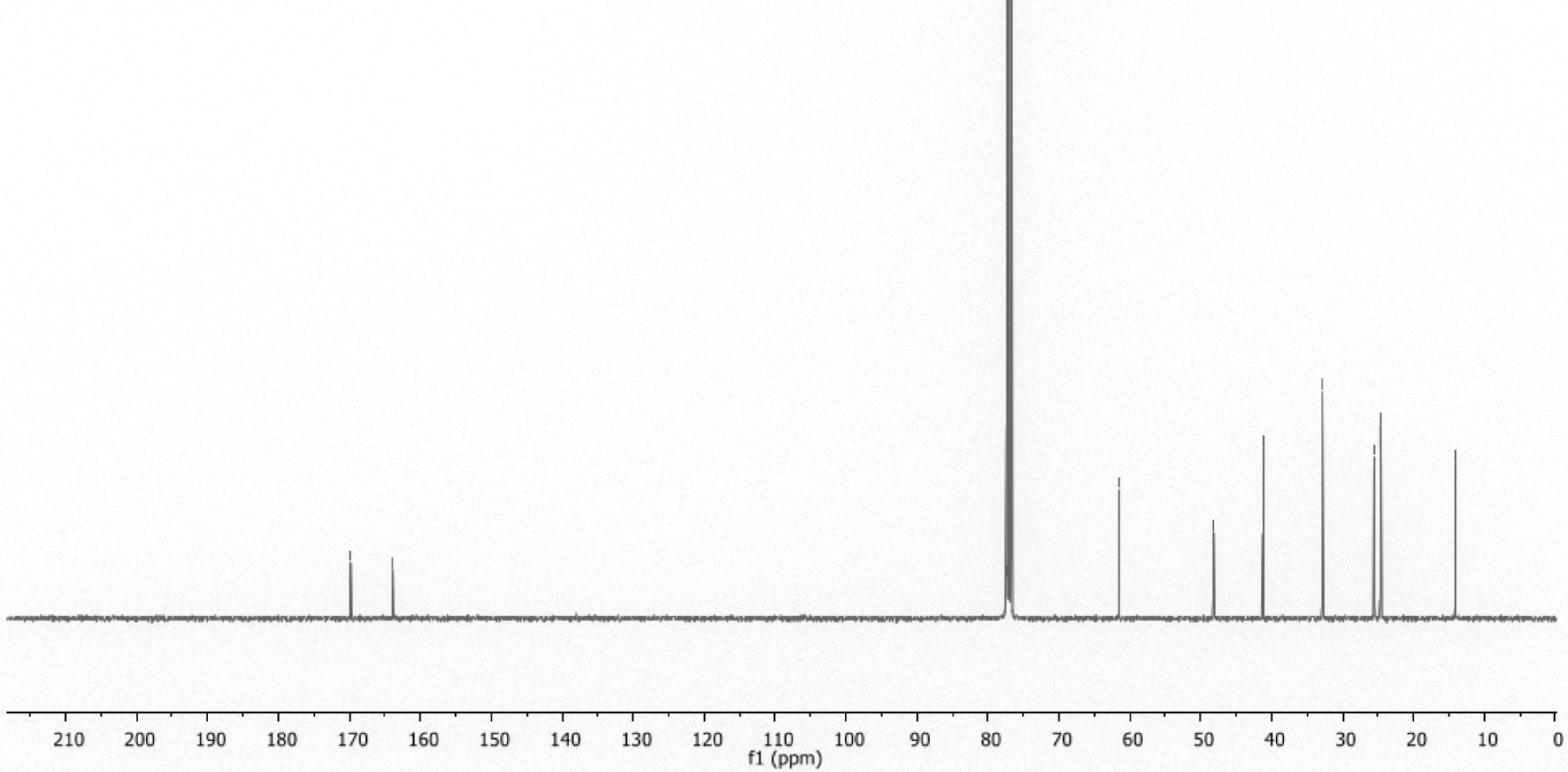

^{1}H NMR
400 MHz, $CDCl_3$

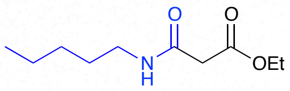

5

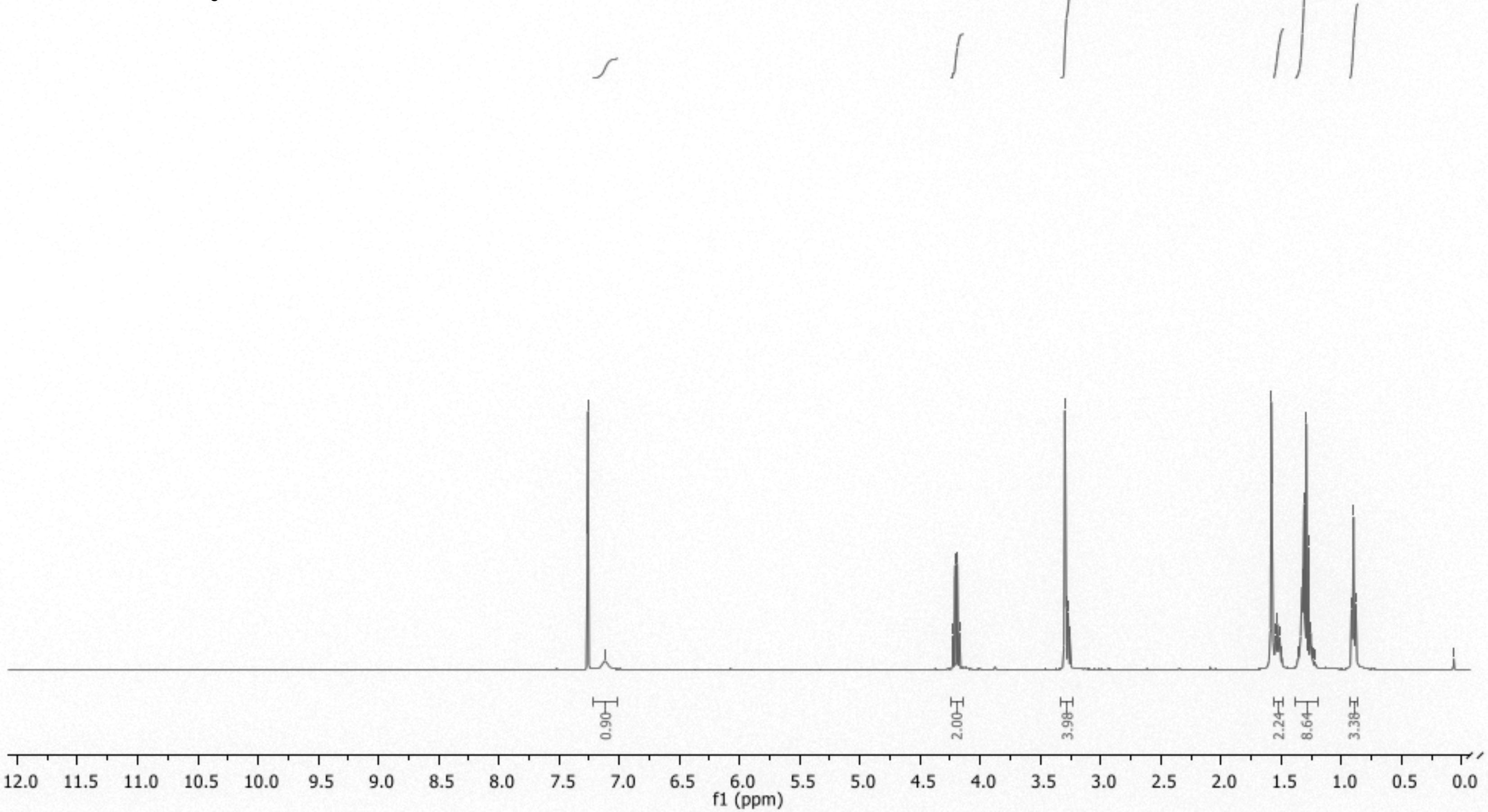

^{13}C NMR
100 MHz, $CDCl_3$

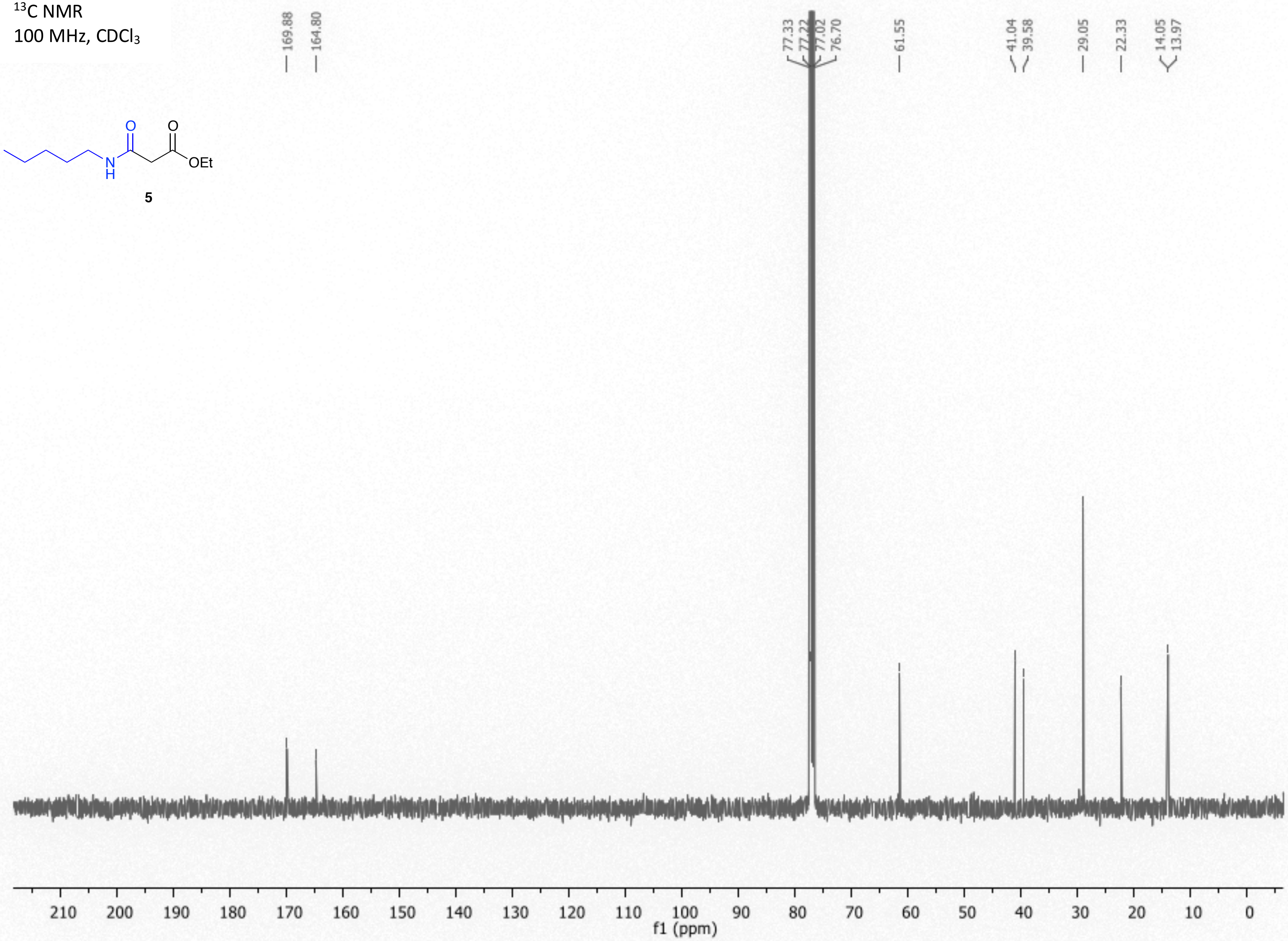

5

^{1}H NMR
400 MHz, $CDCl_3$

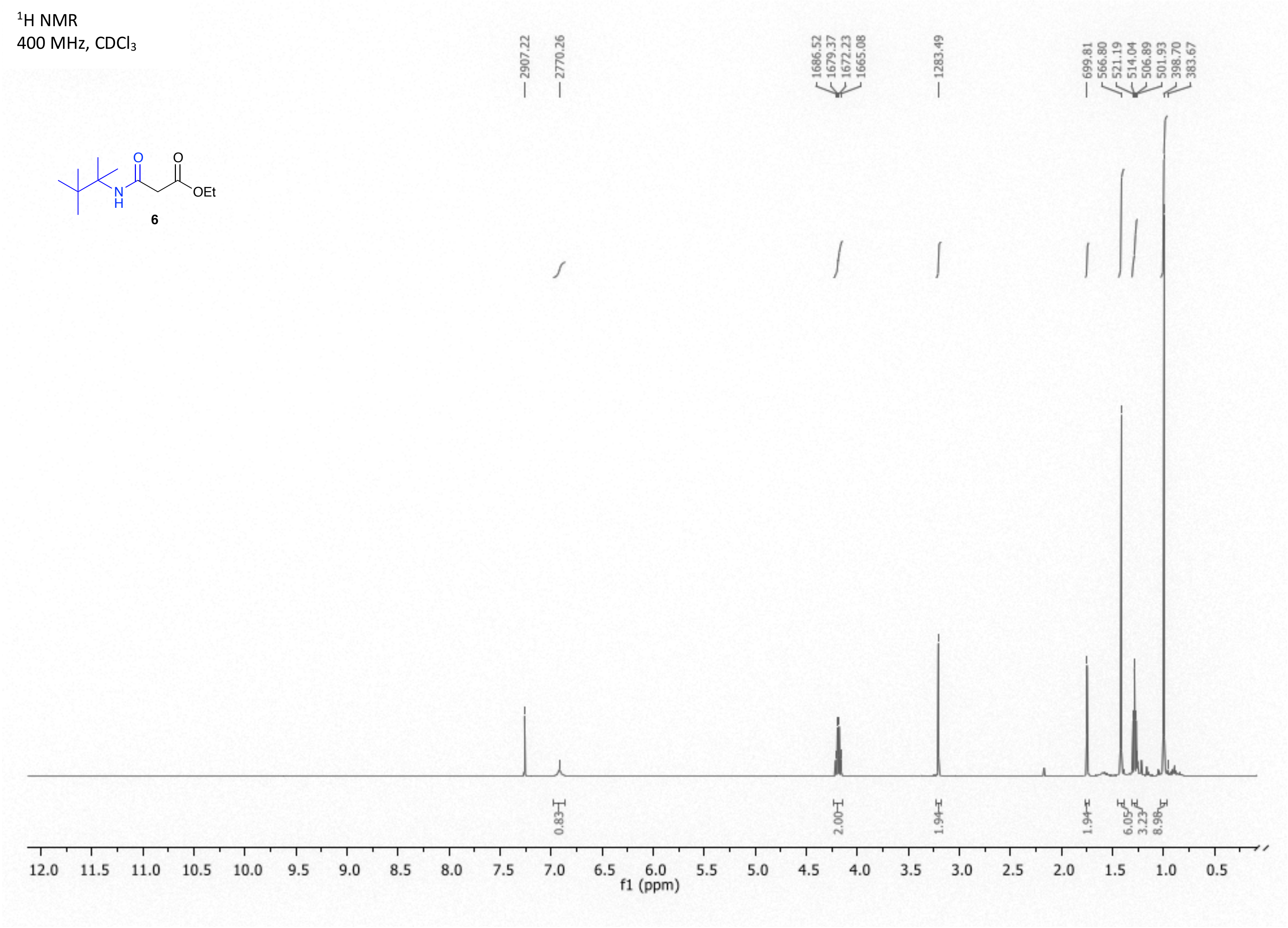

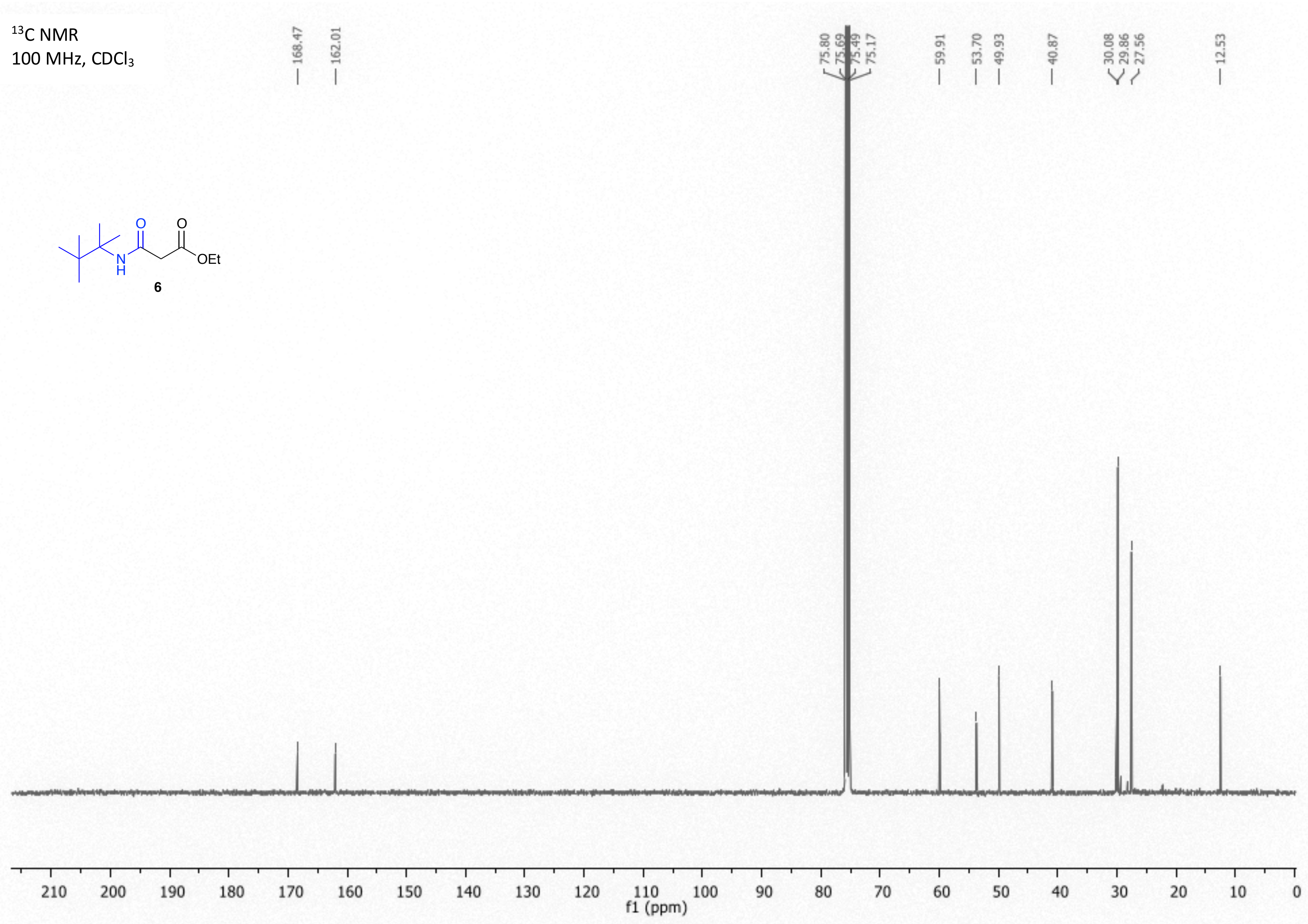

13C NMR
100 MHz, CDCl3
6
OEt
168.47
162.01
75.80
75.69
75.49
75.17
59.91
53.70
49.93
40.87
30.08
29.86
27.56
12.53
210 200 190 180 170 160 150 140 130 120 110 100 90 80 70 60 50 40 30 20 10 0
f1 (ppm)

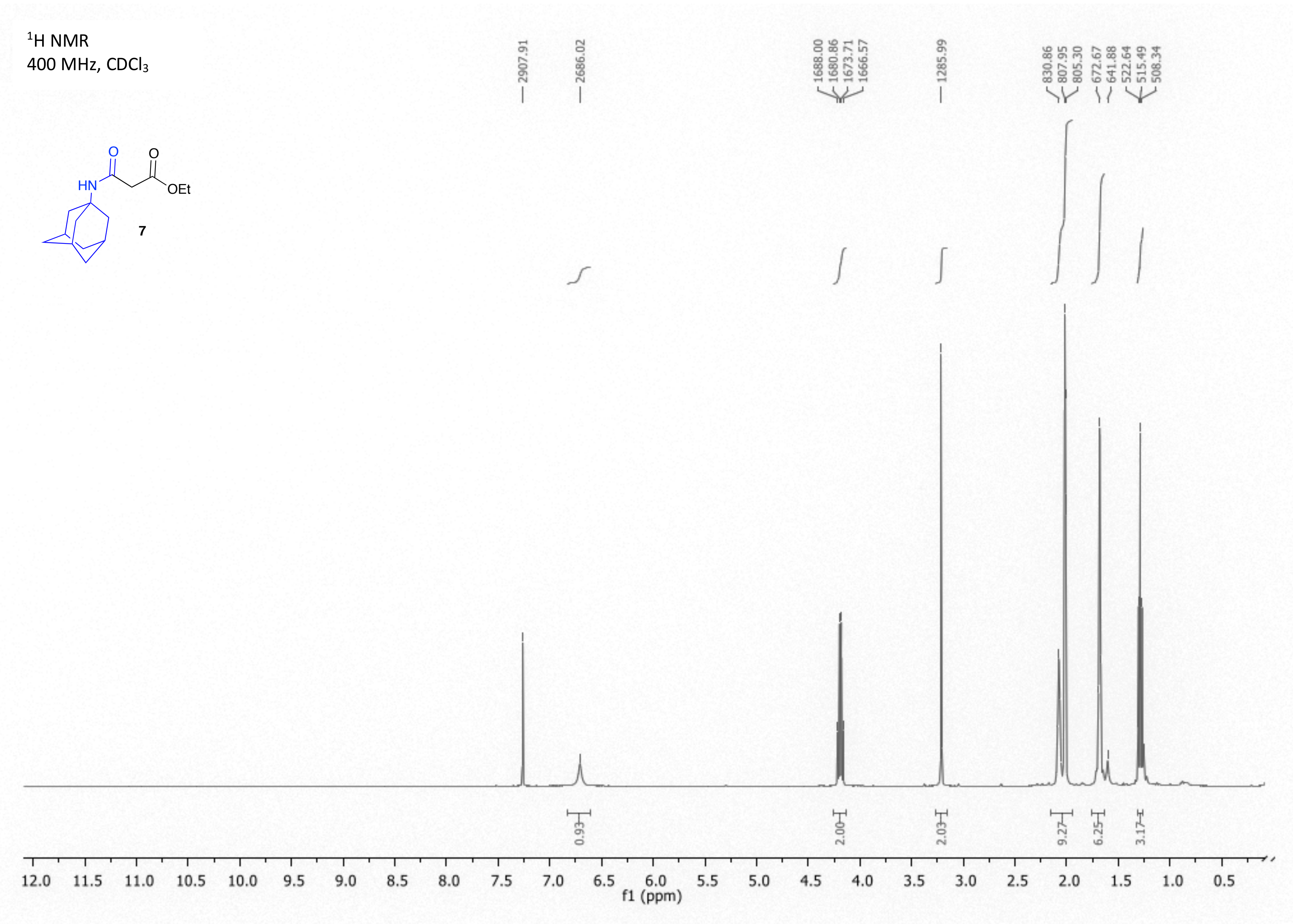

1H NMR
400 MHz, CDCl3
O
O
HN
OEt
7
2907.91
2686.02
1688.00
1680.86
1673.71
1666.57
1285.99
830.86
807.95
805.30
672.67
641.88
522.64
515.49
508.34
0.93
2.00
2.03
9.27
6.25
3.17
12.0 11.5 11.0 10.5 10.0 9.5 9.0 8.5 8.0 7.5 7.0 6.5 6.0 5.5 5.0 4.5 4.0 3.5 3.0 2.5 2.0 1.5 1.0 0.5
f1 (ppm)

^{13}C NMR
100 MHz, $CDCl_3$

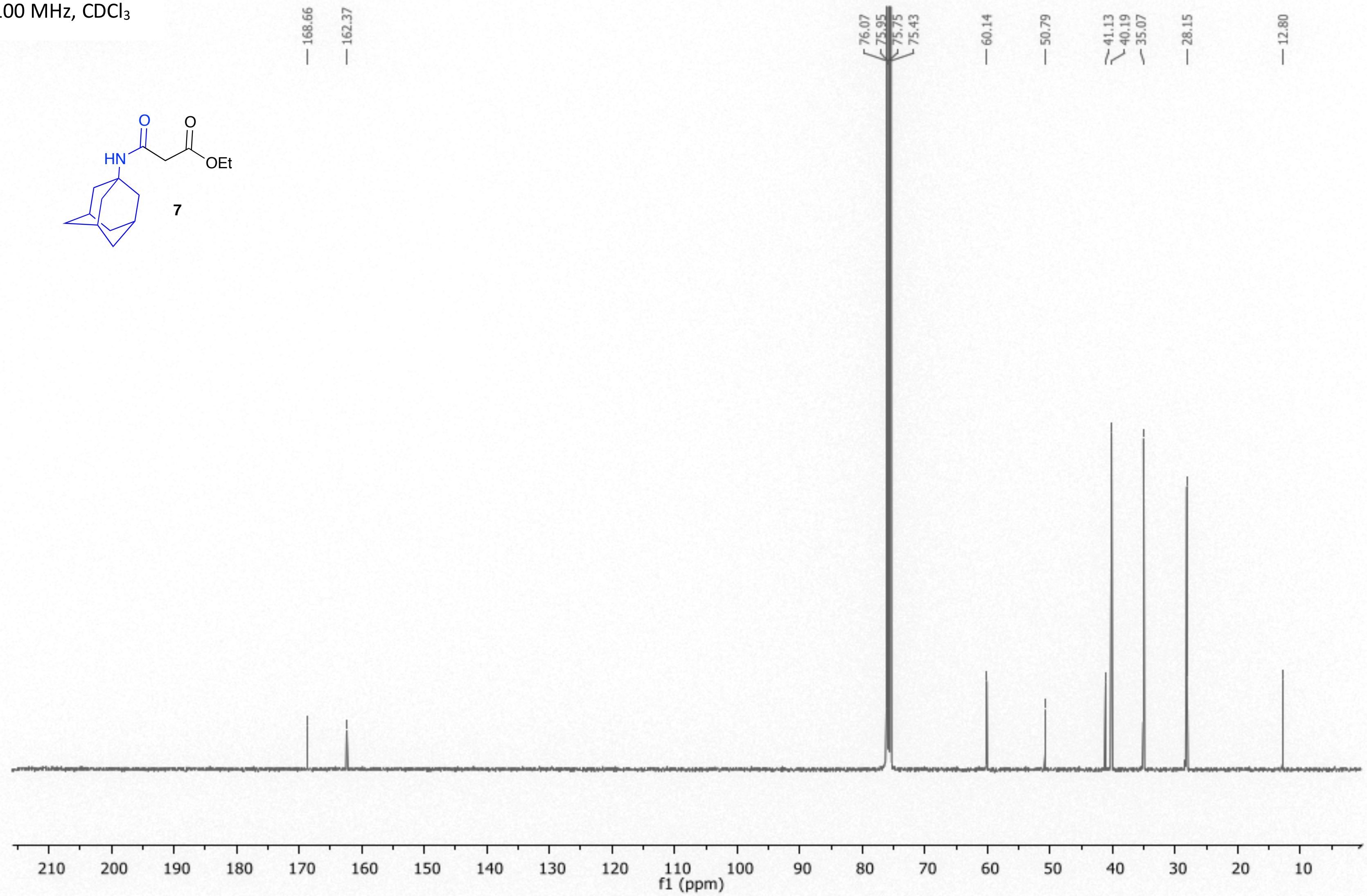

^{1}H NMR
400 MHz, $CDCl_3$

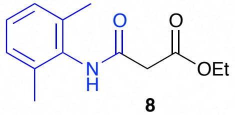

8

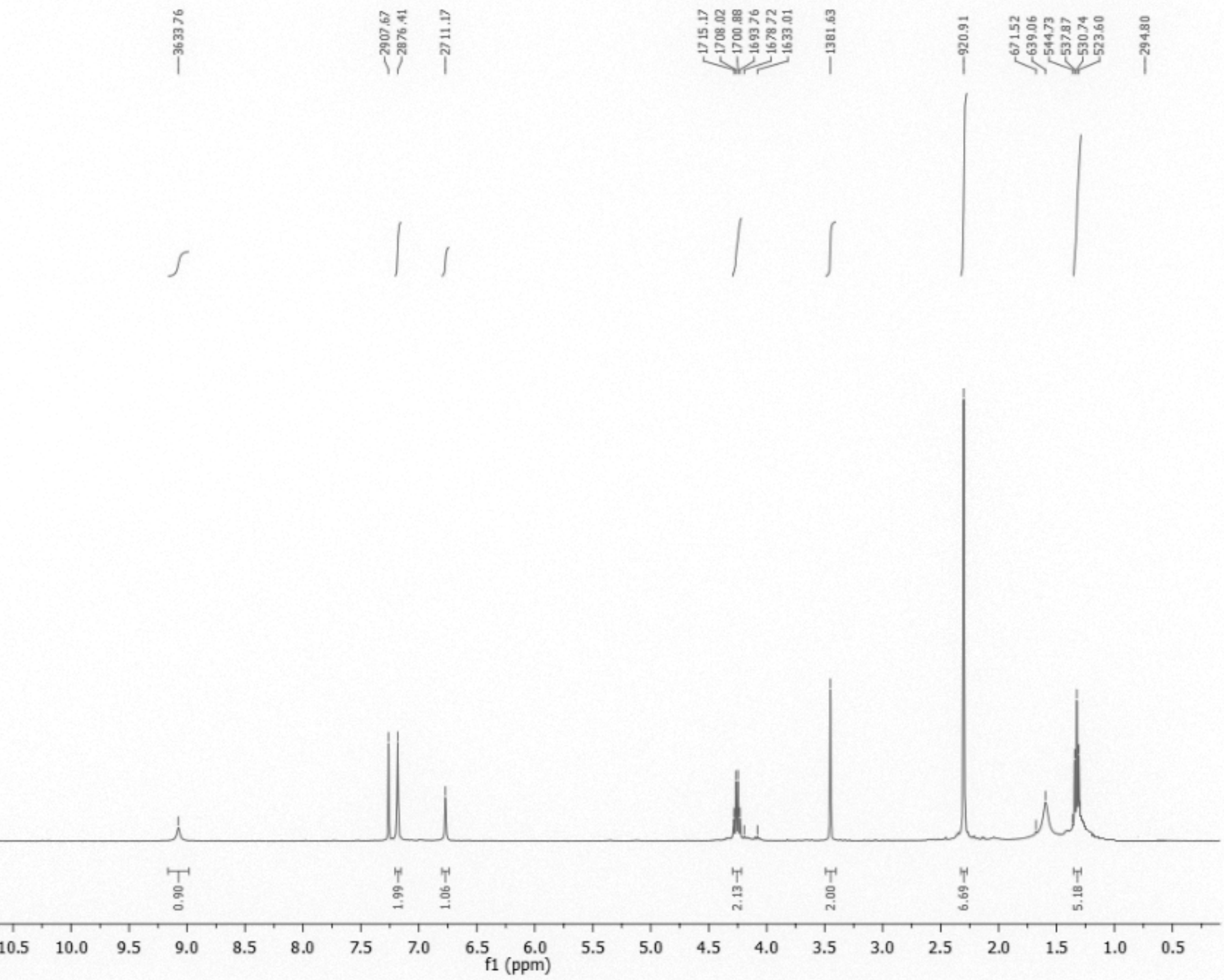

^{13}C NMR
100 MHz, $CDCl_3$

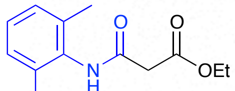

8

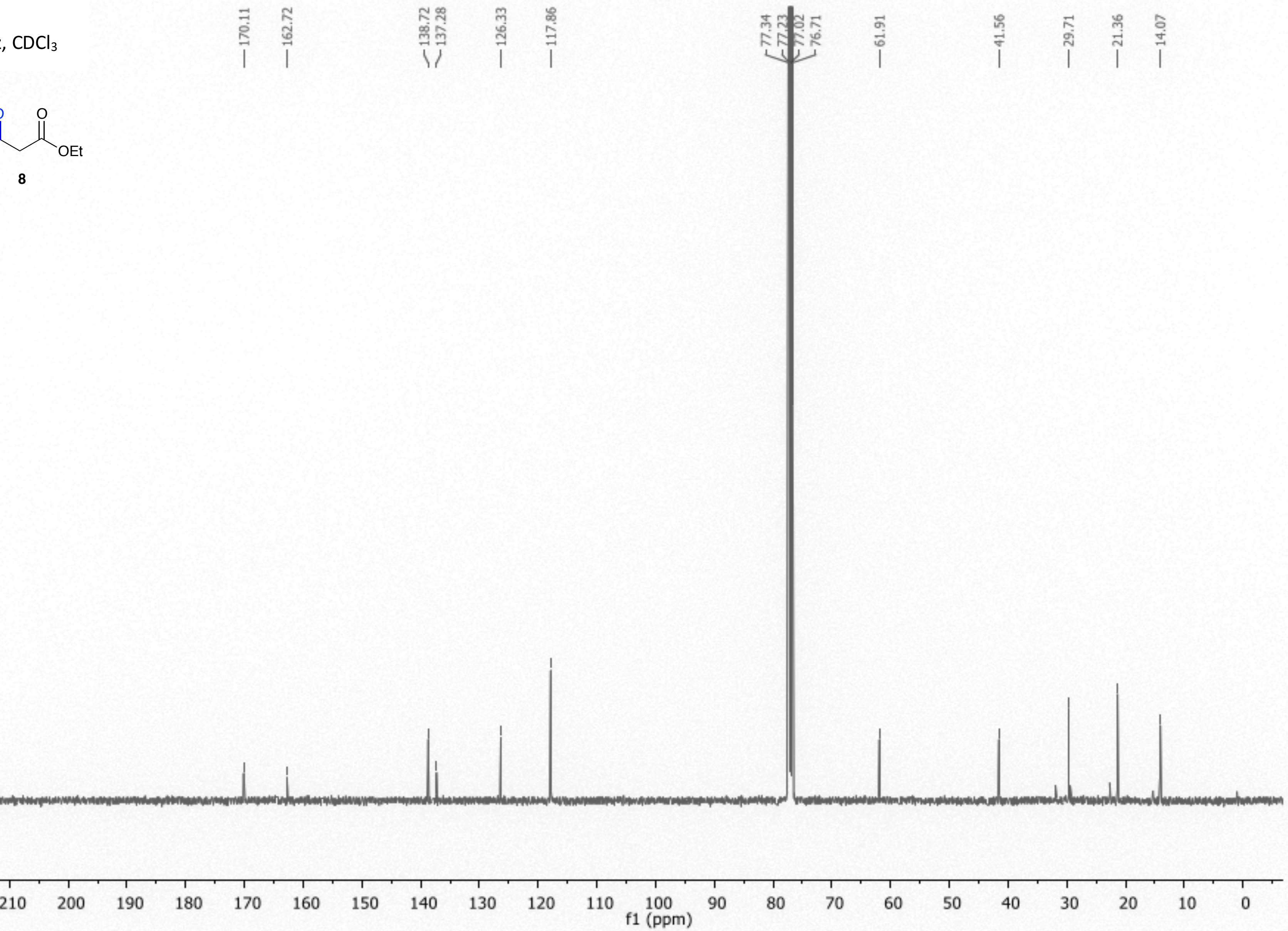

^{1}H NMR
400 MHz, $CDCl_3$

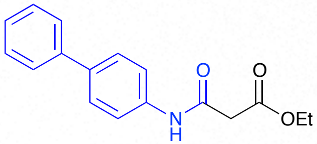

9

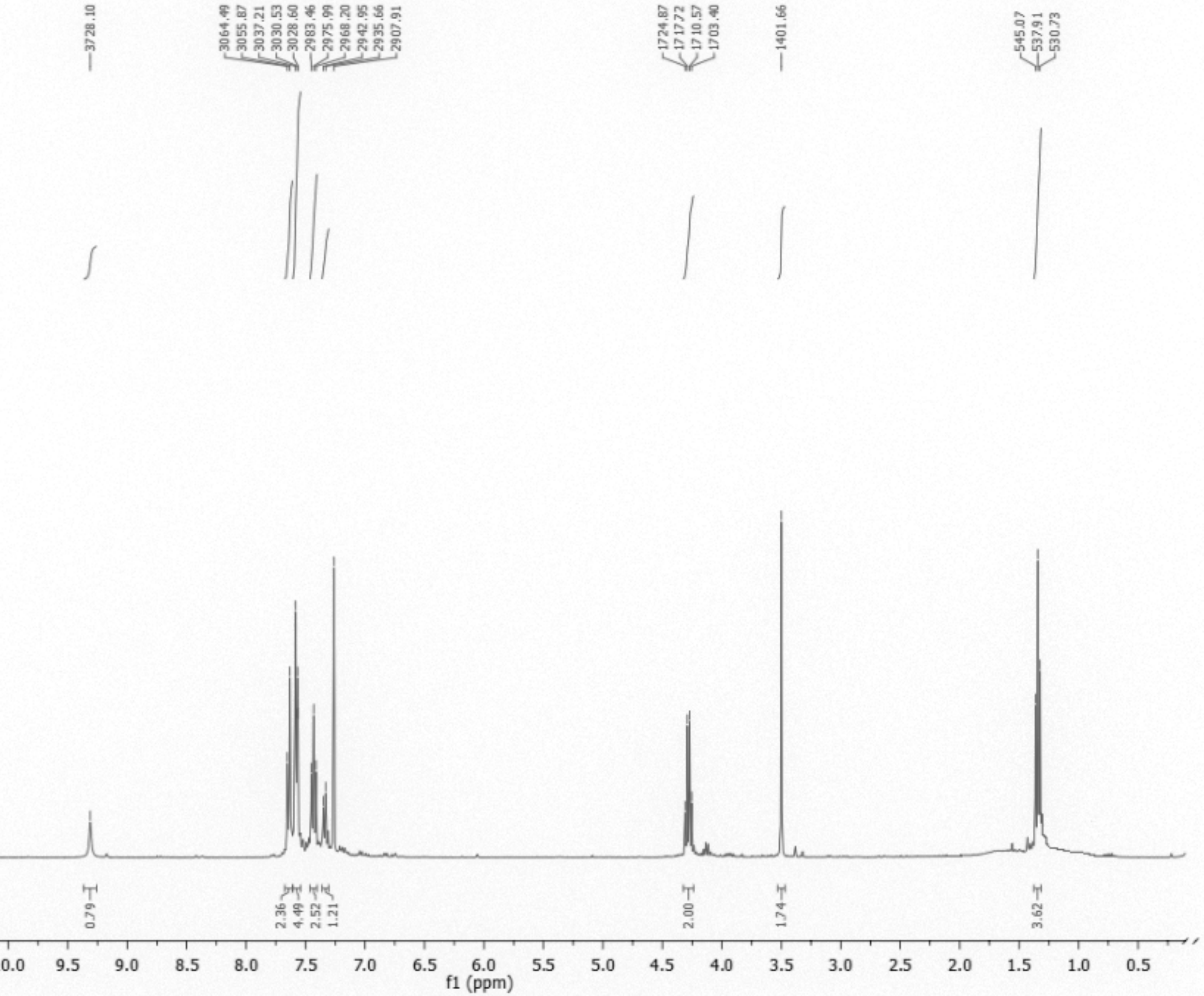

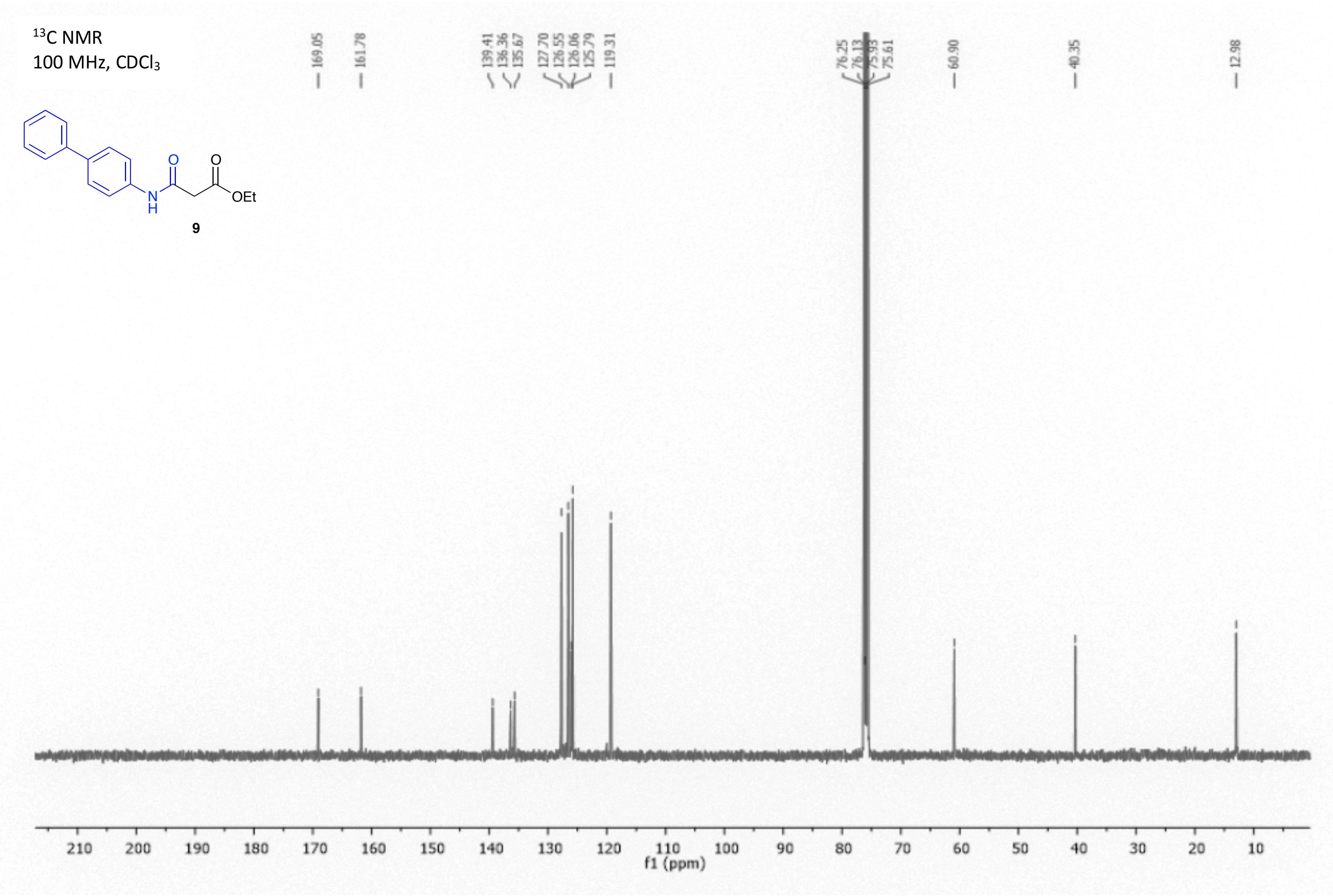
^{13}C NMR
100 MHz, CDCl$_3$
O
O
N
H
OEt
9
169.05
161.78
139.41
136.36
135.67
127.70
126.55
126.06
125.79
119.31
76.25
76.13
75.93
75.61
60.90
40.35
12.98
210
200
190
180
170
160
150
140
130
120
110
100
90
80
70
60
50
40
30
20
10
f1 (ppm)

^{1}H NMR
400 MHz, $CDCl_3$

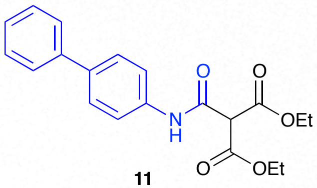

11

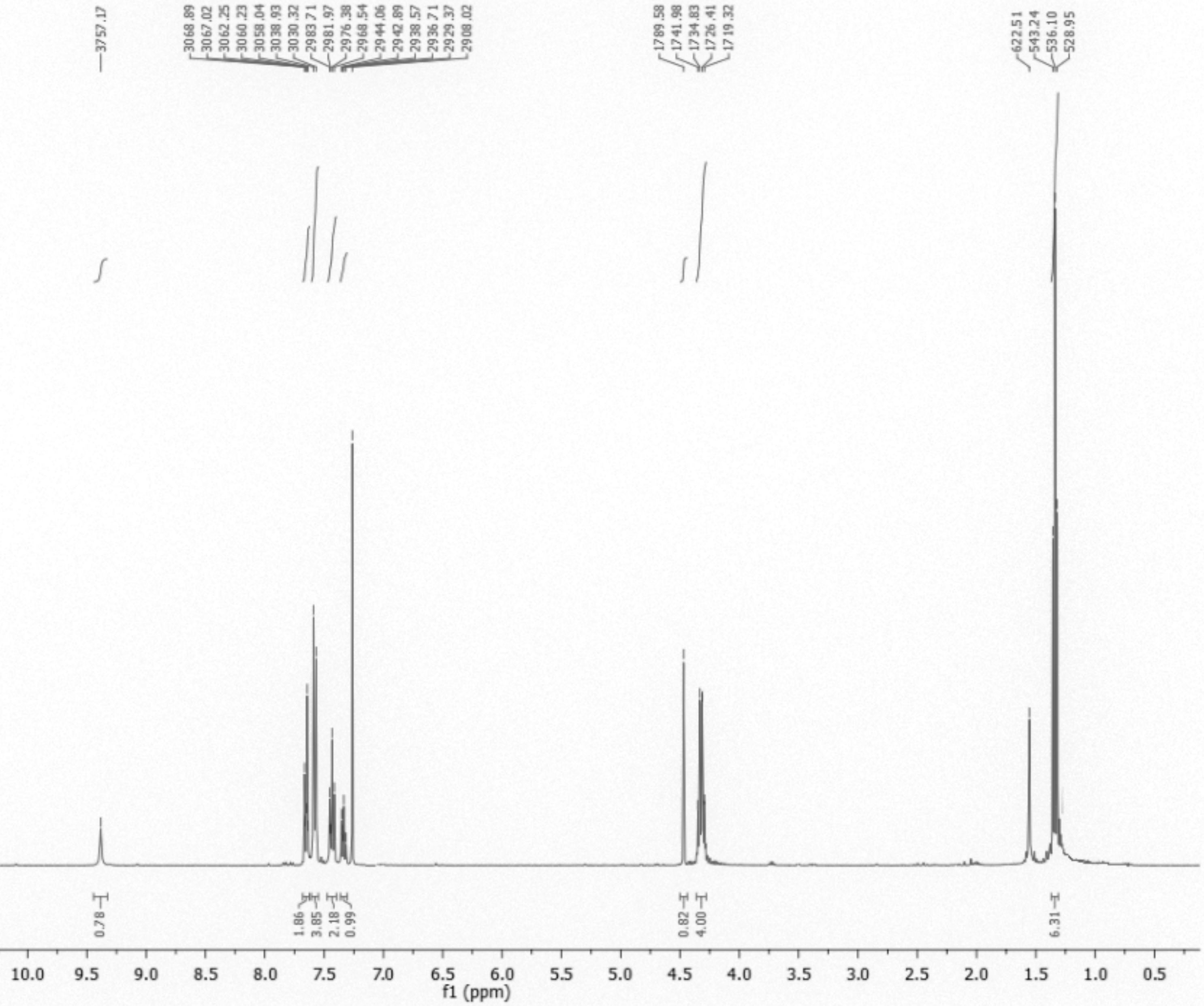

^{13}C NMR
100 MHz, $CDCl_3$

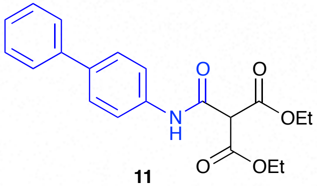

11

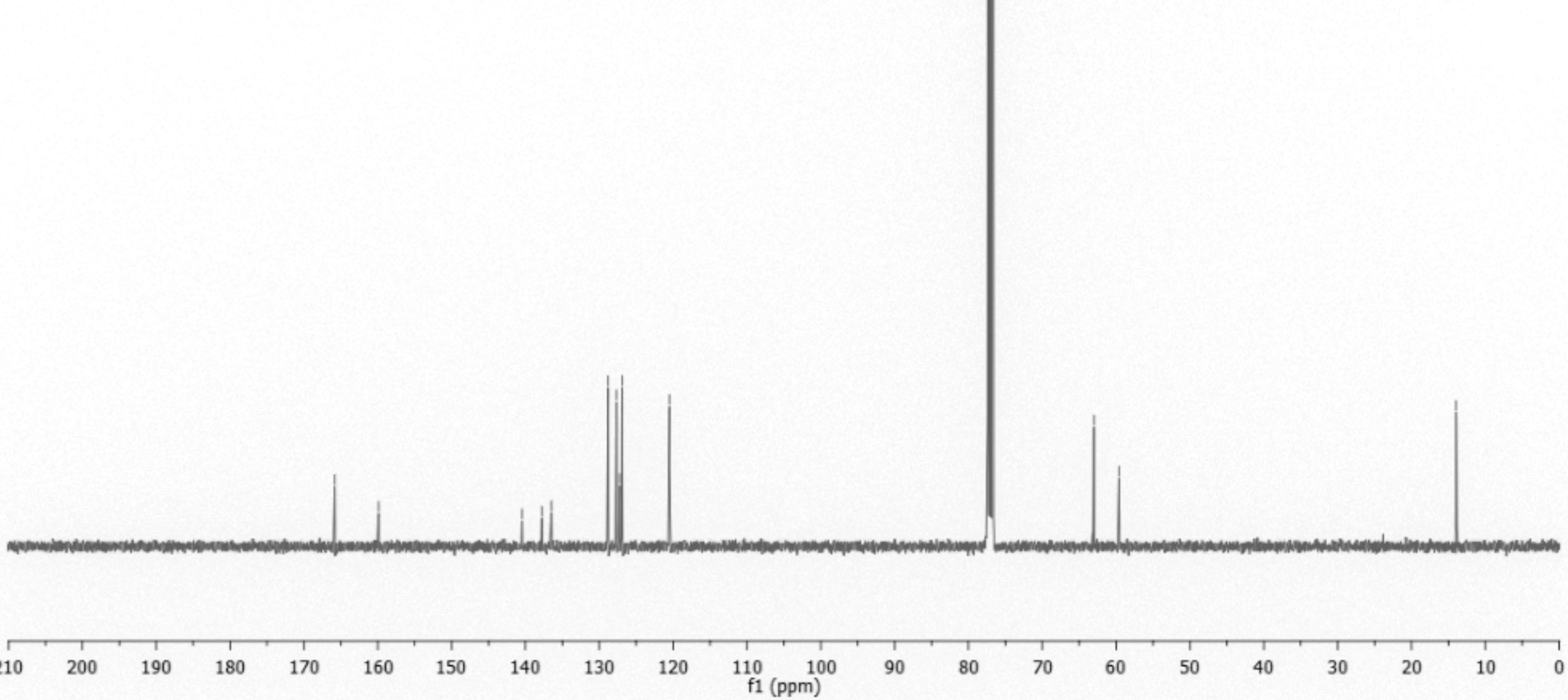

^1H NMR
400 MHz, $CDCl_3$

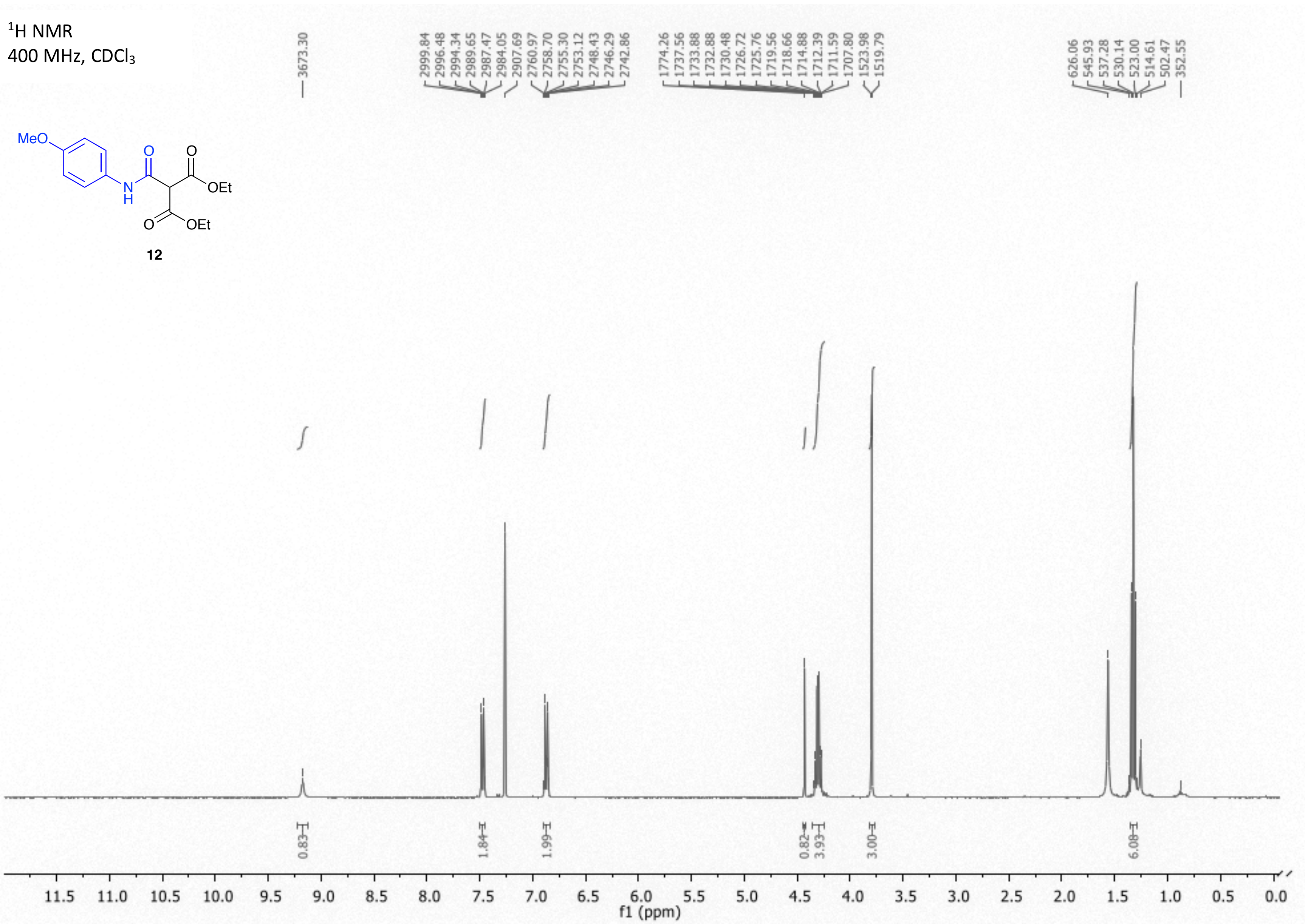

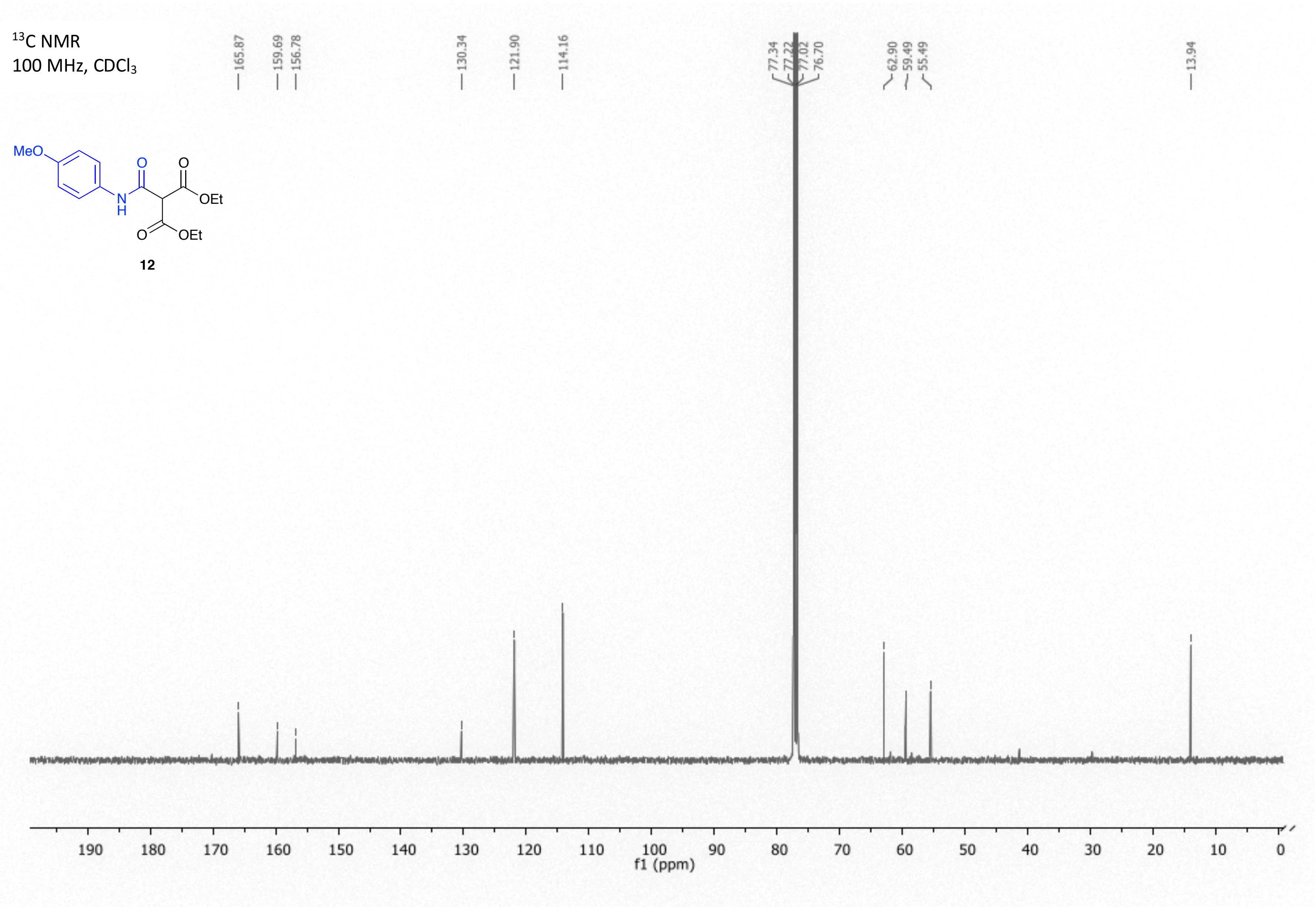
^{13}C NMR
100 MHz, $CDCl_3$
165.87
159.69
156.78
130.34
121.90
114.16
77.34
77.22
77.02
76.70
62.90
59.49
55.49
13.94
MeO
O
O
N
H
OEt
O
OEt
12
190 180 170 160 150 140 130 120 110 100 90 80 70 60 50 40 30 20 10 0
f1 (ppm)

^{1}H NMR
400 MHz, $CDCl_3$

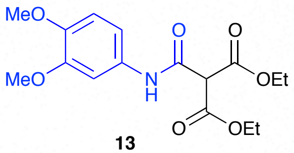

13

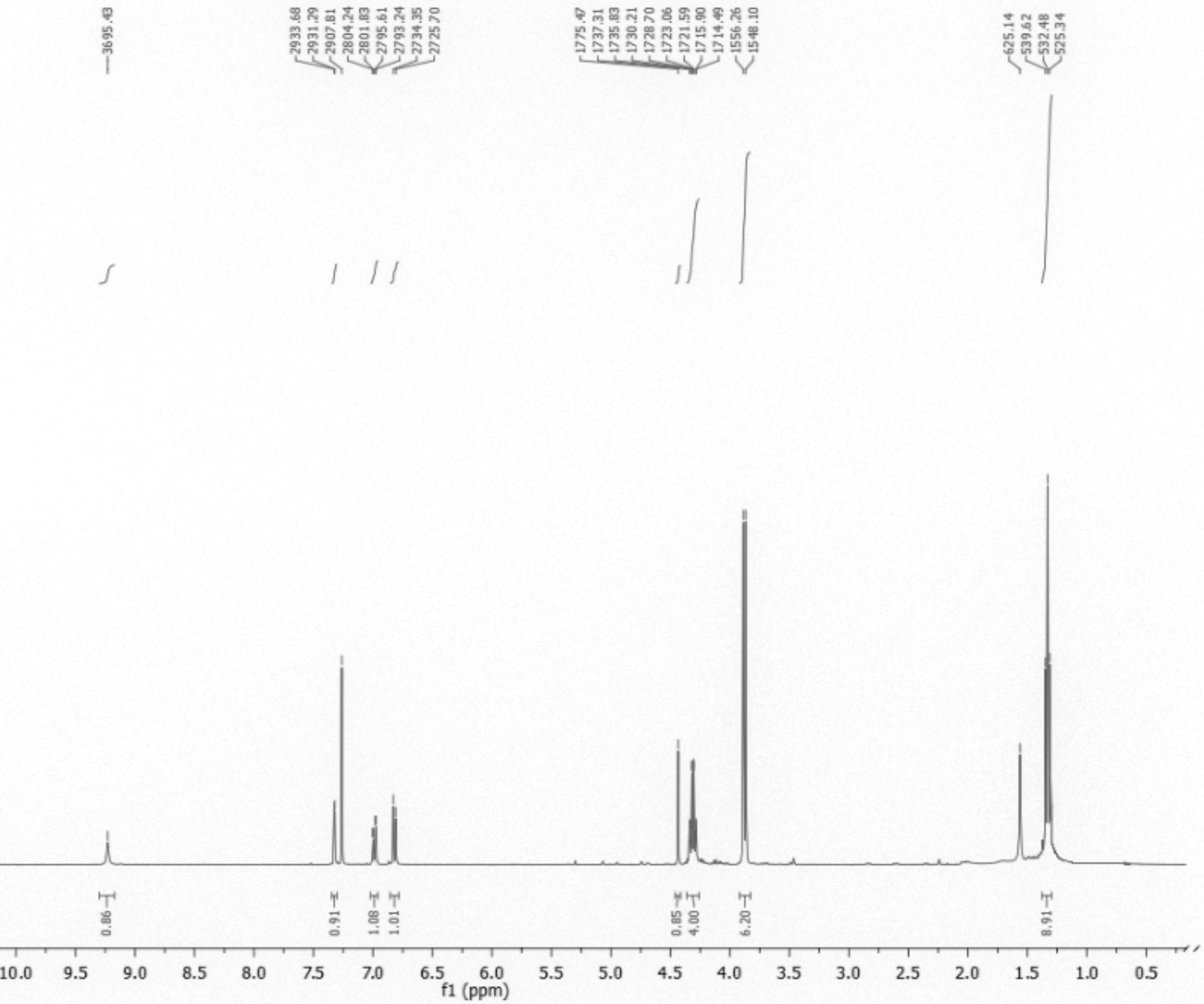

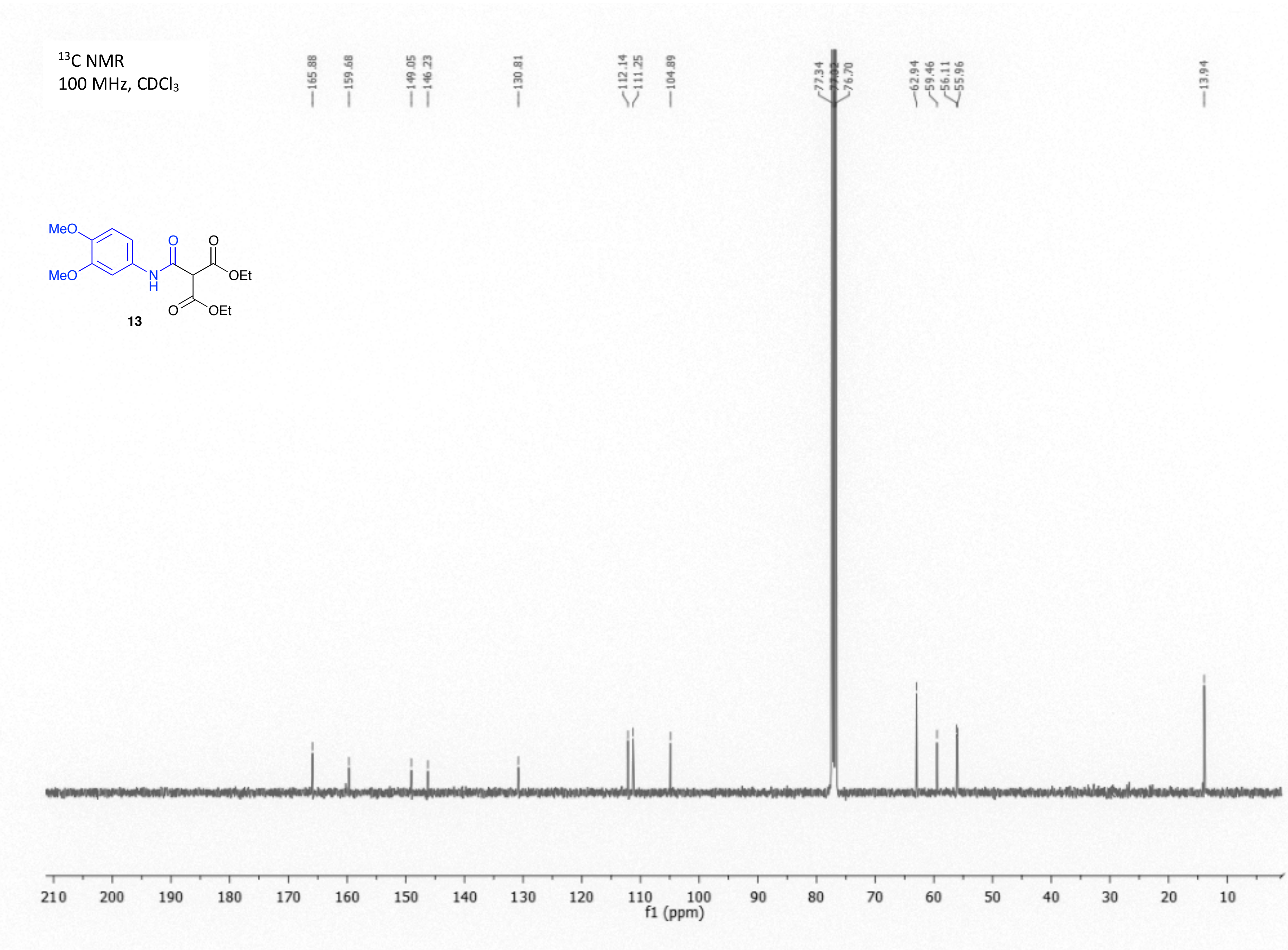

^{13}C NMR
100 MHz, CDCl$_3$
165.88
159.68
149.05
146.23
130.81
112.14
111.25
104.89
77.34
77.02
76.70
62.94
59.46
56.11
55.96
13.94
MeO
MeO
N
H
O
O
OEt
O
OEt
13
210
200
190
180
170
160
150
140
130
120
110
100
90
80
70
60
50
40
30
20
10
f1 (ppm)

^{1}H NMR
400 MHz, $CDCl_3$

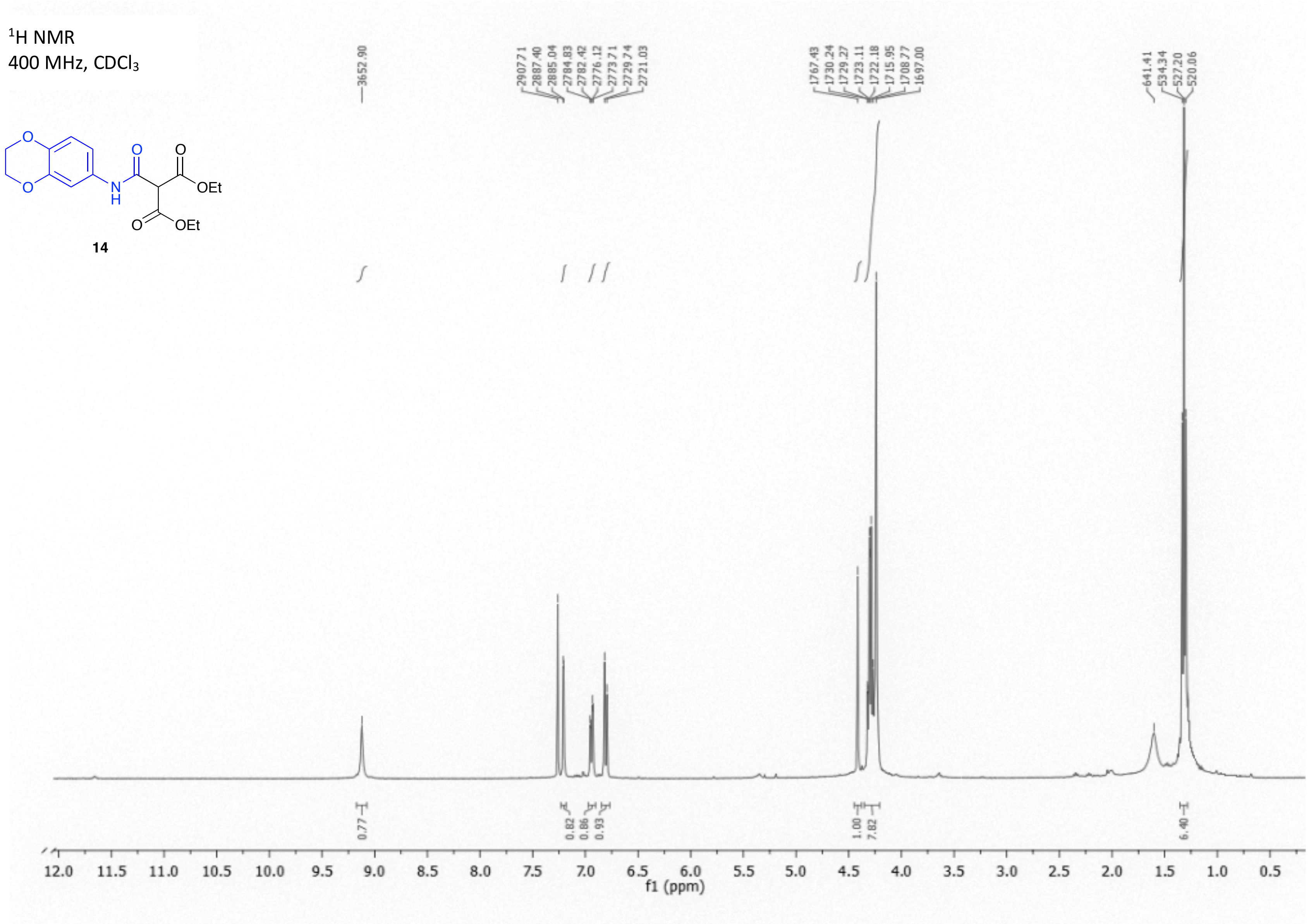

14

^{13}C NMR
100 MHz, $CDCl_3$

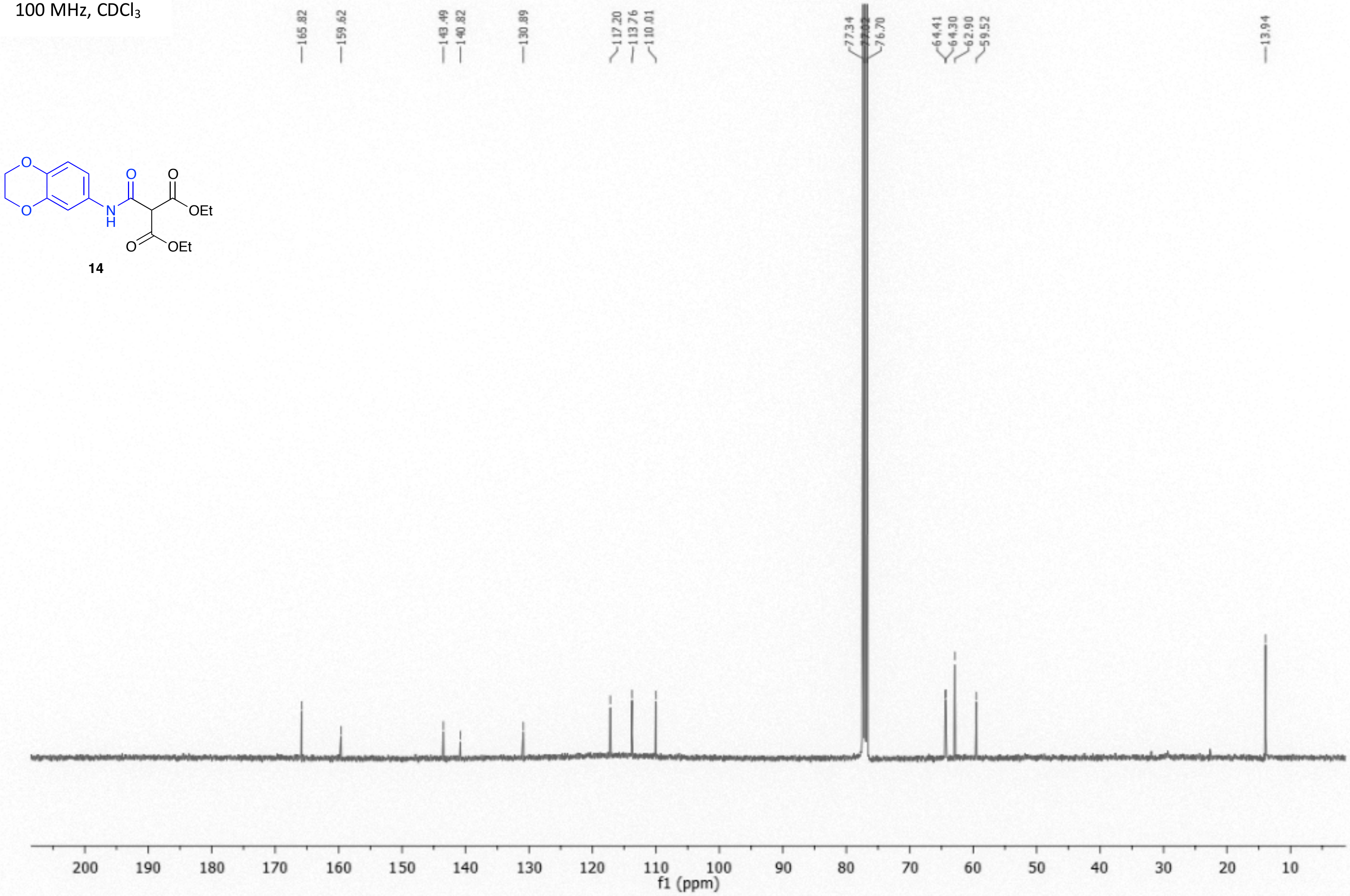

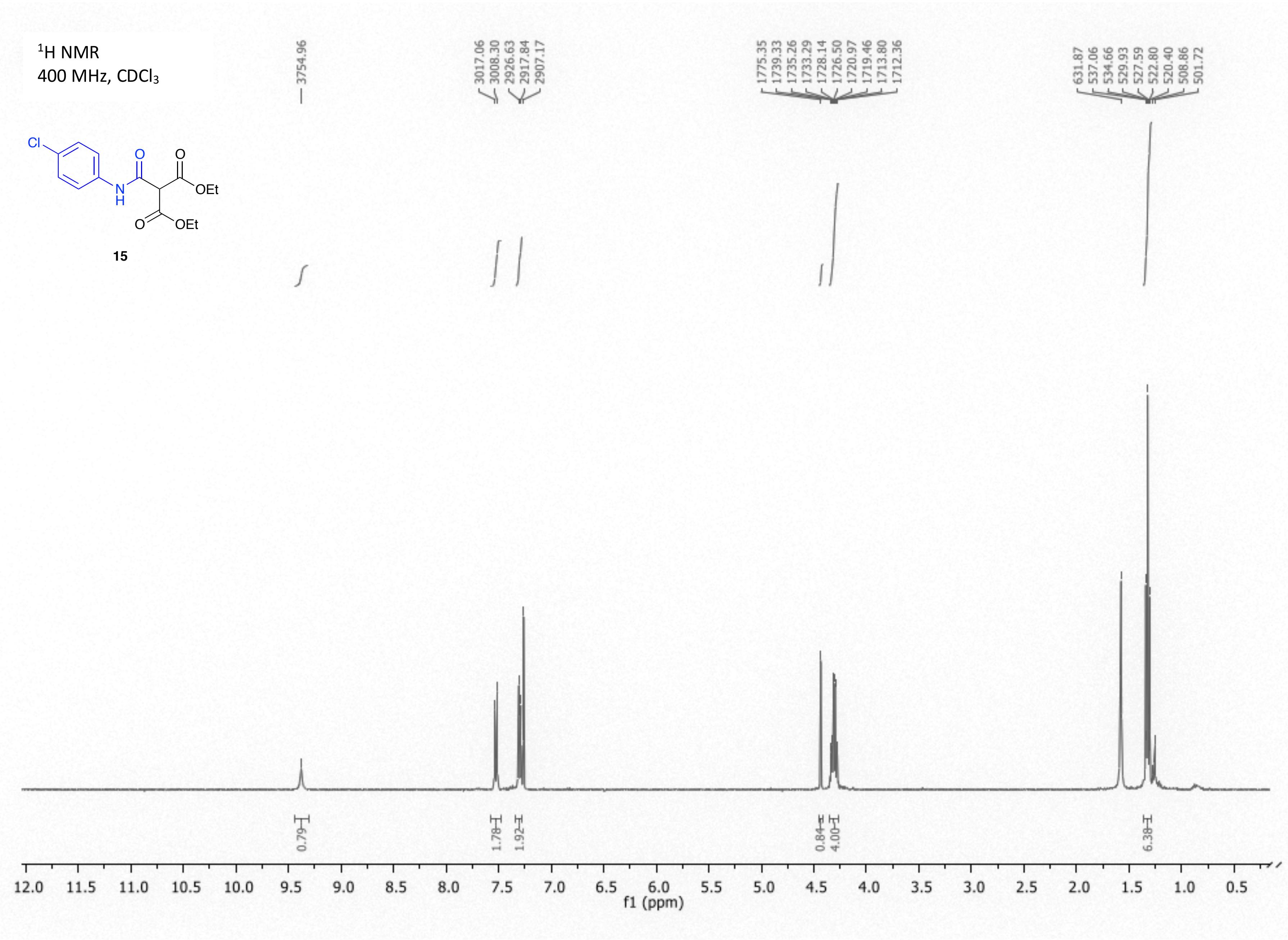

1H NMR
400 MHz, CDCl3
Cl
O
O
N
H
OEt
O
OEt
15
3754.96
3017.06
3008.30
2926.63
2917.84
2907.17
1775.35
1739.33
1735.26
1733.29
1728.14
1726.50
1720.97
1719.46
1713.80
1712.36
631.87
537.06
534.66
529.93
527.59
522.80
520.40
508.86
501.72
0.79
1.78
1.92
0.84
4.00
6.38
12.0 11.5 11.0 10.5 10.0 9.5 9.0 8.5 8.0 7.5 7.0 6.5 6.0 5.5 5.0 4.5 4.0 3.5 3.0 2.5 2.0 1.5 1.0 0.5
f1 (ppm)

^{13}C NMR
100 MHz, $CDCl_3$

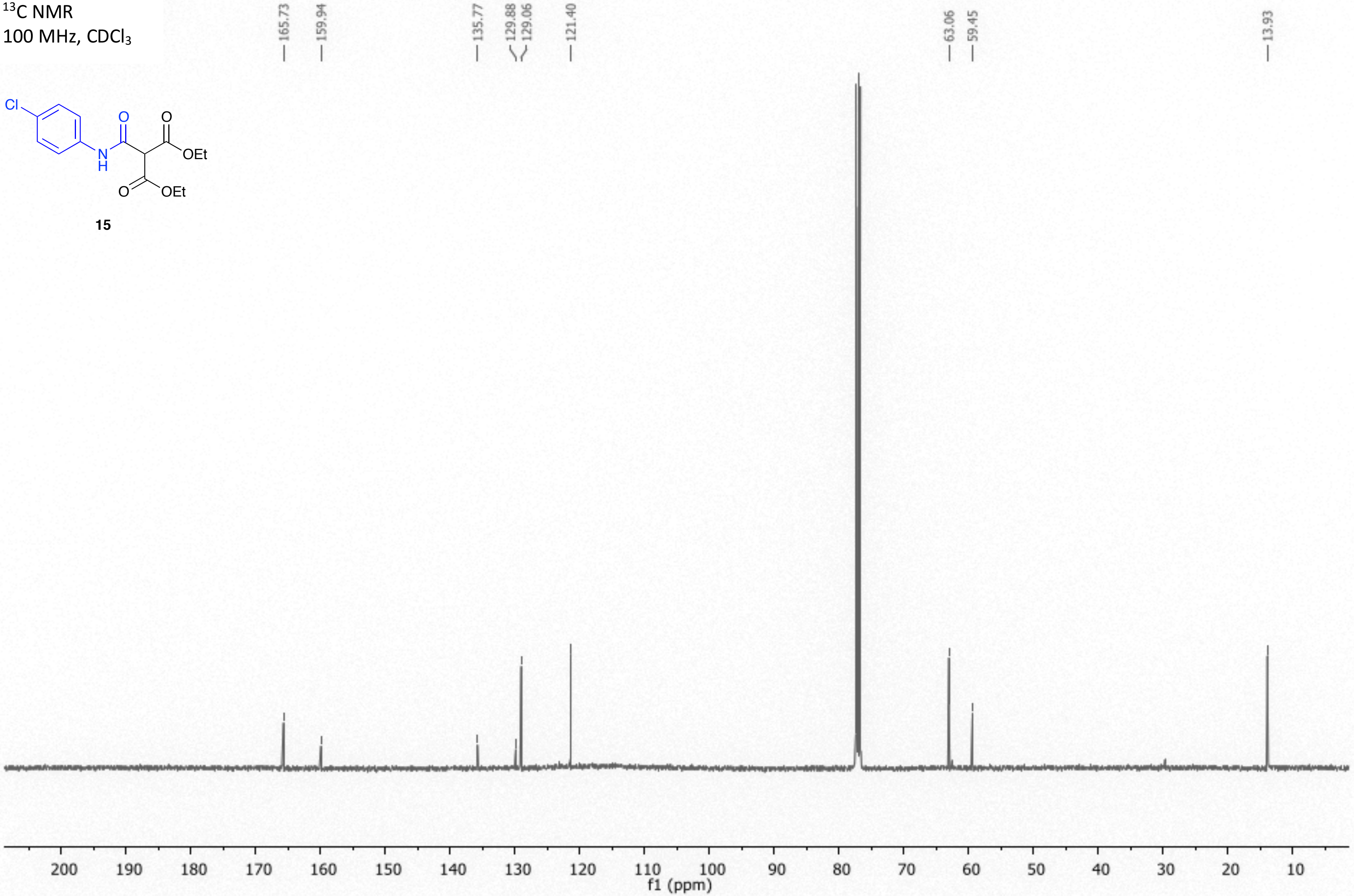

^{1}H NMR
400 MHz, $CDCl_3$

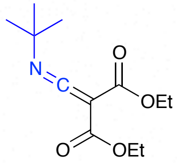

16

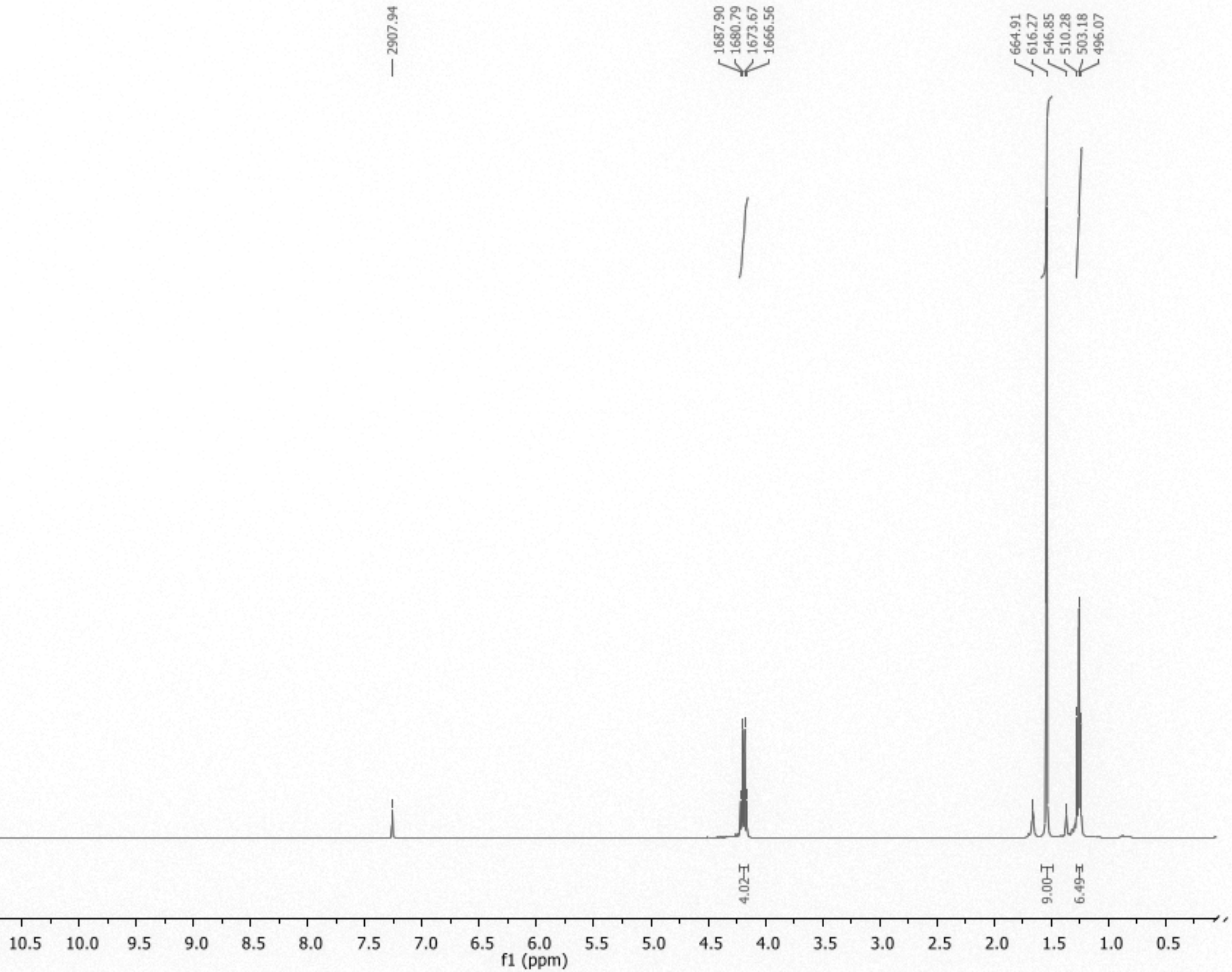

^{13}C NMR
100 MHz, $CDCl_3$

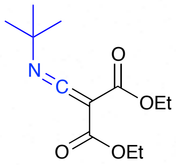

16

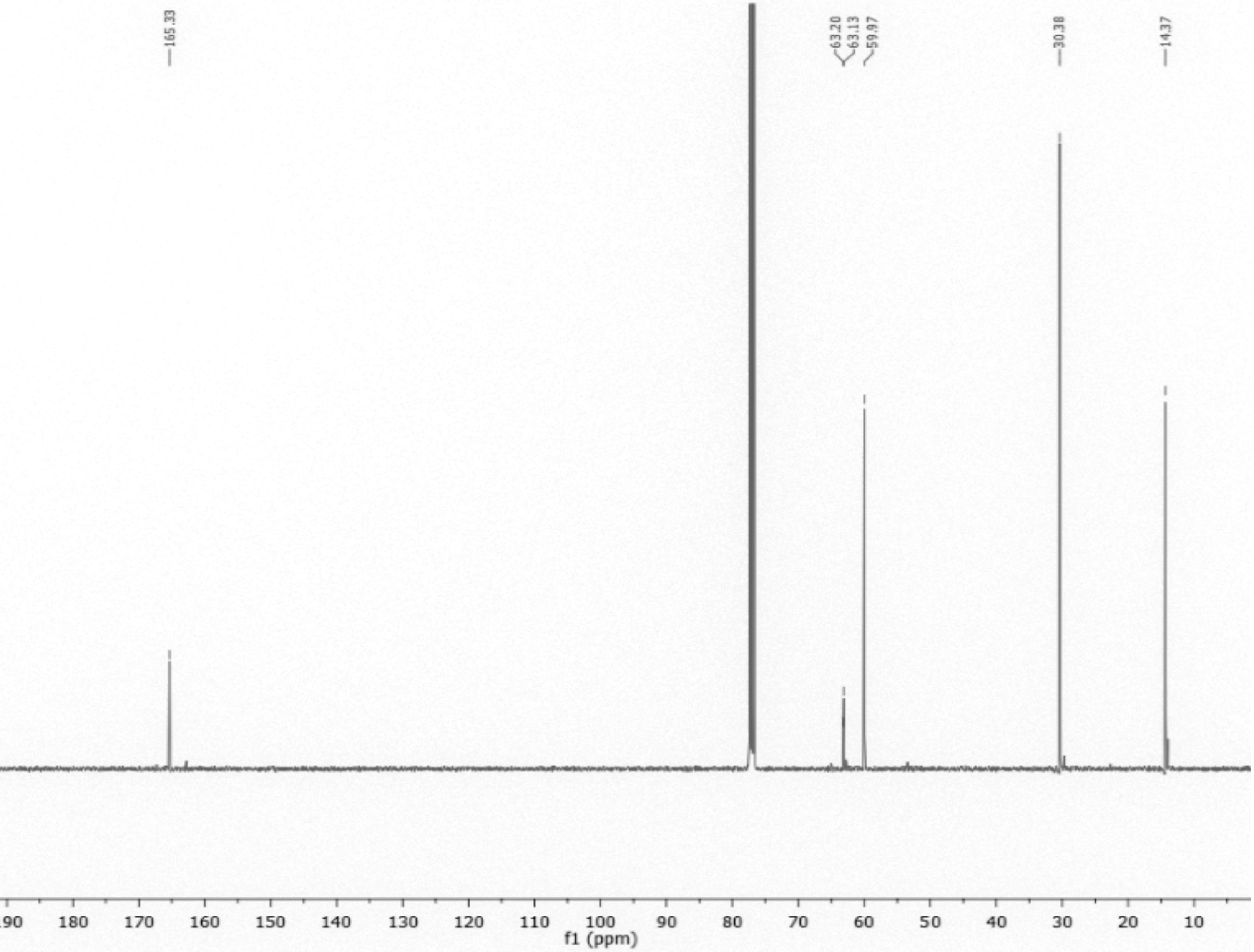

^{1}H NMR
400 MHz, $CDCl_3$

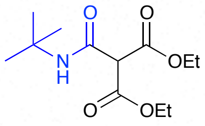

17

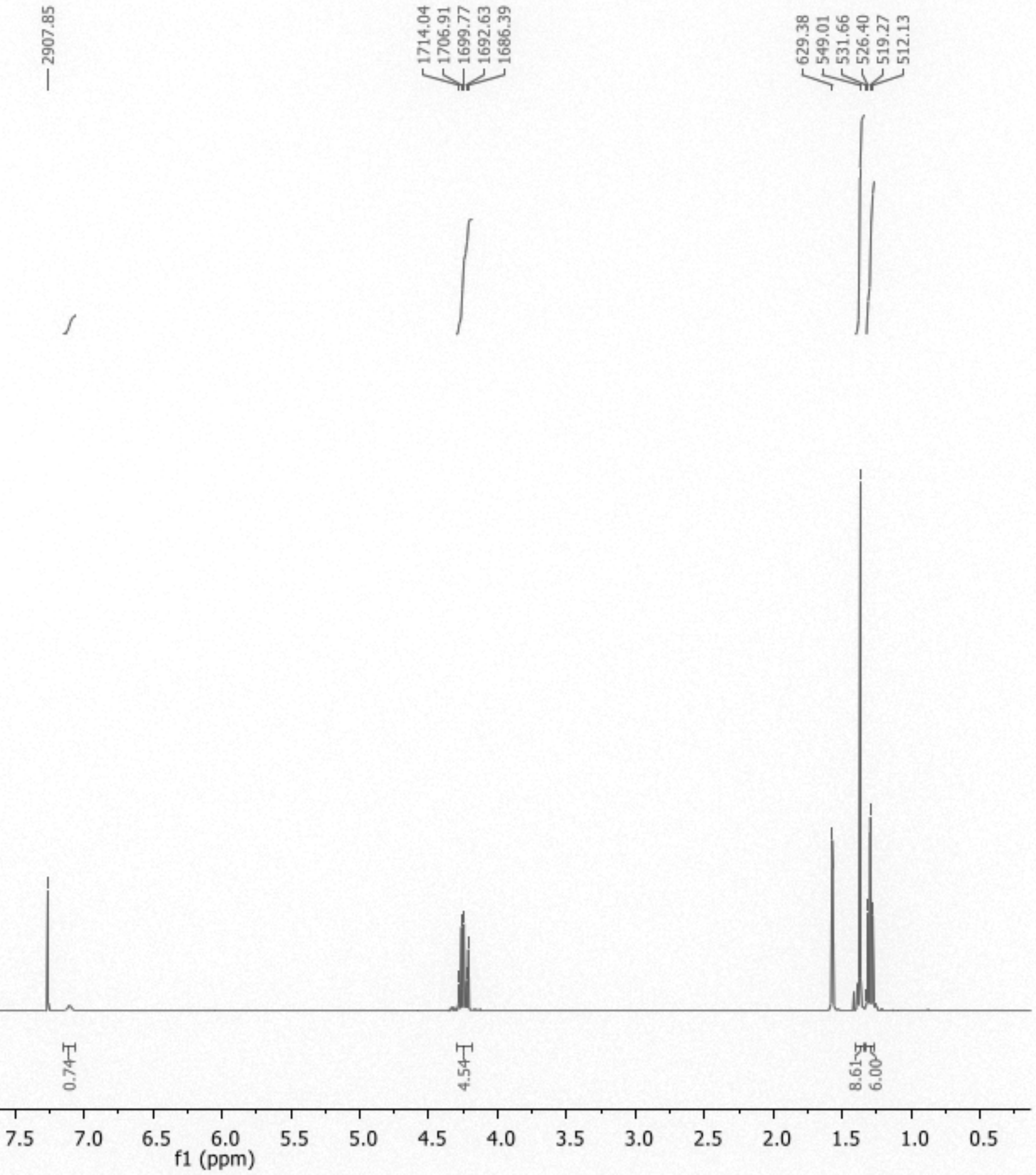

^{13}C NMR
100 MHz, $CDCl_3$

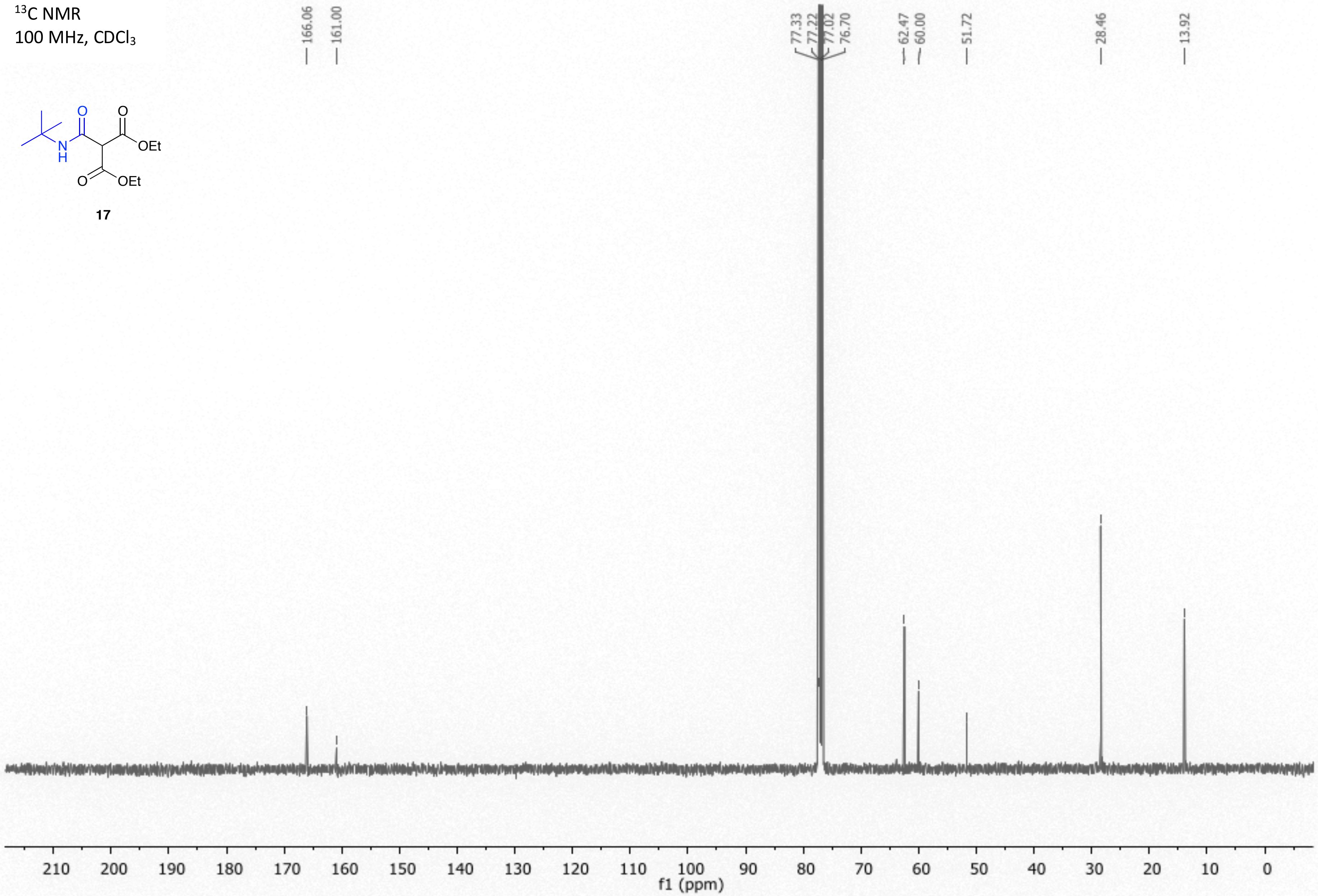

17

^1H NMR
400 MHz, $CDCl_3$

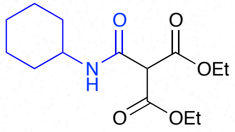

18

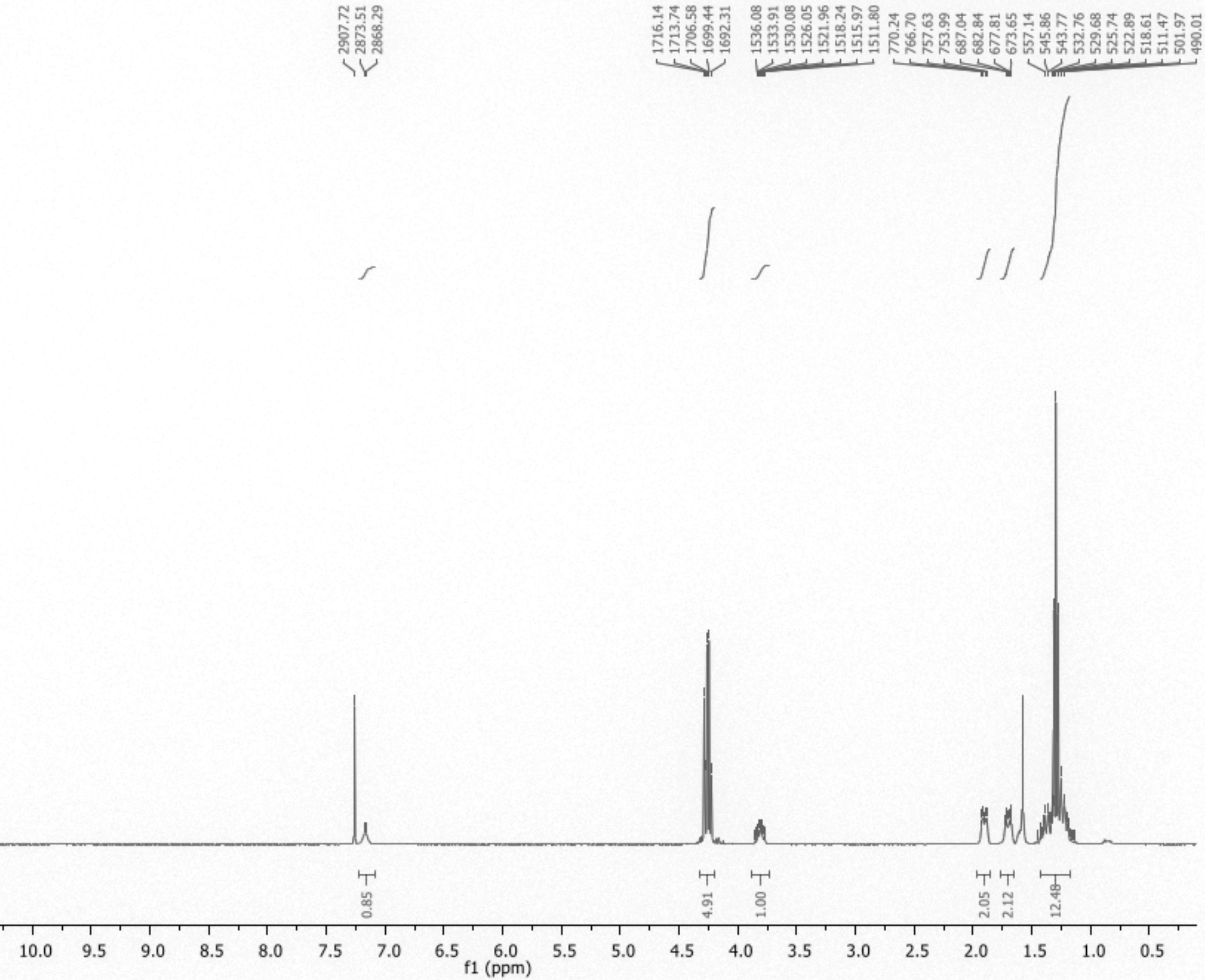

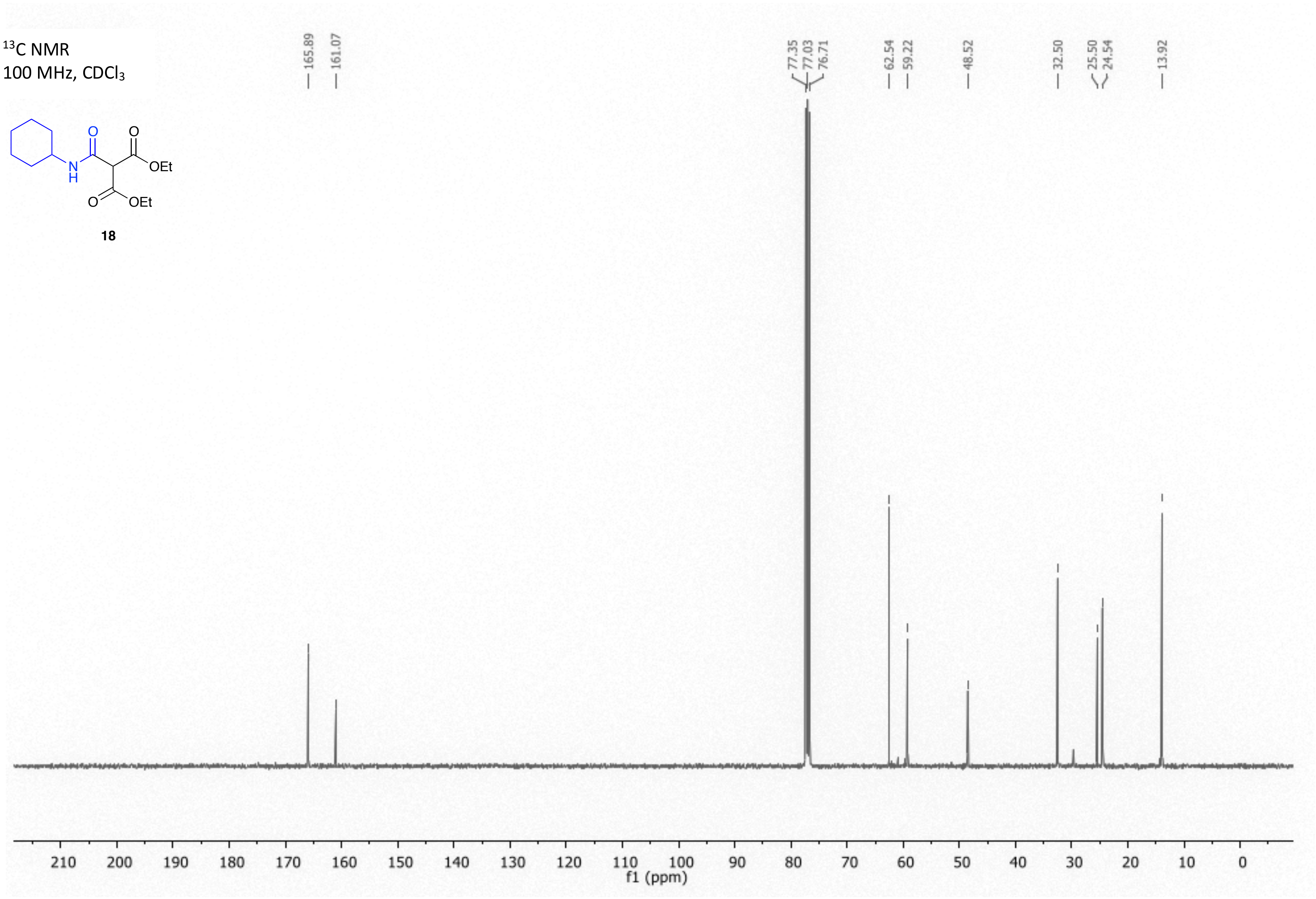
^{13}C NMR
100 MHz, $CDCl_3$
O
O
N
H
OEt
O
OEt
18
165.89
161.07
77.35
77.03
76.71
62.54
59.22
48.52
32.50
25.50
24.54
13.92
210 200 190 180 170 160 150 140 130 120 110 100 90 80 70 60 50 40 30 20 10 0
f1 (ppm)

^{1}H NMR
400 MHz, $CDCl_3$

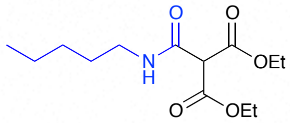

19

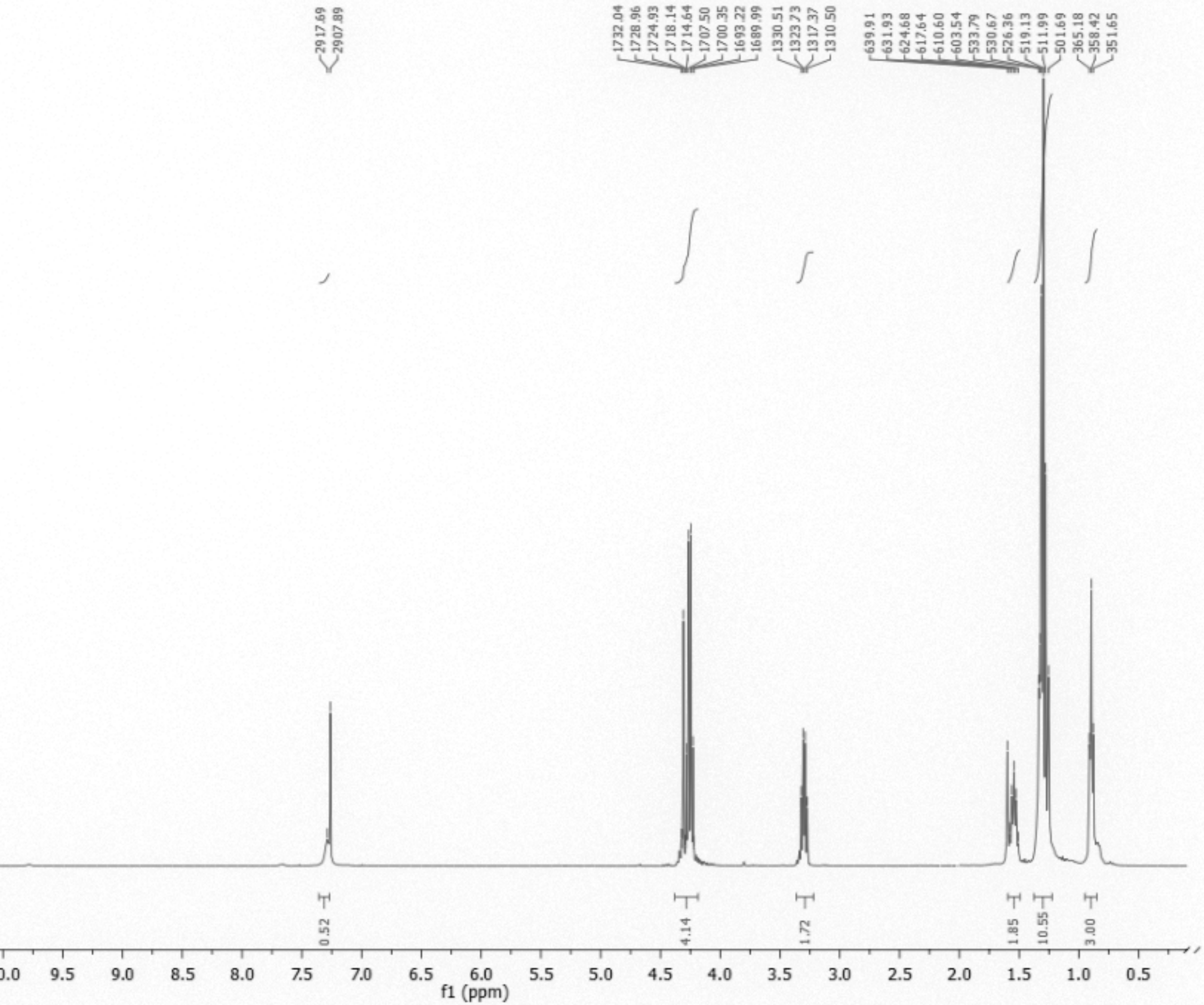

^{13}C NMR
100 MHz, $CDCl_3$

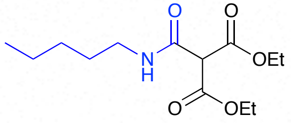

19

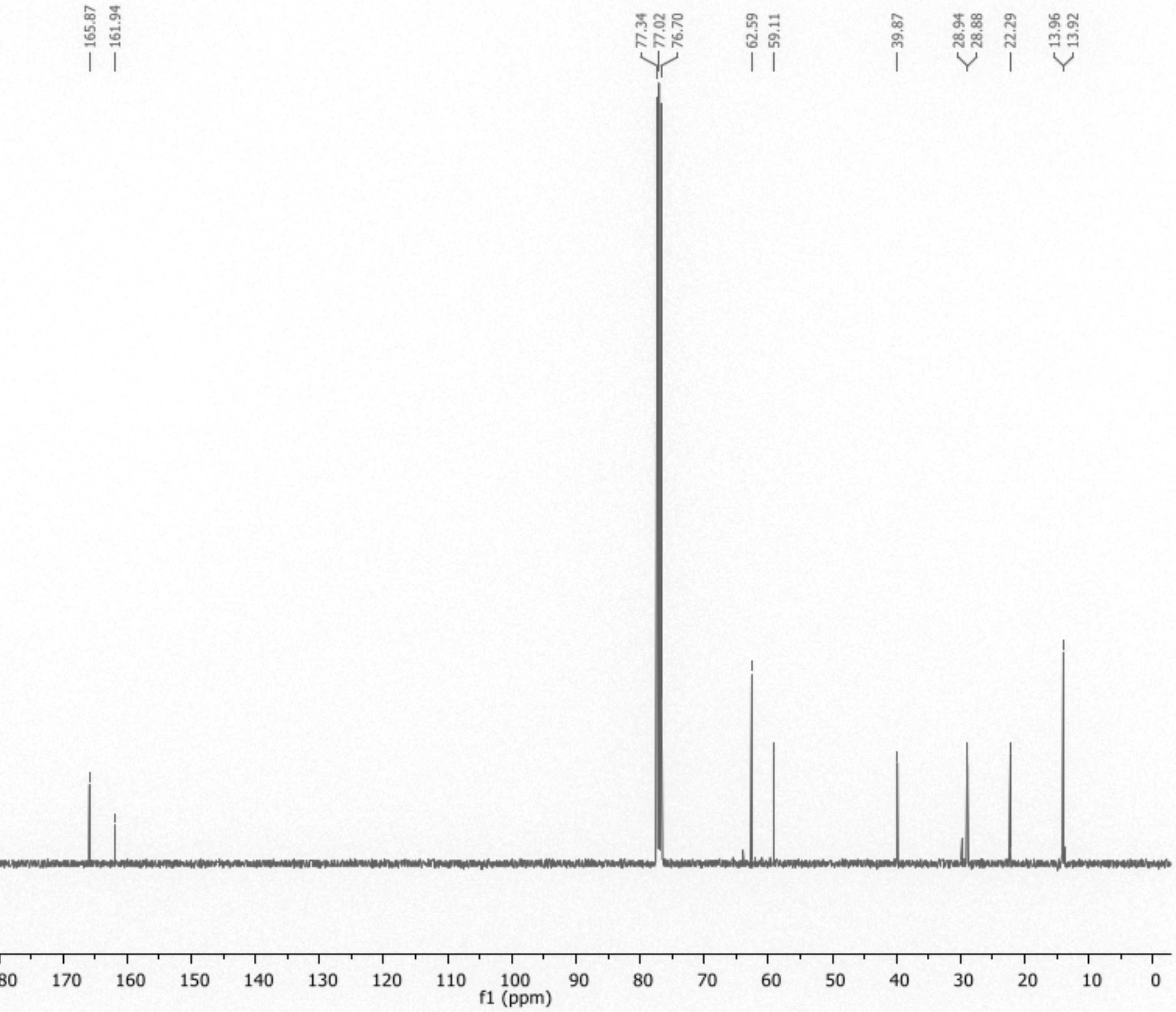

^{1}H NMR
400 MHz, $CDCl_3$

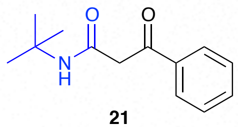

21

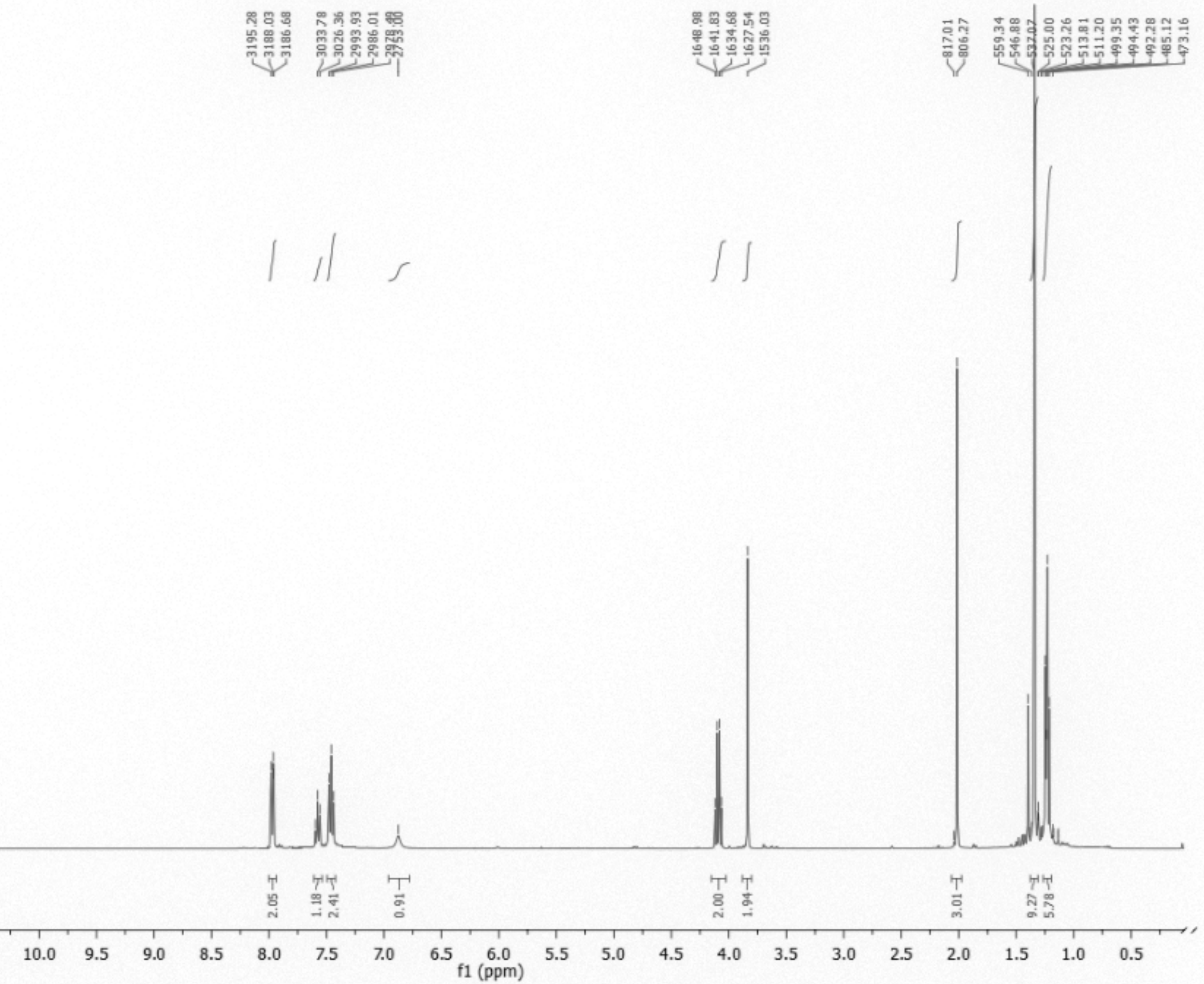

^{13}C NMR
100 MHz, $CDCl_3$

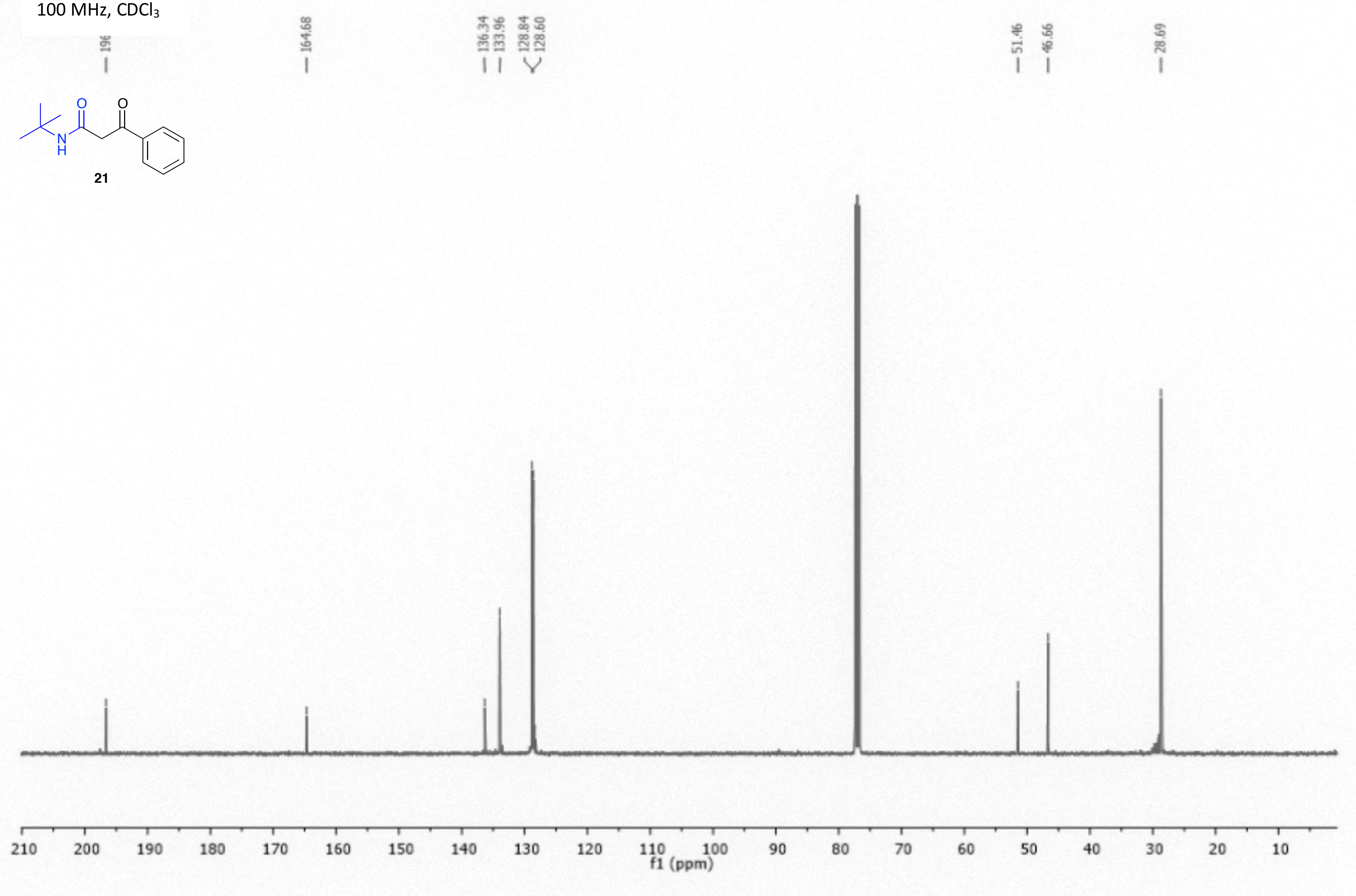

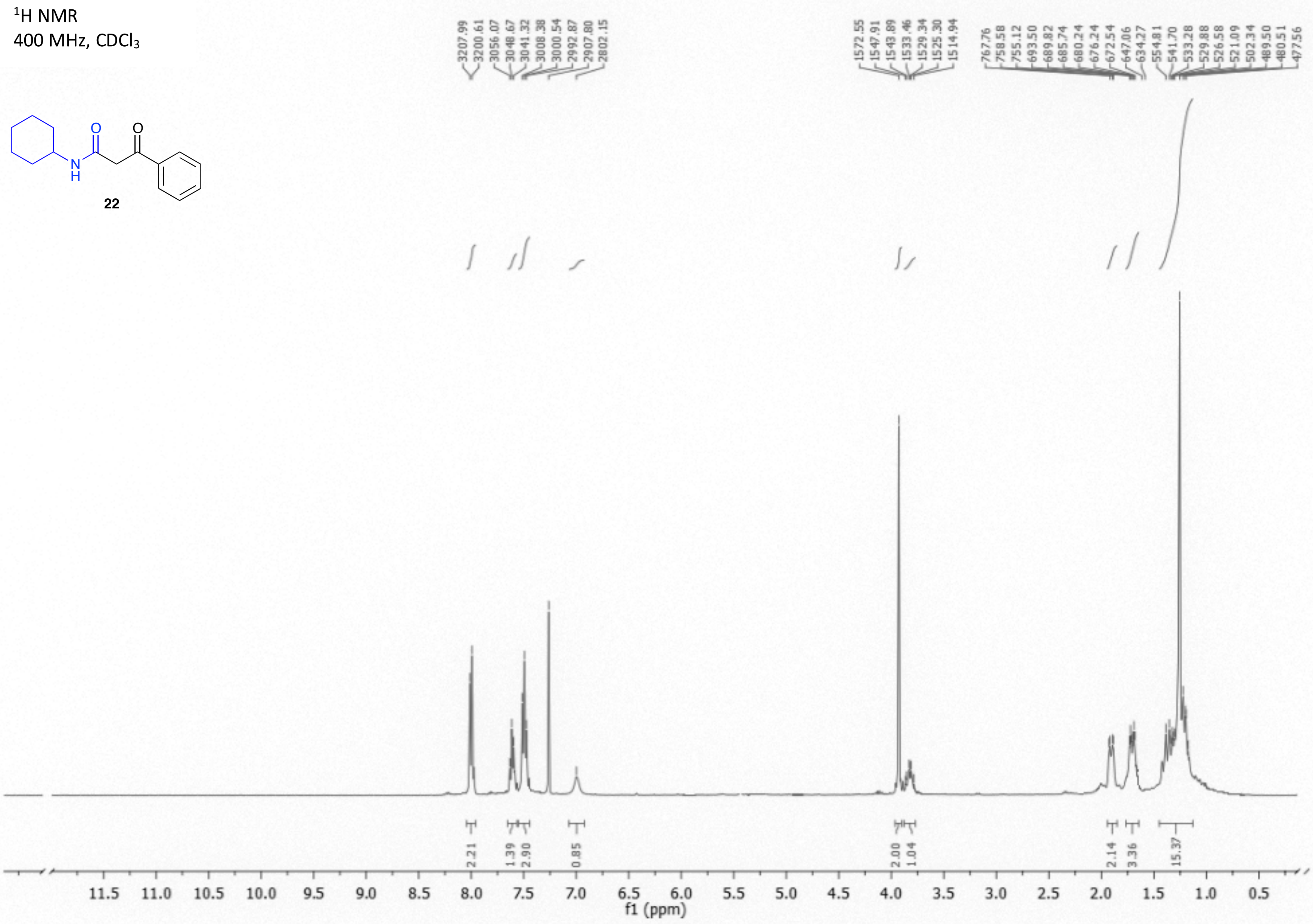
^{1}H NMR
400 MHz, $CDCl_3$
22
3207.99
3200.61
3056.07
3048.67
3041.32
3008.38
3000.54
2992.87
2907.80
2802.15
1572.55
1547.91
1543.89
1533.46
1529.34
1525.30
1514.94
767.76
758.58
755.12
693.50
689.82
685.74
680.24
676.24
672.54
647.06
634.27
554.81
541.70
533.28
529.88
526.58
521.09
502.34
489.50
480.51
477.56
2.21
1.39
2.90
0.85
2.00
1.04
2.14
3.36
15.37
11.5
11.0
10.5
10.0
9.5
9.0
8.5
8.0
7.5
7.0
6.5
6.0
5.5
5.0
4.5
4.0
3.5
3.0
2.5
2.0
1.5
1.0
0.5
f1 (ppm)

^{13}C NMR
100 MHz, $CDCl_3$

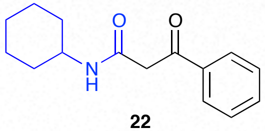

22

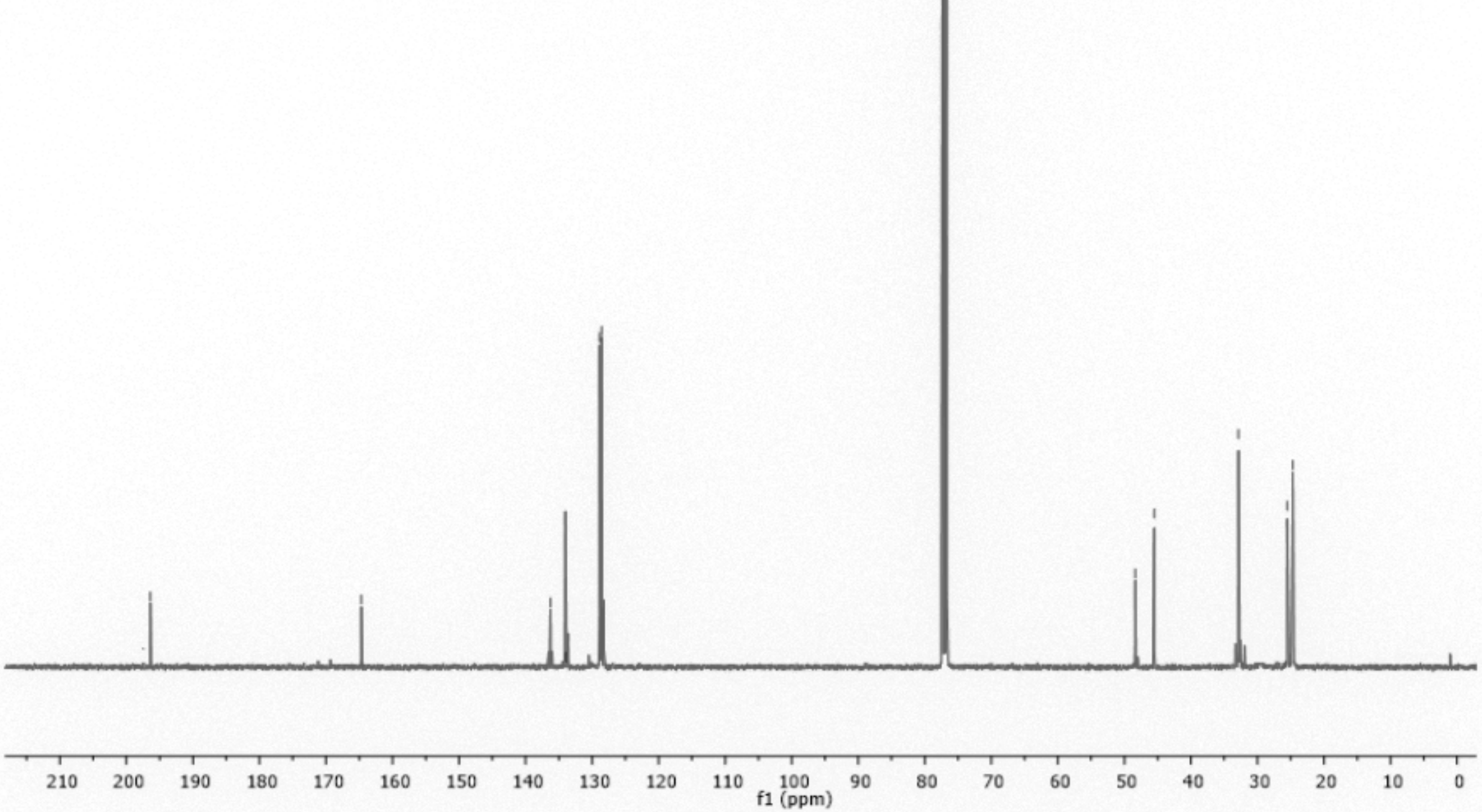

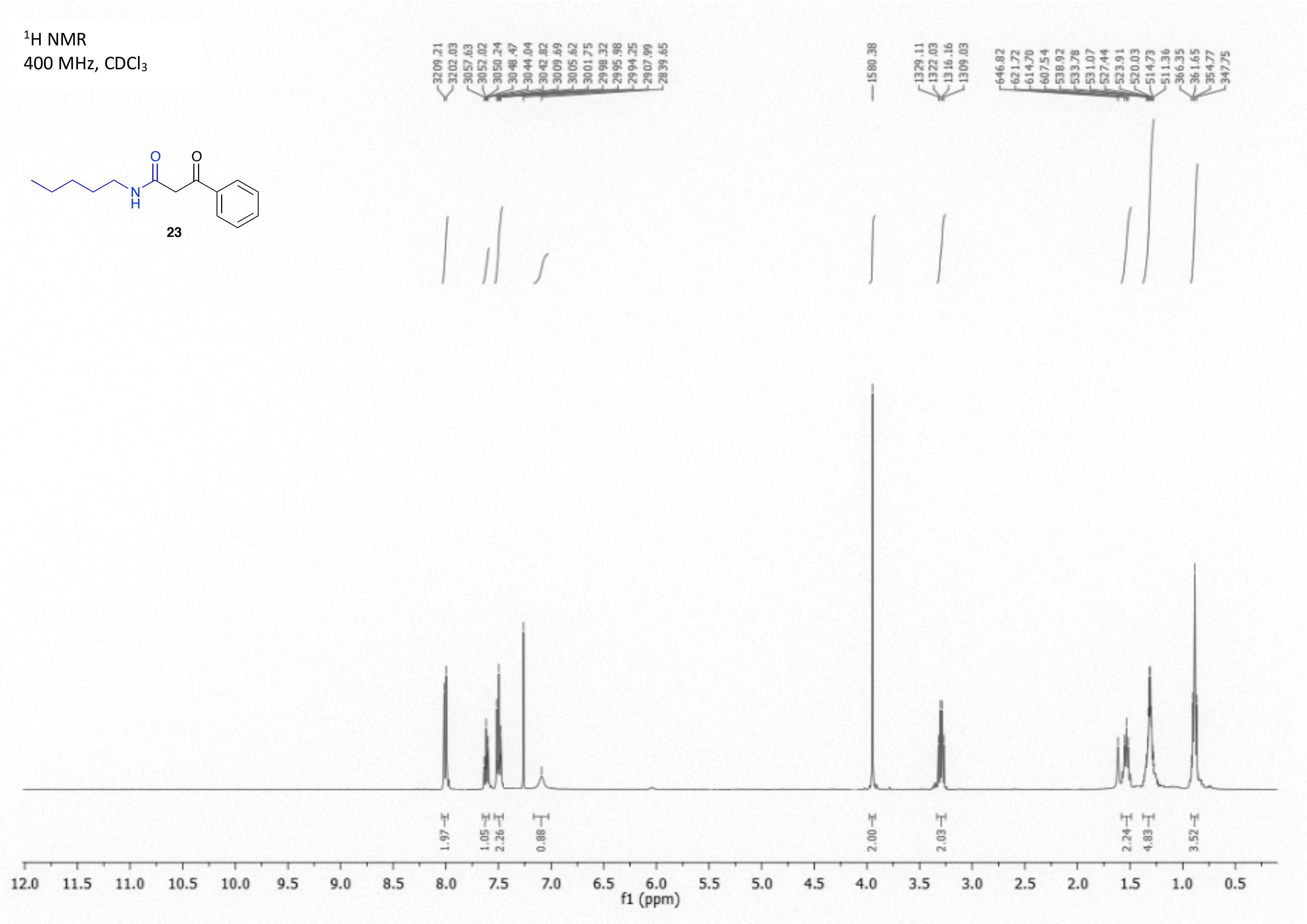

^1H NMR
400 MHz, $CDCl_3$
23
f1 (ppm)

^{13}C NMR
100 MHz, $CDCl_3$

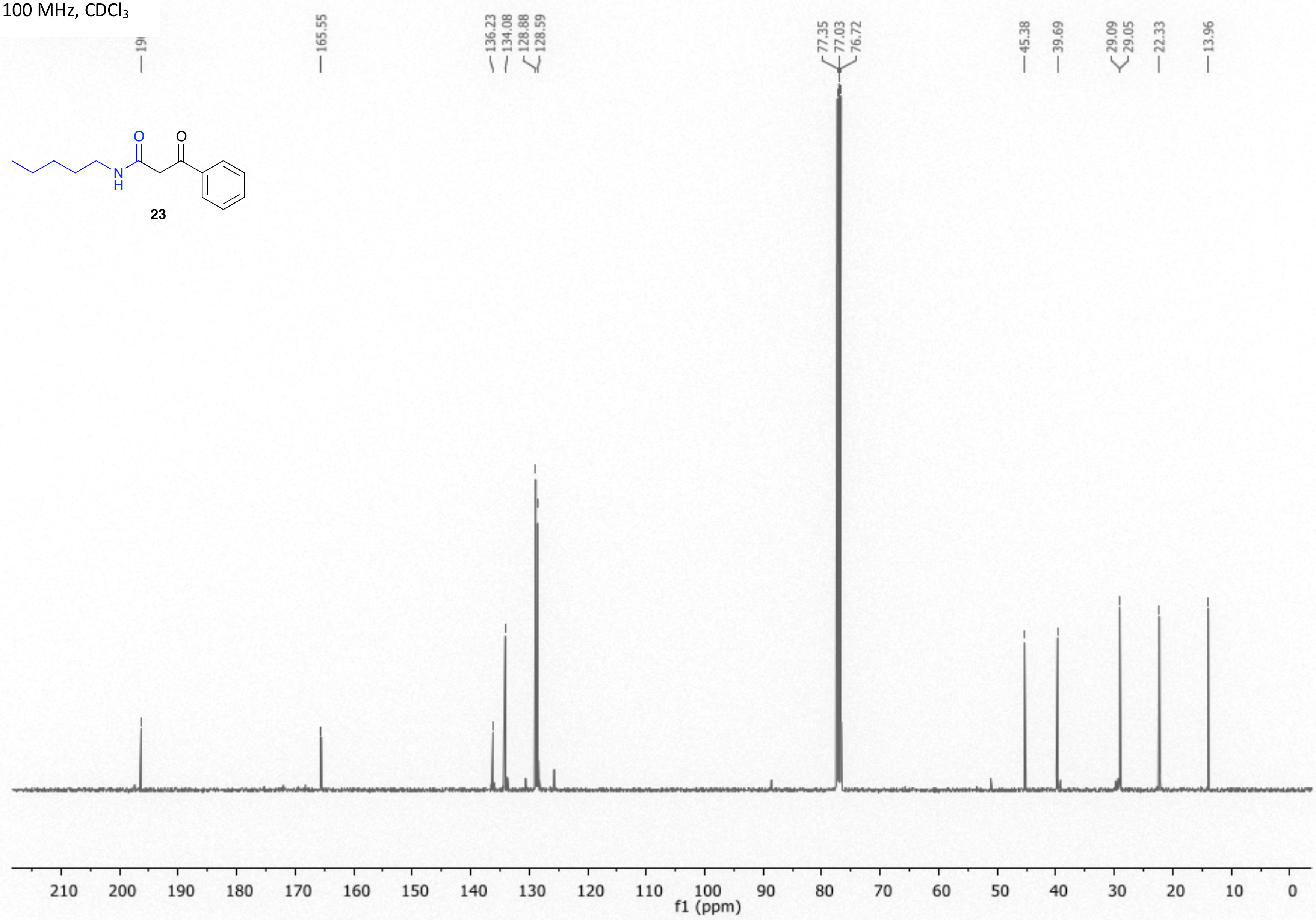

23

^{1}H NMR
400 MHz, $CDCl_3$

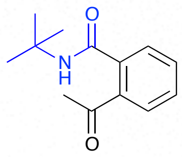

25

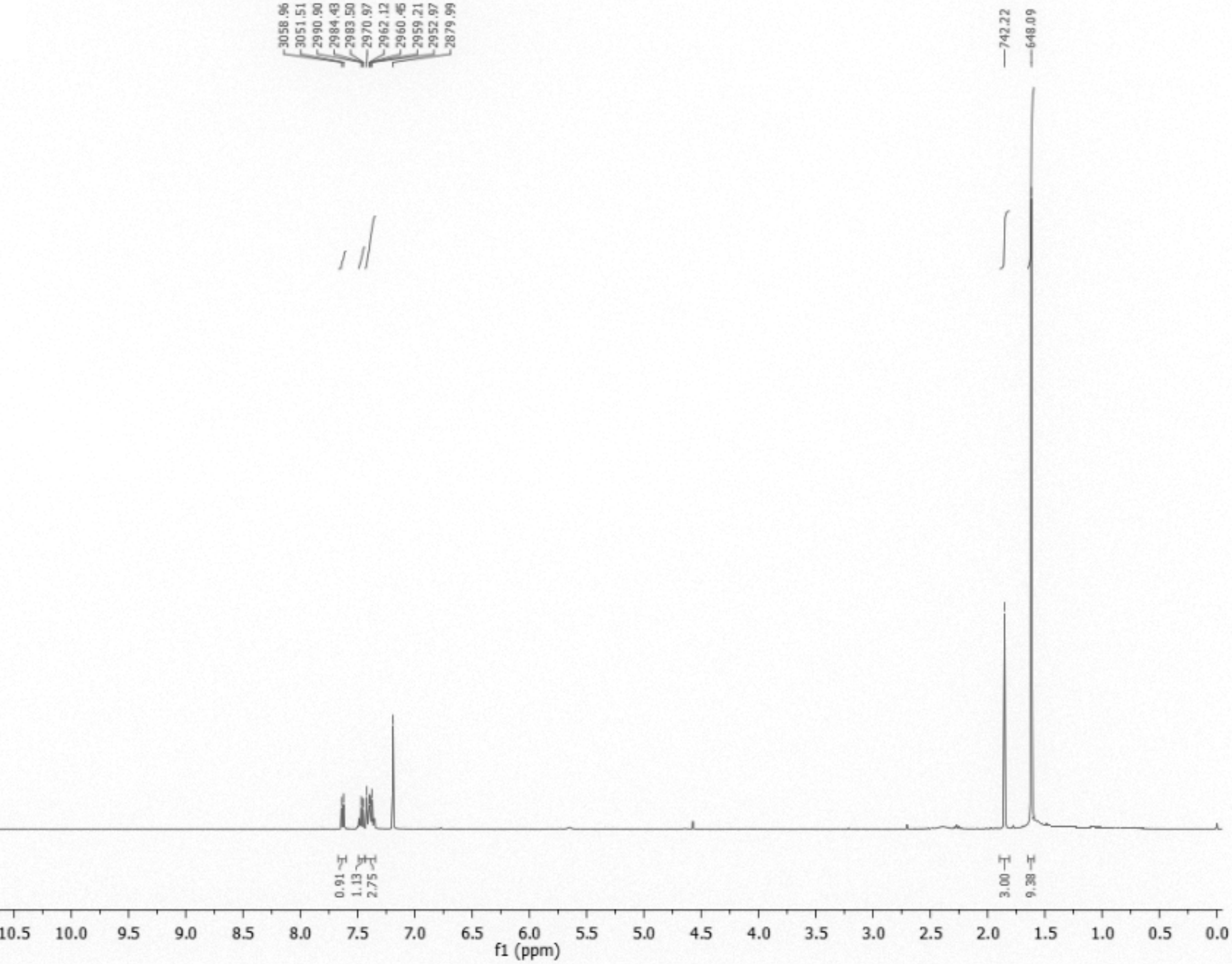

^{13}C NMR
100 MHz, $CDCl_3$

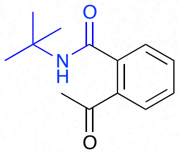

25

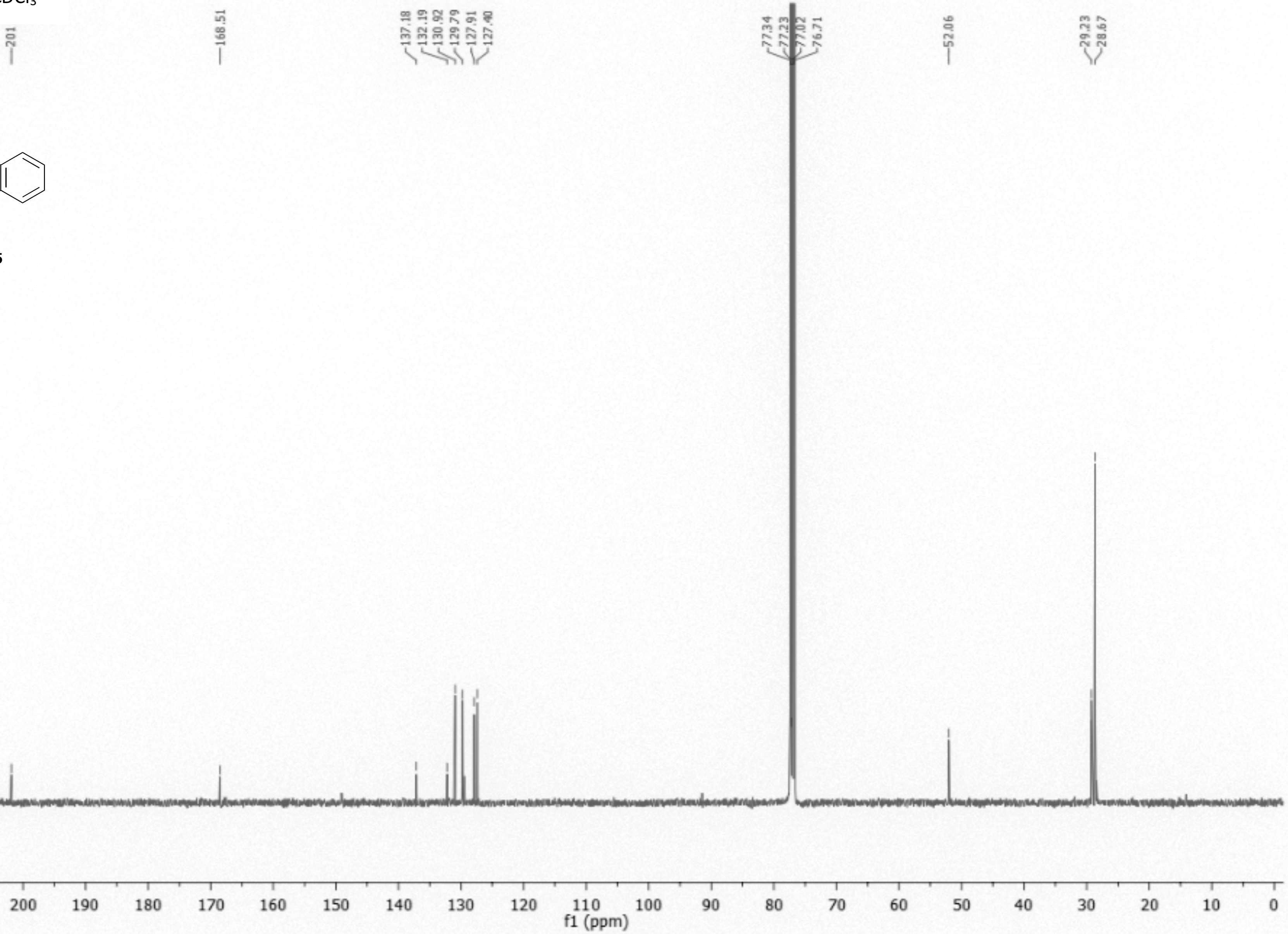

^{1}H NMR
400 MHz, CD_3OD

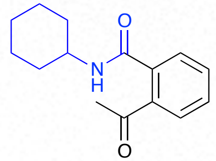

26

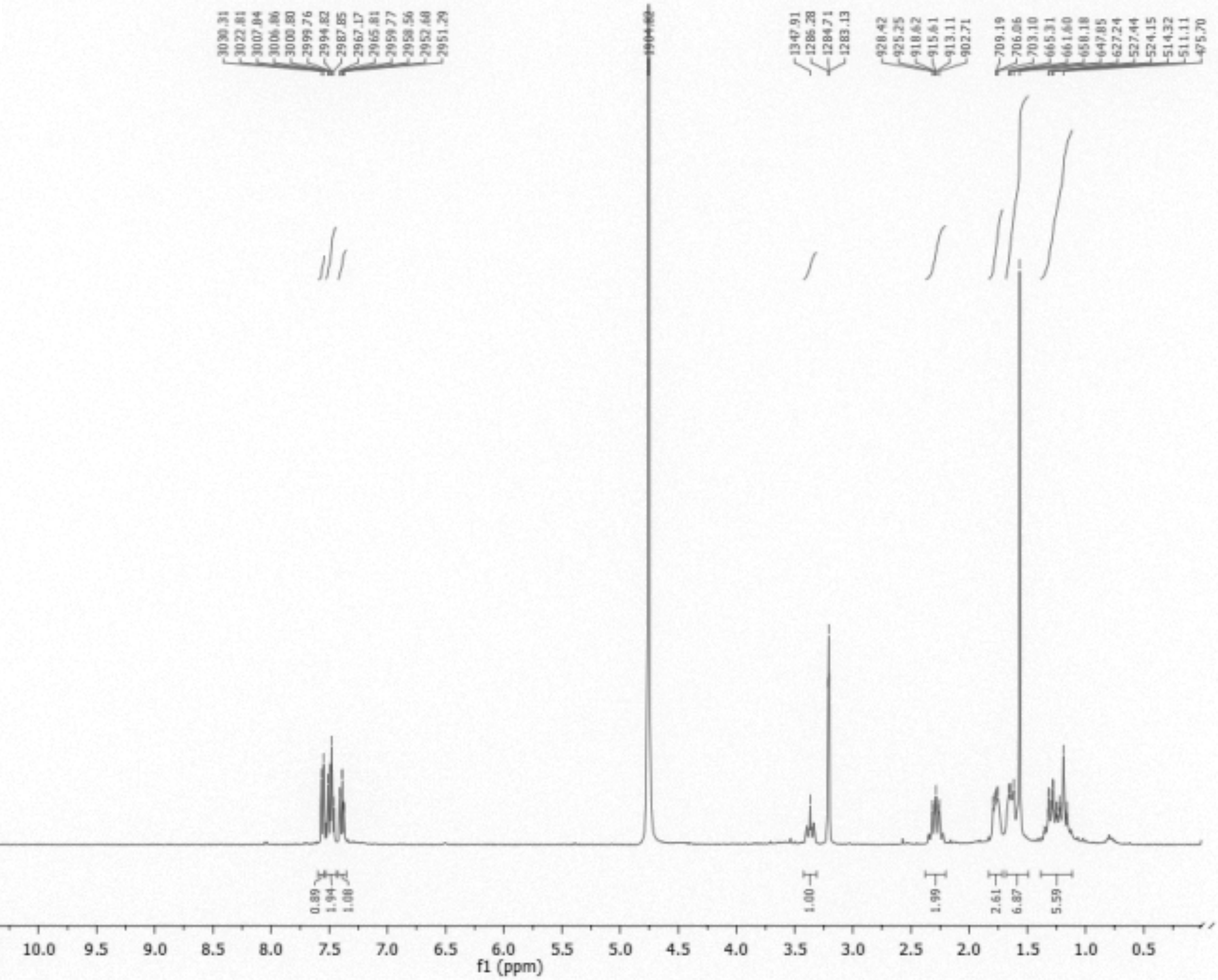

^{13}C NMR
100 MHz, CD_3OD

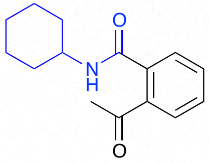

26

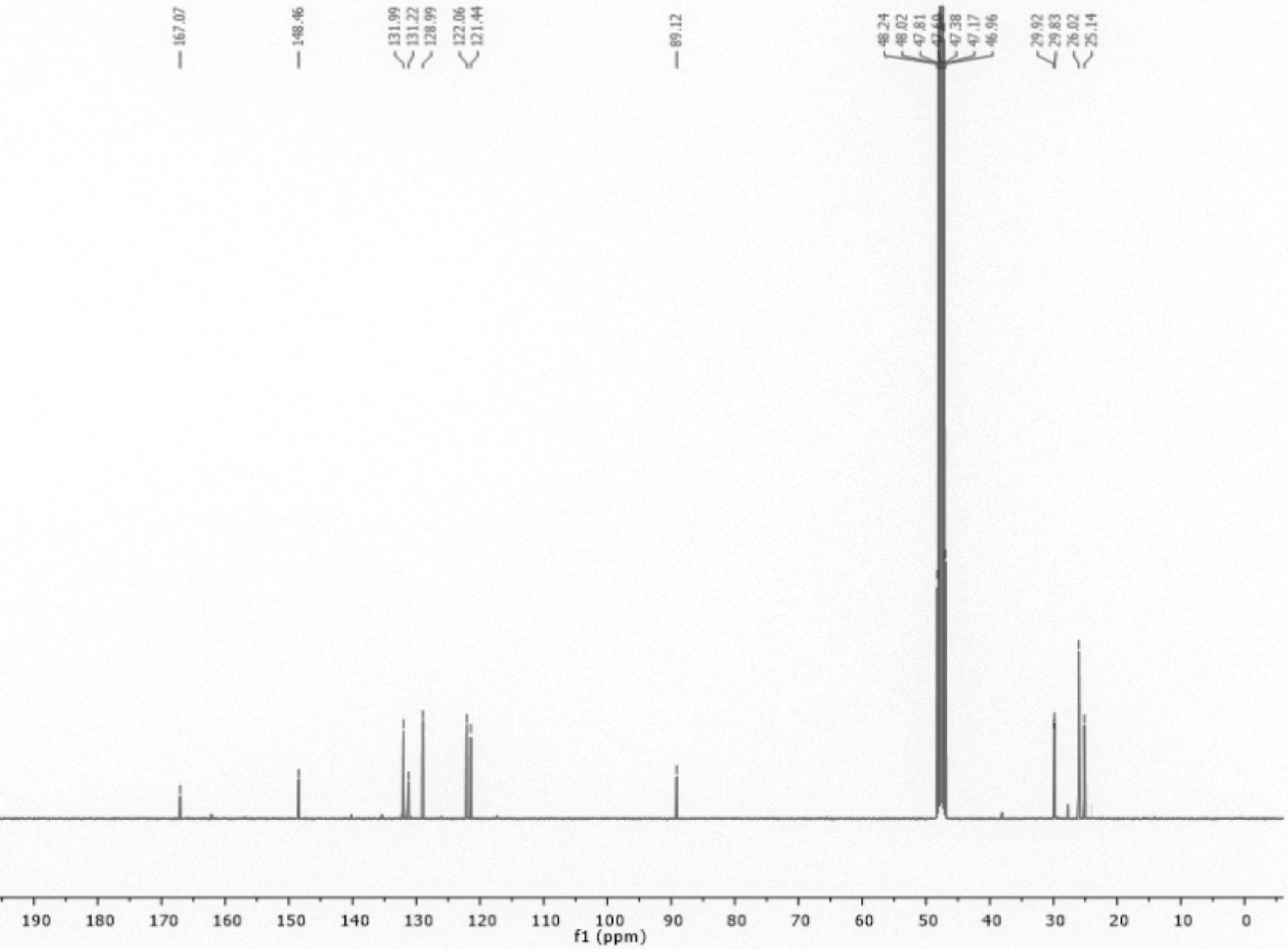

^{1}H NMR
400 MHz, $CDCl_3$

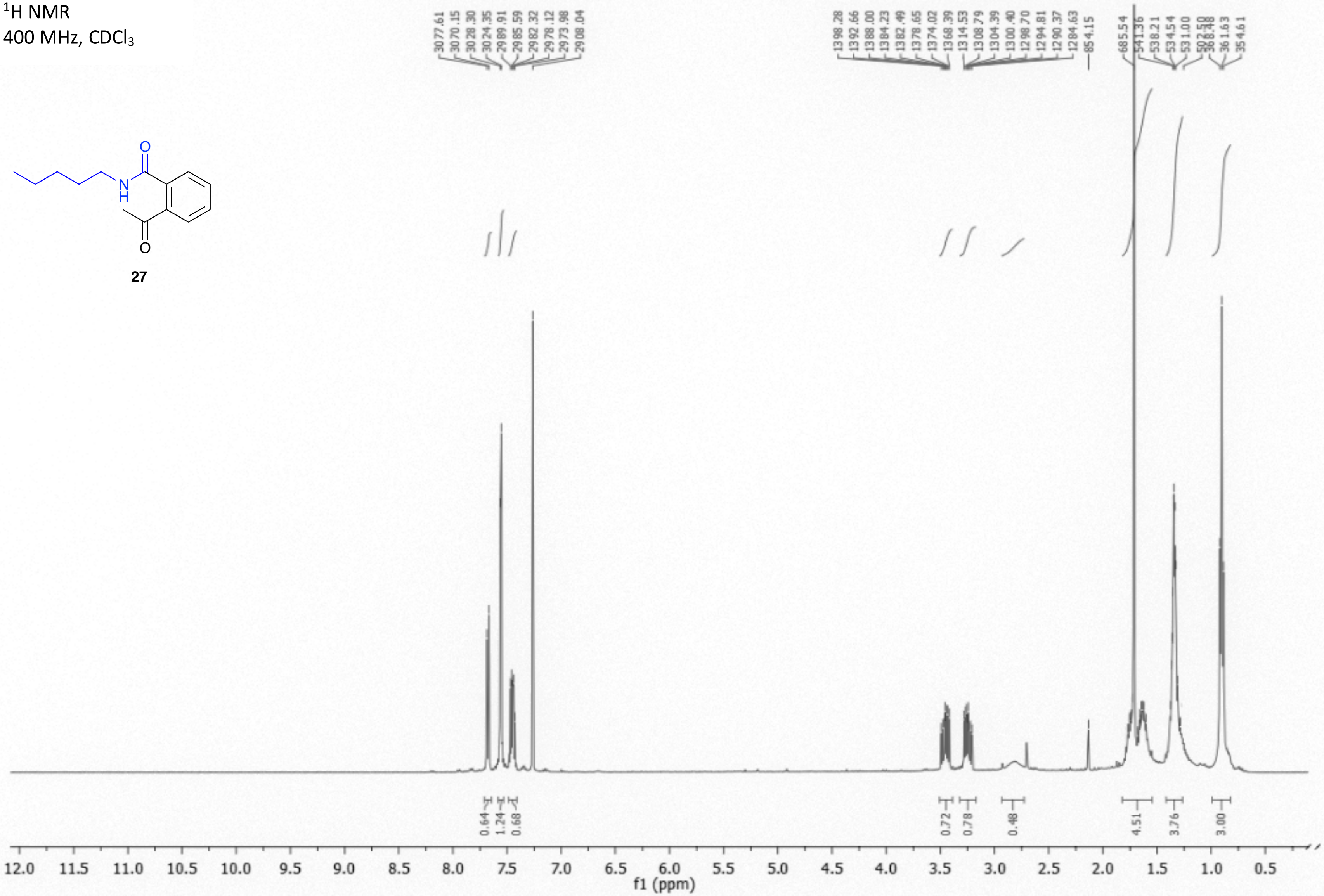

27

^{13}C NMR
100 MHz, $CDCl_3$

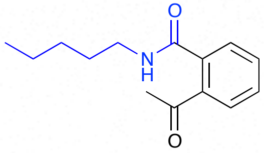

27

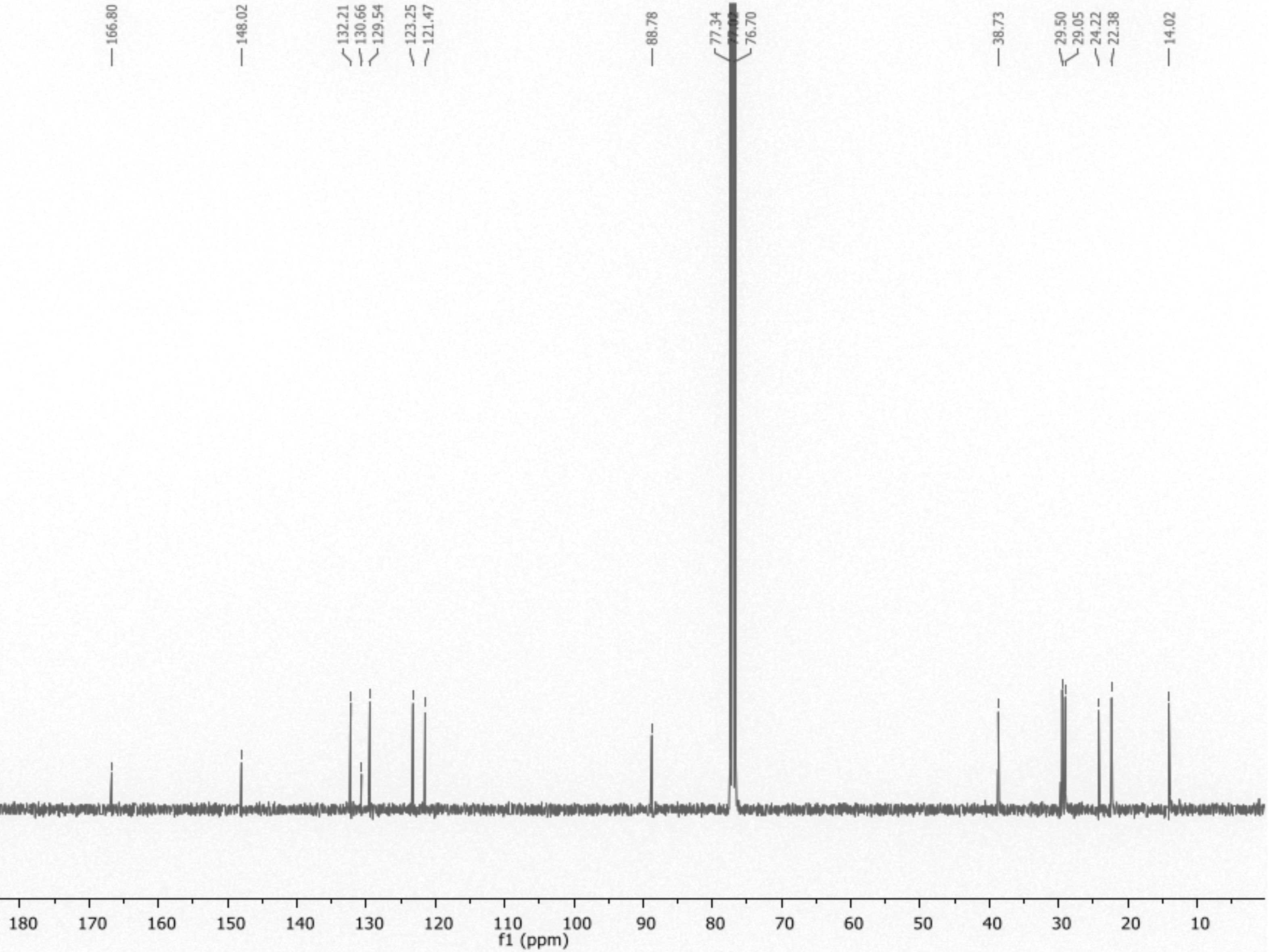

^{1}H NMR
400 MHz, $CDCl_3$

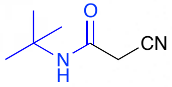

29

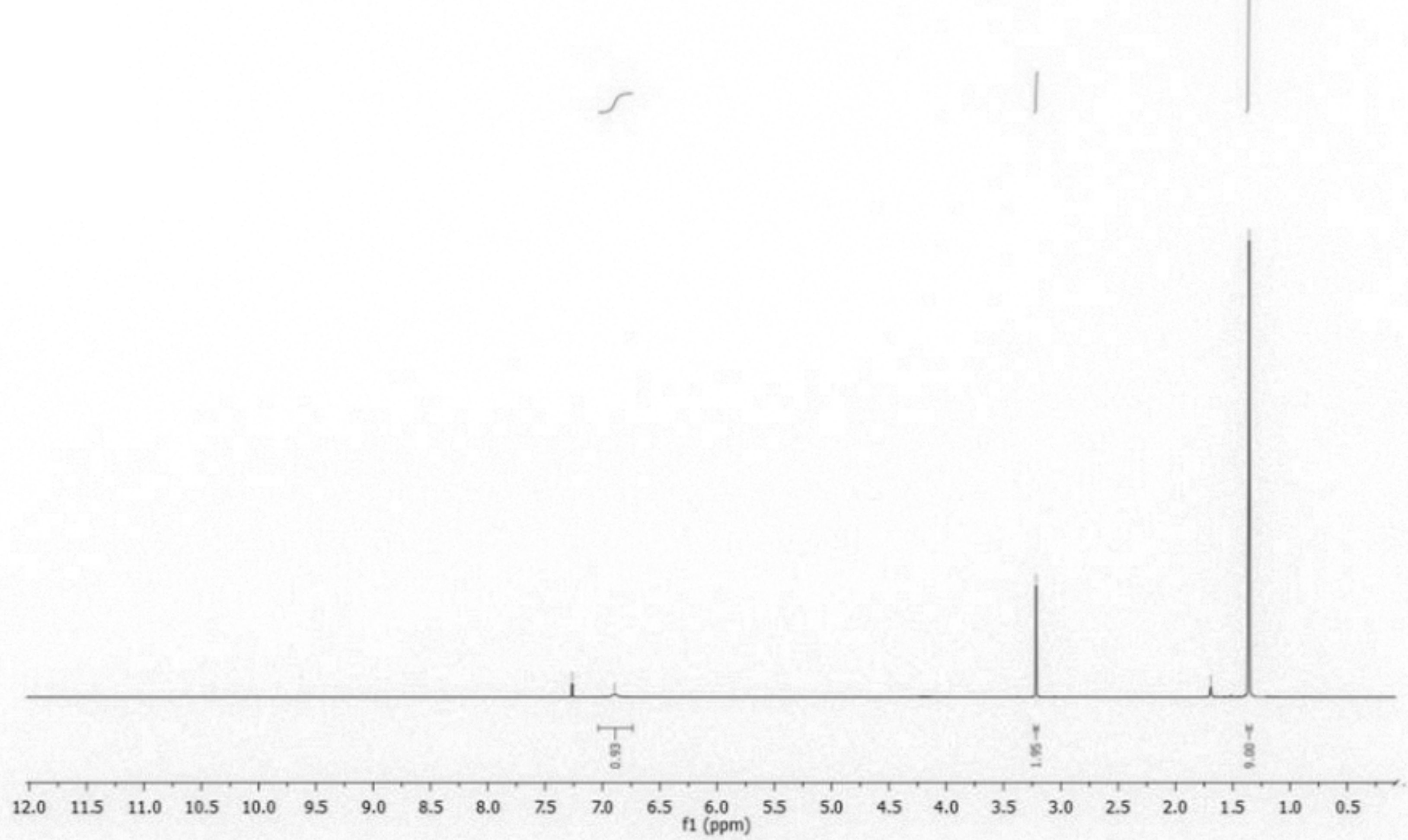

^{13}C NMR
100 MHz, $CDCl_3$

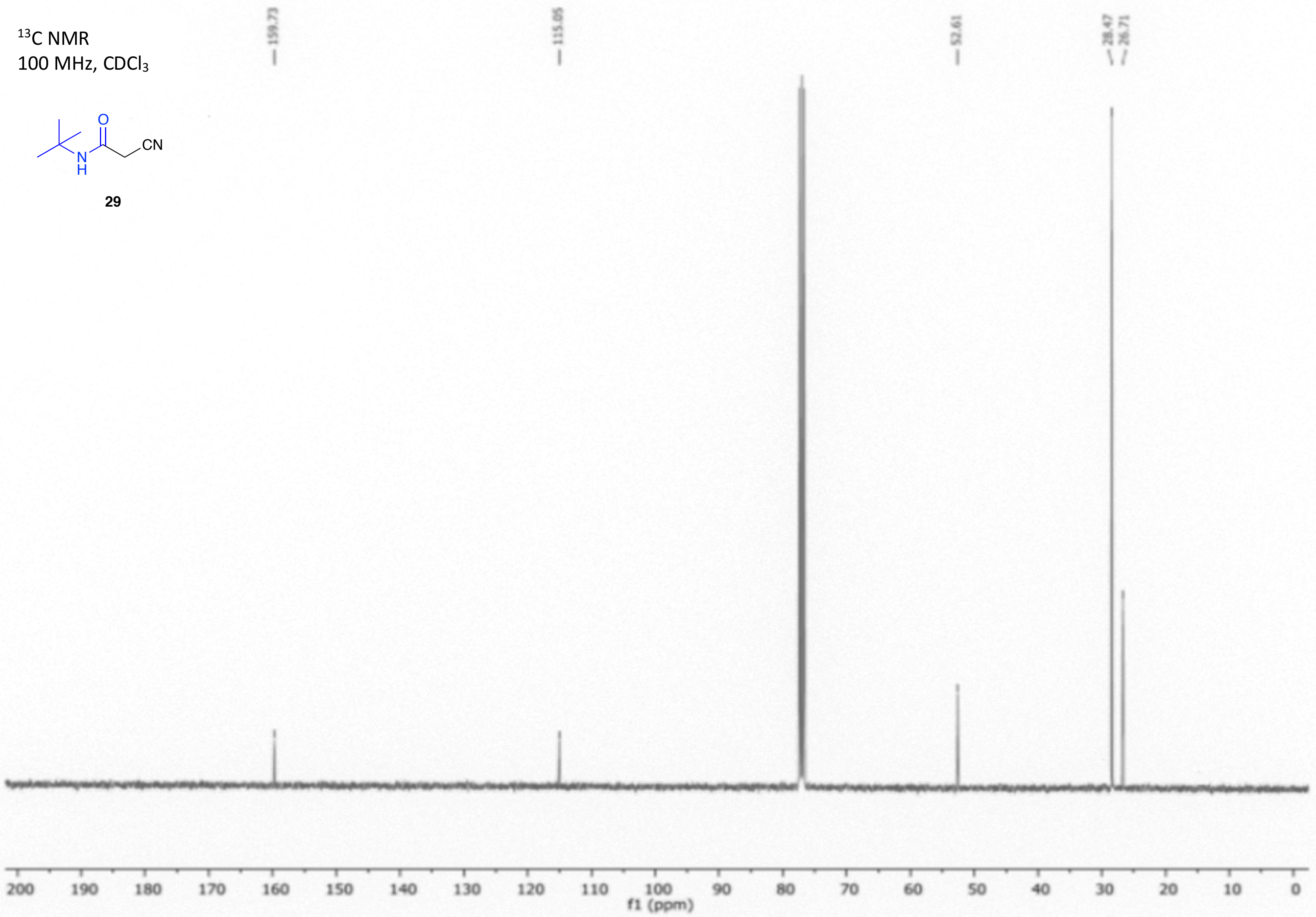

^{1}H NMR
400 MHz, DMSO-d_6

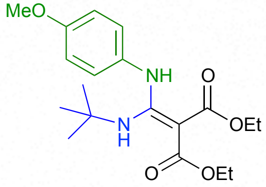
30

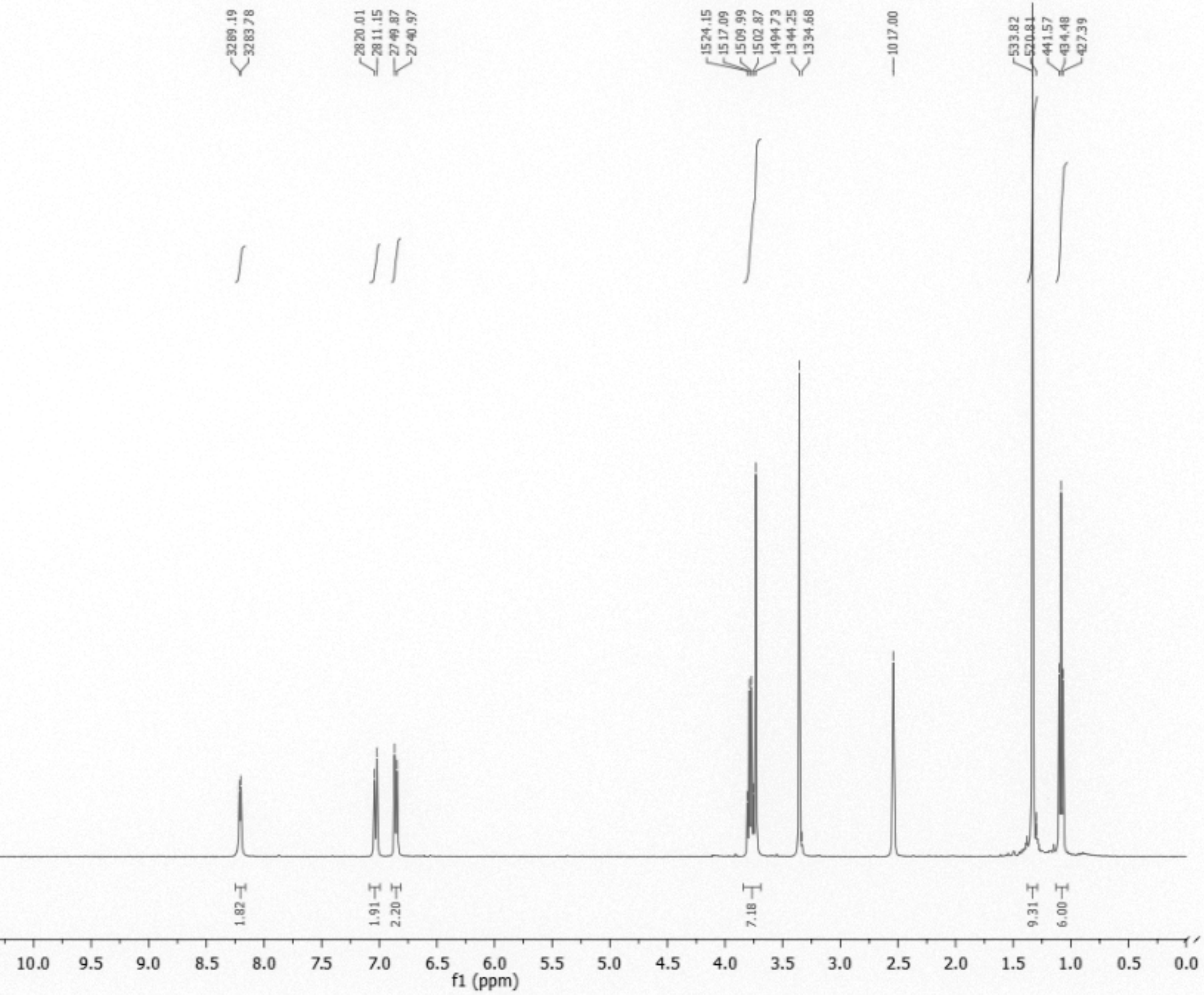

^{13}C NMR
100 MHz, DMSO-d_6

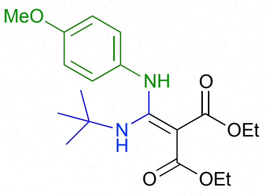

30

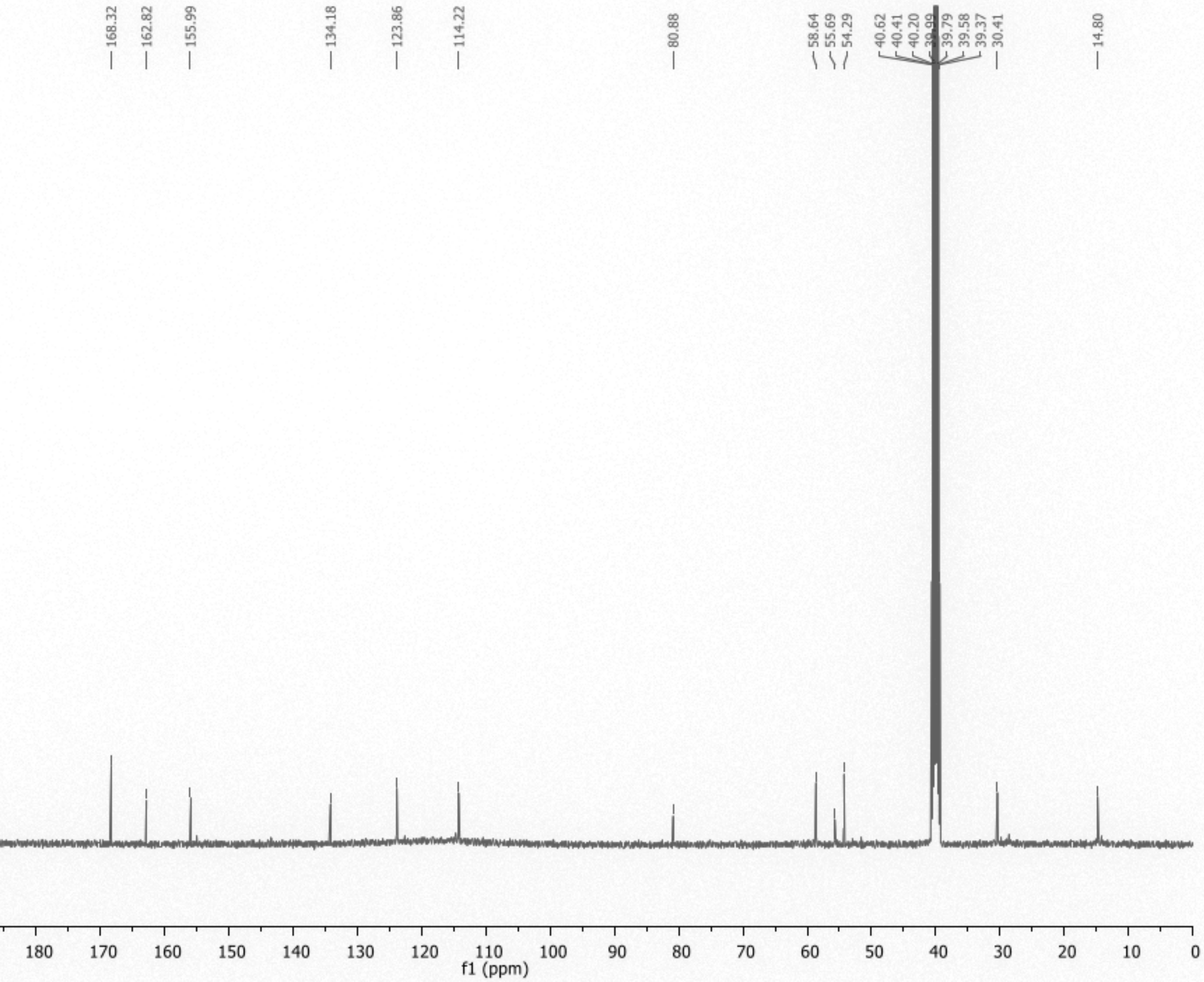

^{1}H NMR
400 MHz, DMSO-d_6

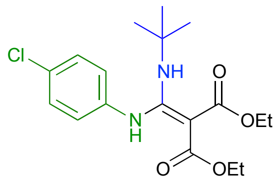

31

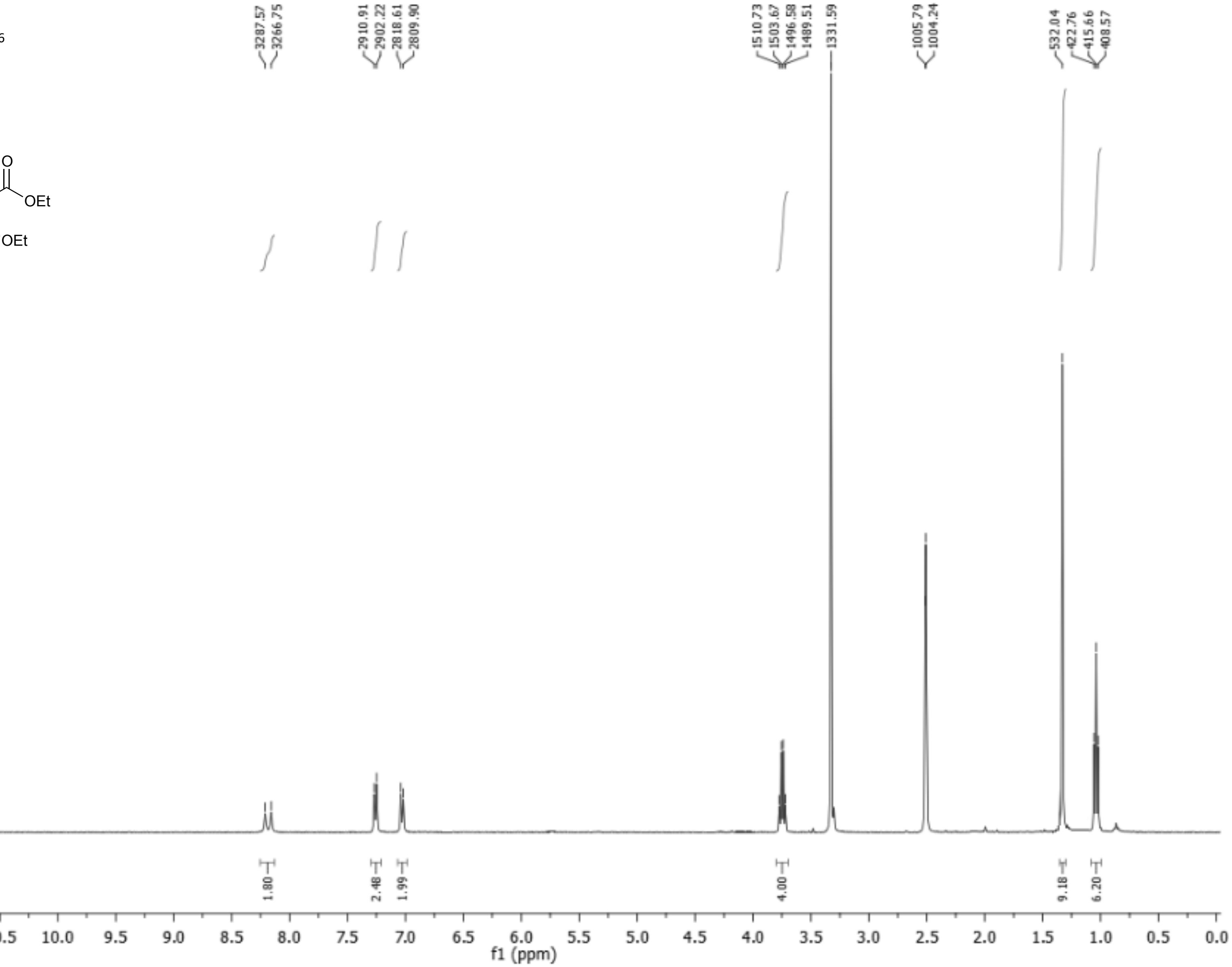

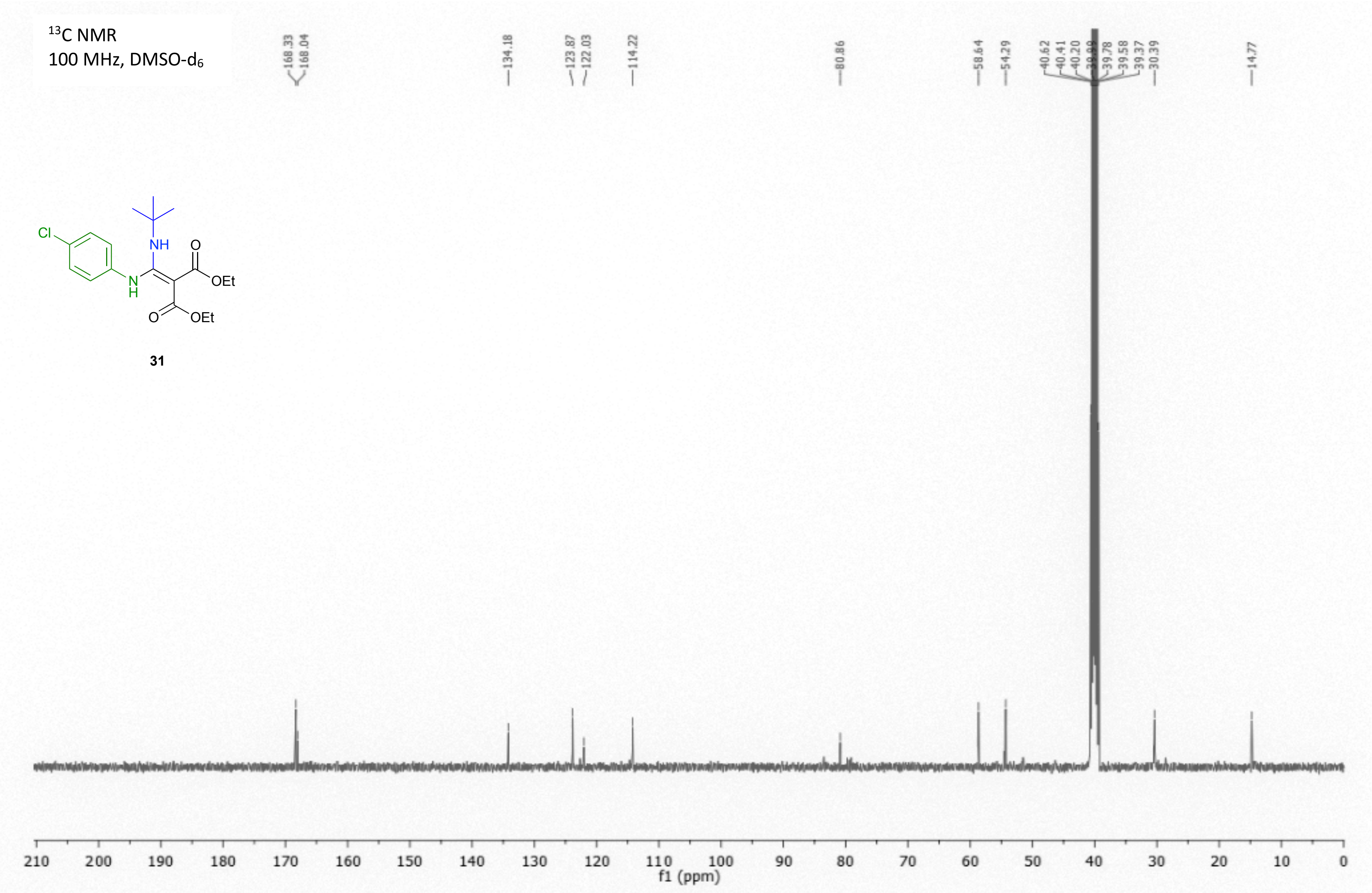
13C NMR
100 MHz, DMSO-d6
168.33
168.04
134.18
123.87
122.03
114.22
80.86
58.64
54.29
40.62
40.41
40.20
39.99
39.78
39.58
39.37
30.39
14.77
Cl
NH
O
OEt
N
H
O
OEt
31
210 200 190 180 170 160 150 140 130 120 110 100 90 80 70 60 50 40 30 20 10 0
f1 (ppm)

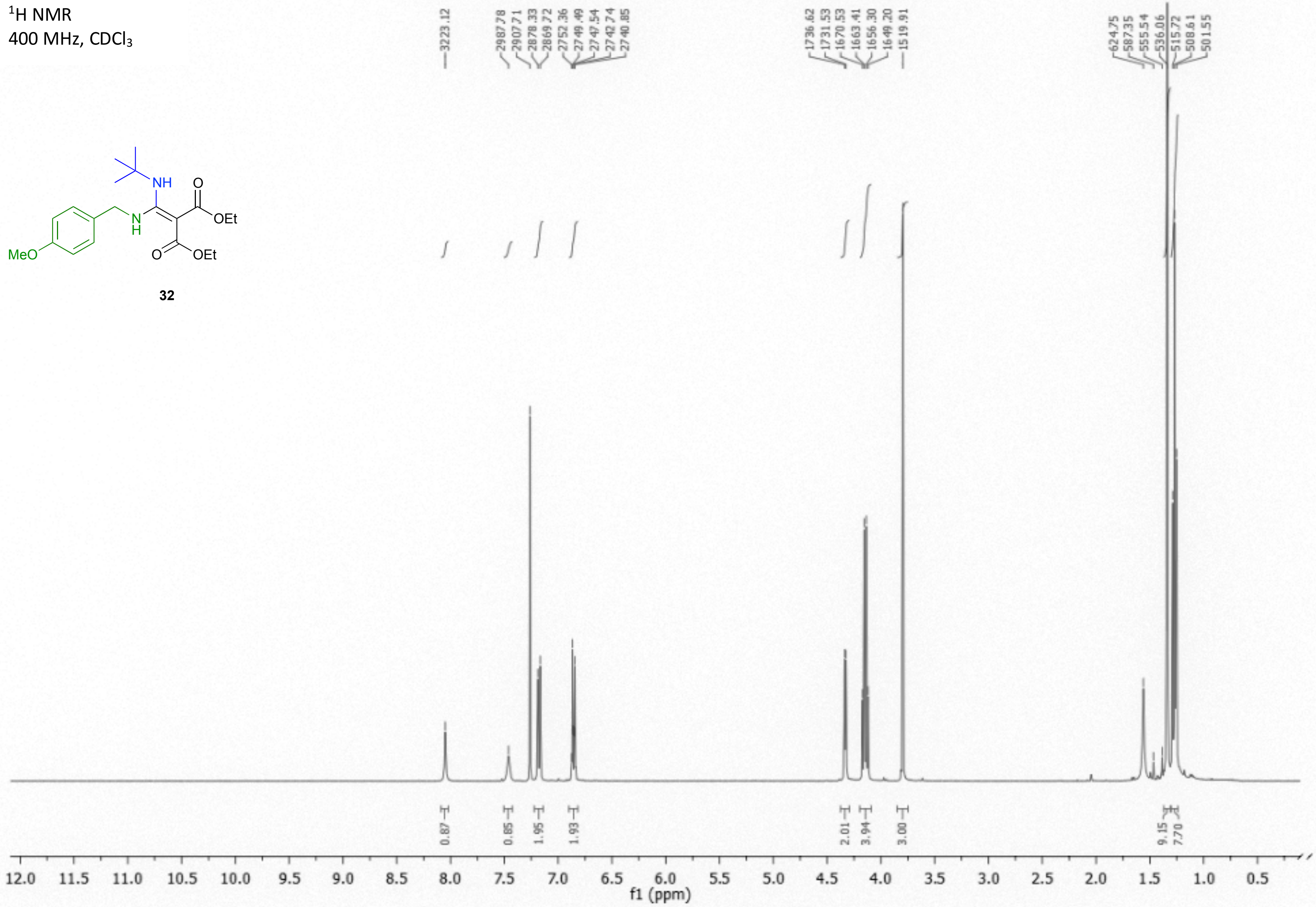
1H NMR
400 MHz, CDCl3
NH
O
OEt
MeO
N
H
O
OEt
32
3223.12
2987.78
2907.71
2878.33
2869.72
2752.36
2749.49
2747.54
2742.74
2740.85
1736.62
1731.53
1670.53
1663.41
1656.30
1649.20
1519.91
624.75
587.35
555.54
536.06
515.72
508.61
501.55
0.87
0.85
1.95
1.93
2.01
3.94
3.00
9.15
7.70
12.0 11.5 11.0 10.5 10.0 9.5 9.0 8.5 8.0 7.5 7.0 6.5 6.0 5.5 5.0 4.5 4.0 3.5 3.0 2.5 2.0 1.5 1.0 0.5
f1 (ppm)

^{13}C NMR
100 MHz, $CDCl_3$

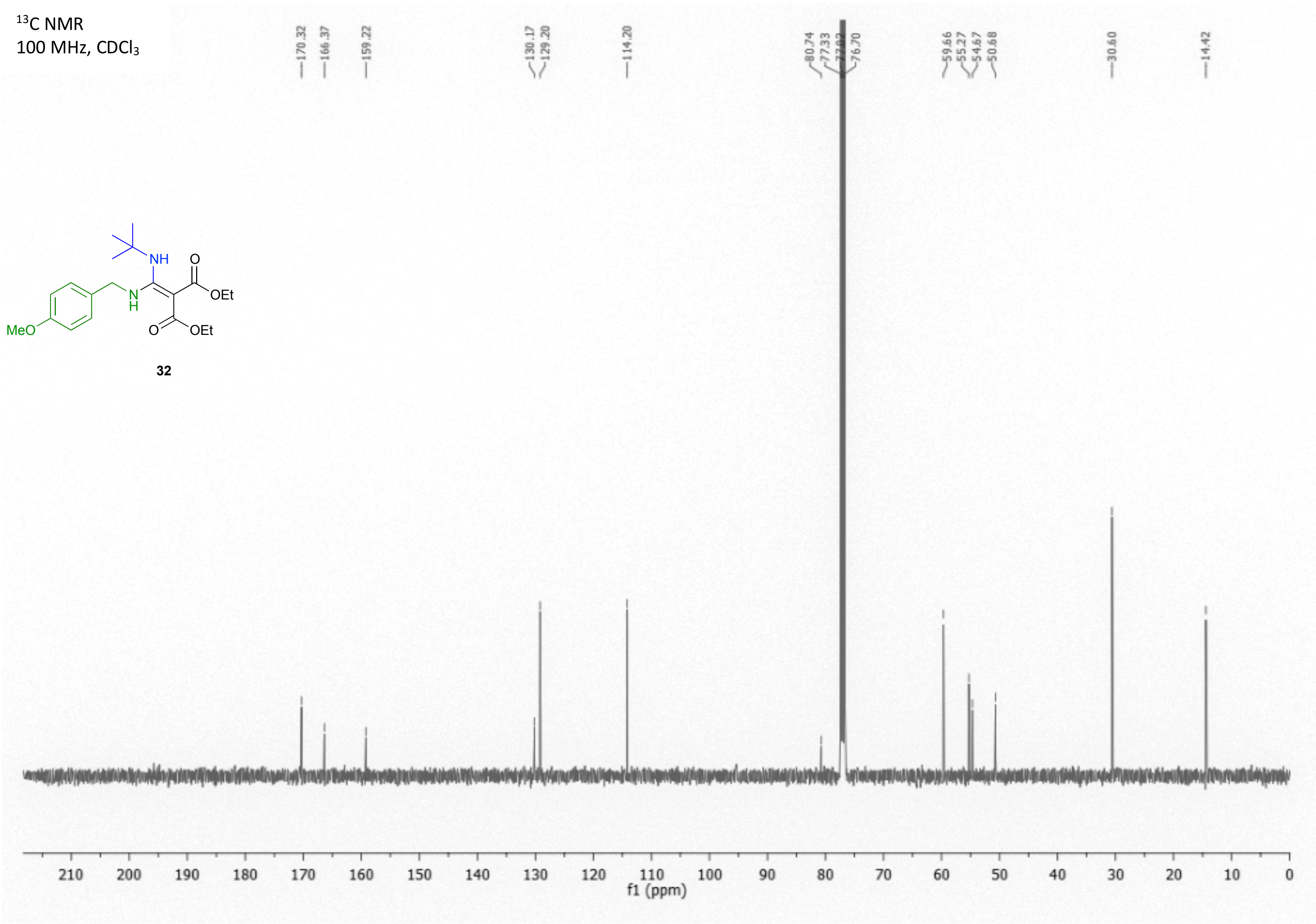

^{1}H NMR
400 MHz, $CDCl_3$

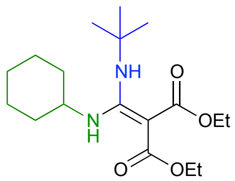

33

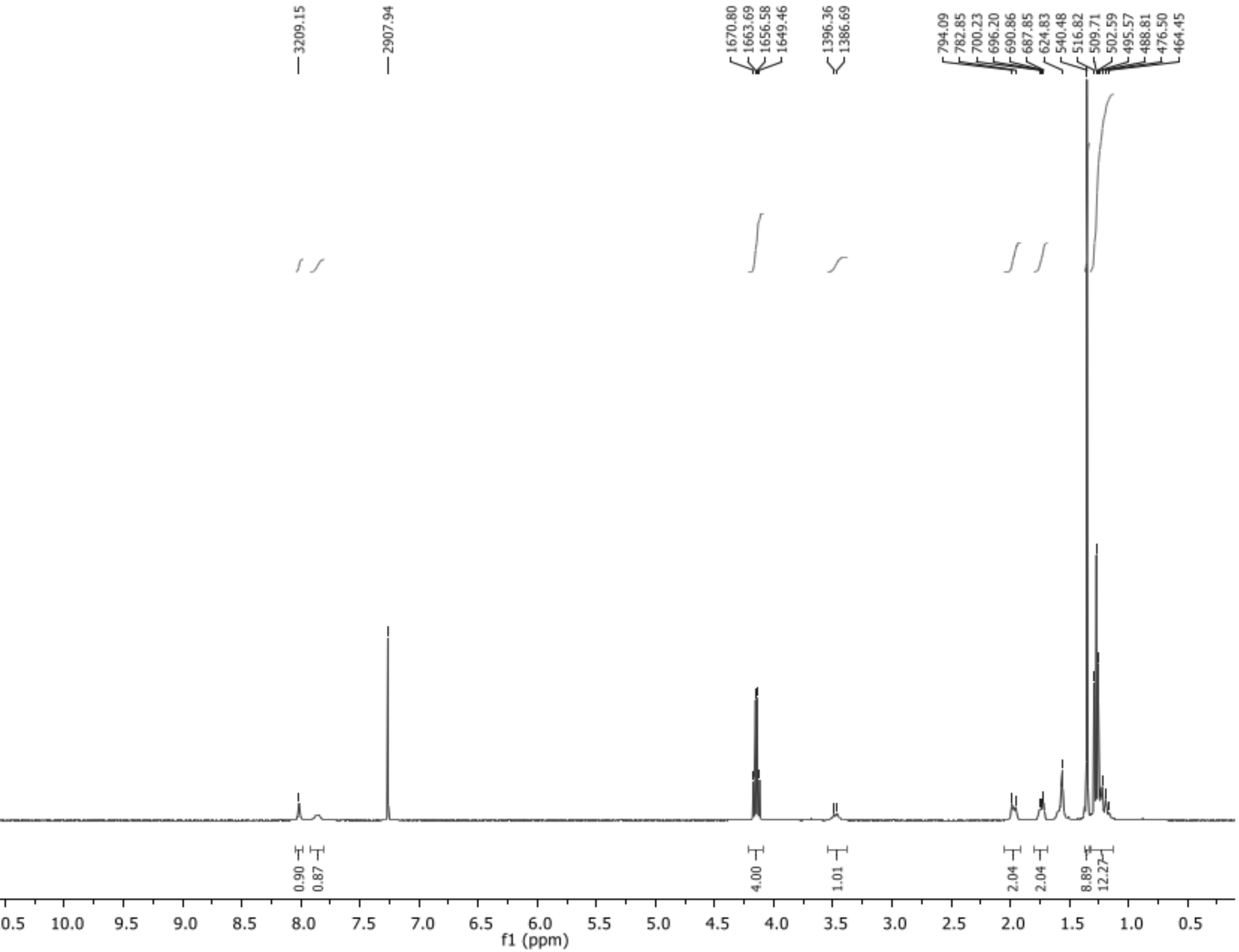

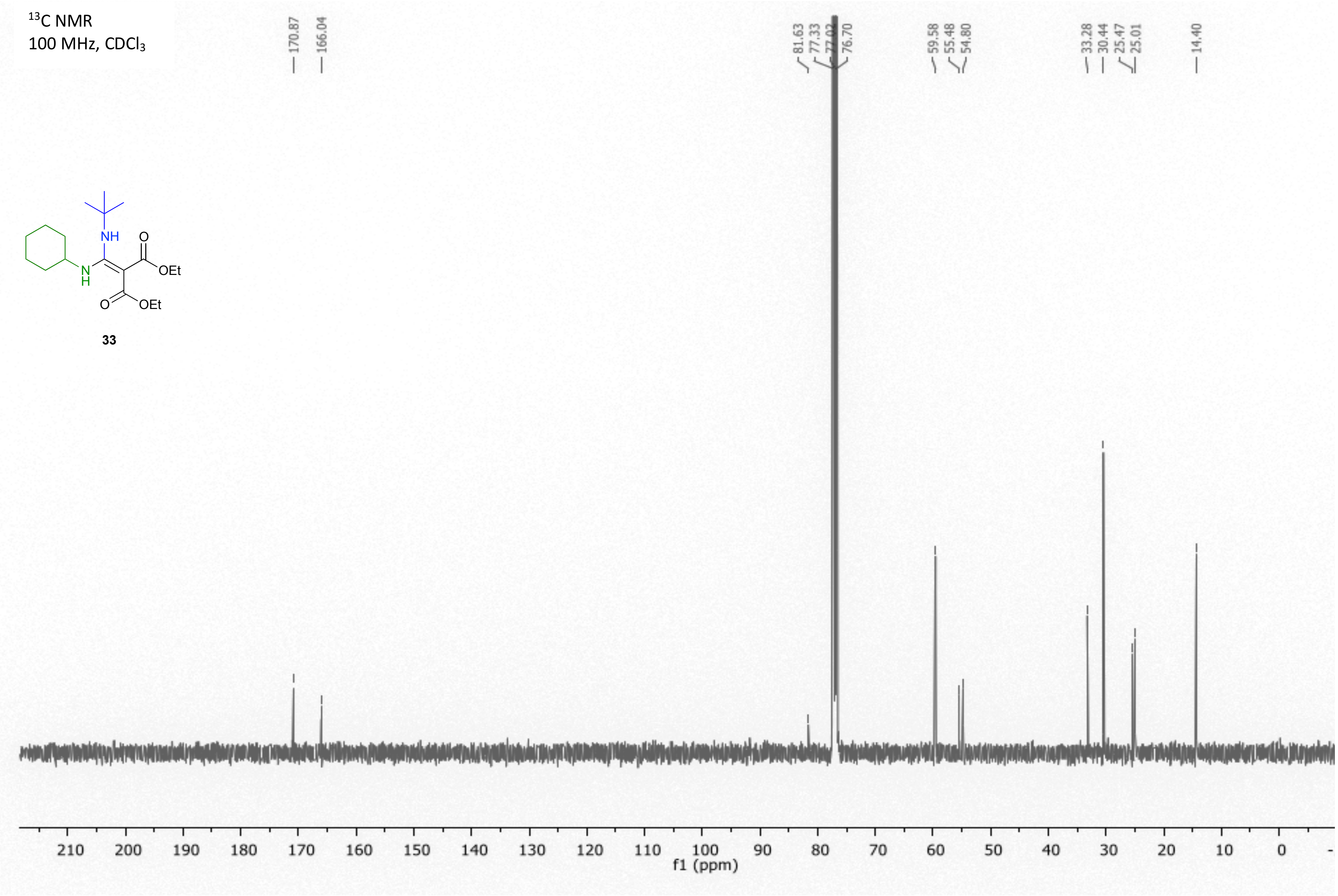

13C NMR
100 MHz, CDCl3
170.87
166.04
81.63
77.33
77.02
76.70
59.58
55.48
54.80
33.28
30.44
25.47
25.01
14.40
NH
N
H
O
OEt
O
OEt
33
210
200
190
180
170
160
150
140
130
120
110
100
90
80
70
60
50
40
30
20
10
0
f1 (ppm)

^{1}H NMR
400 MHz, $CDCl_3$

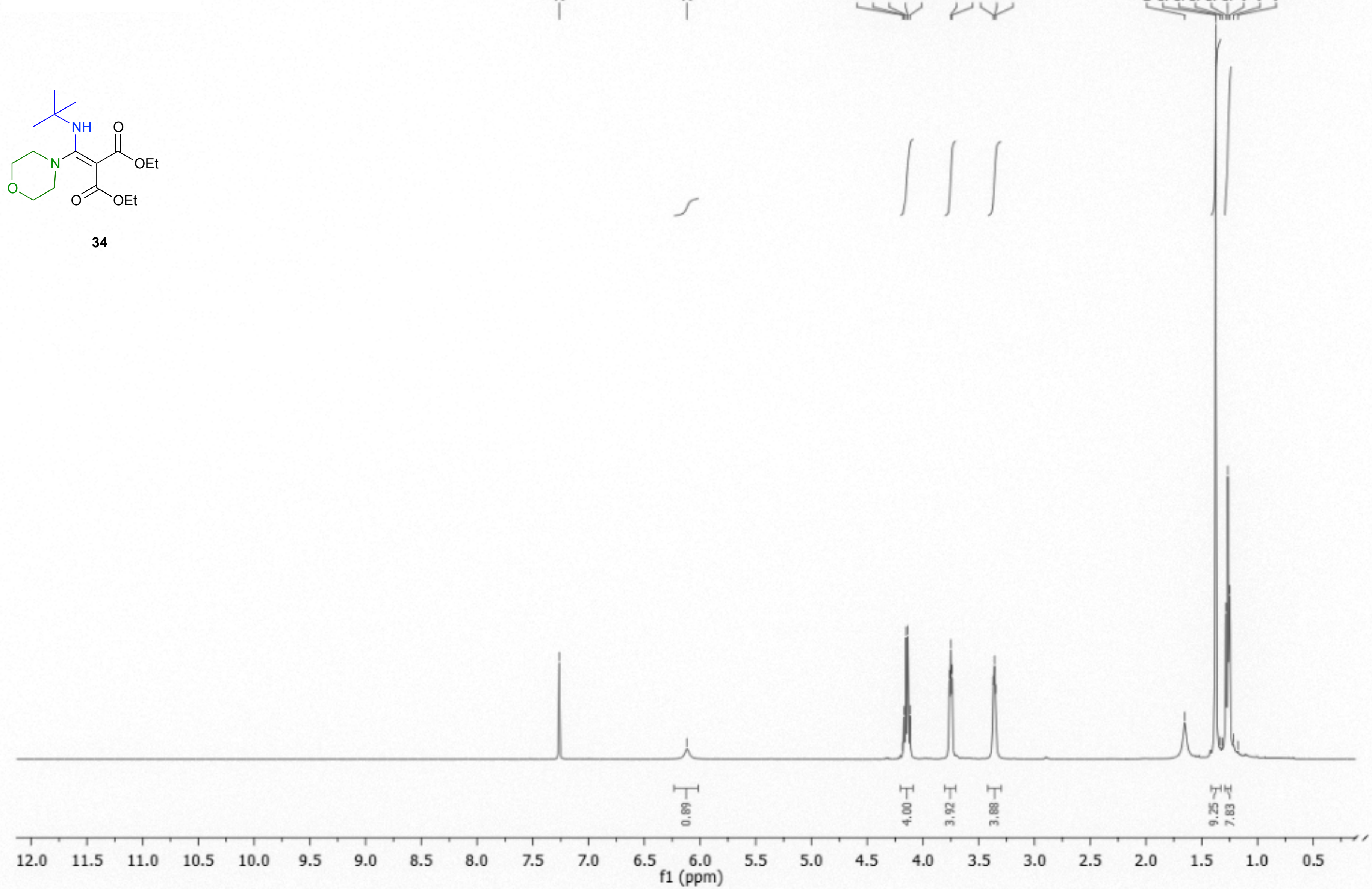

^{13}C NMR
100 MHz, $CDCl_3$

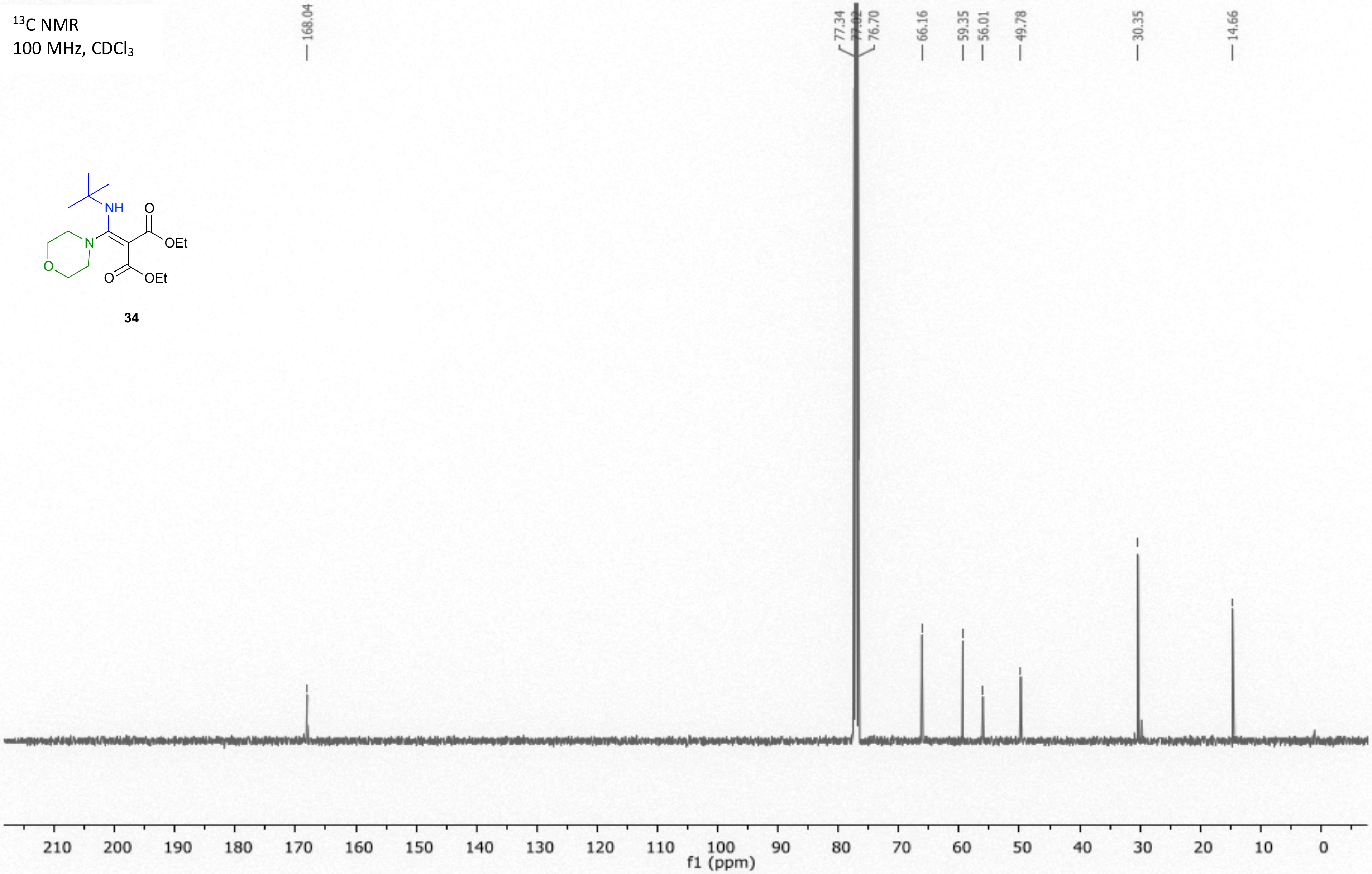

34

^{1}H NMR
400 MHz, $CDCl_3$

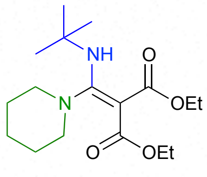

35

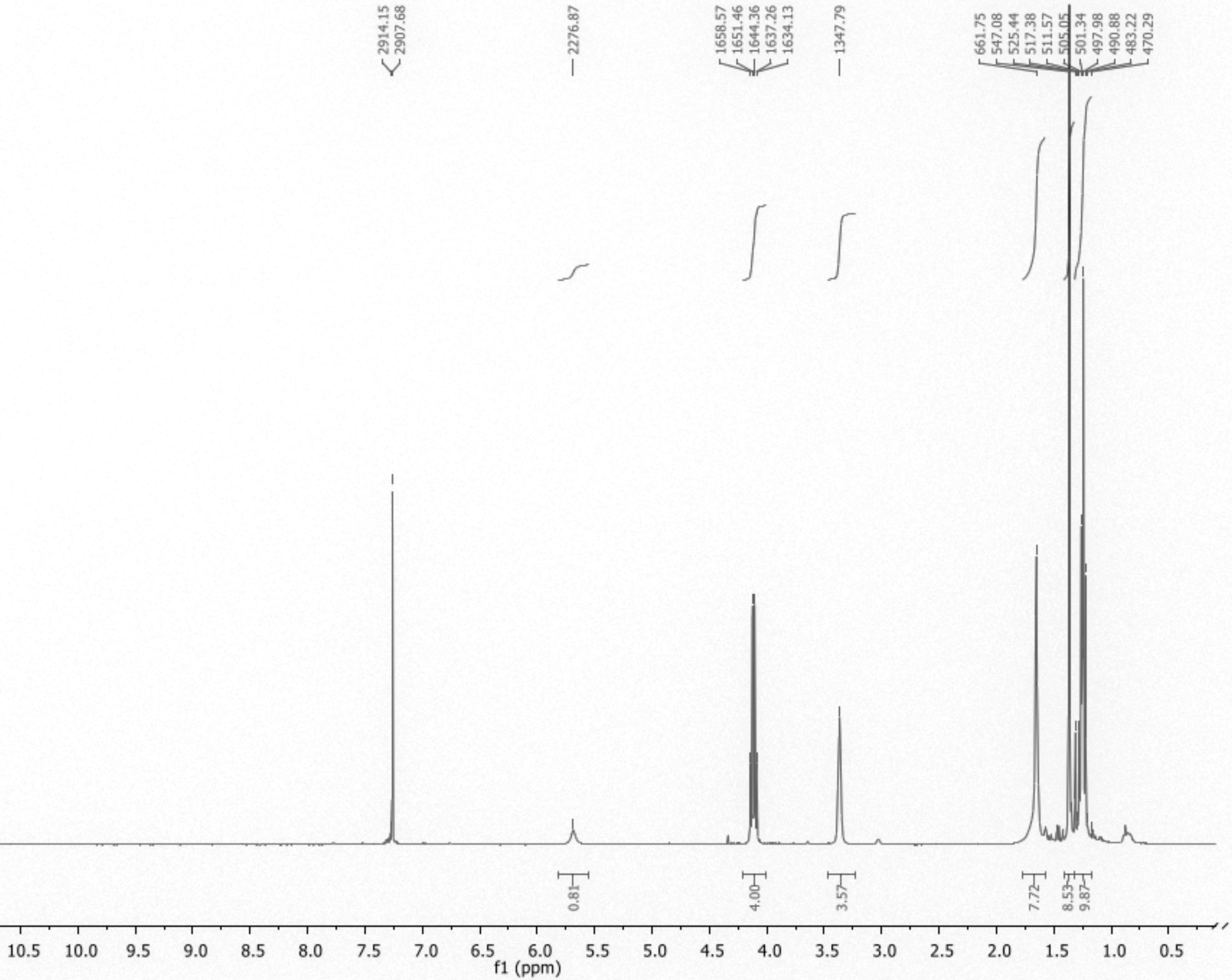

^{13}C NMR
100 MHz, $CDCl_3$

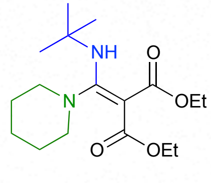

35

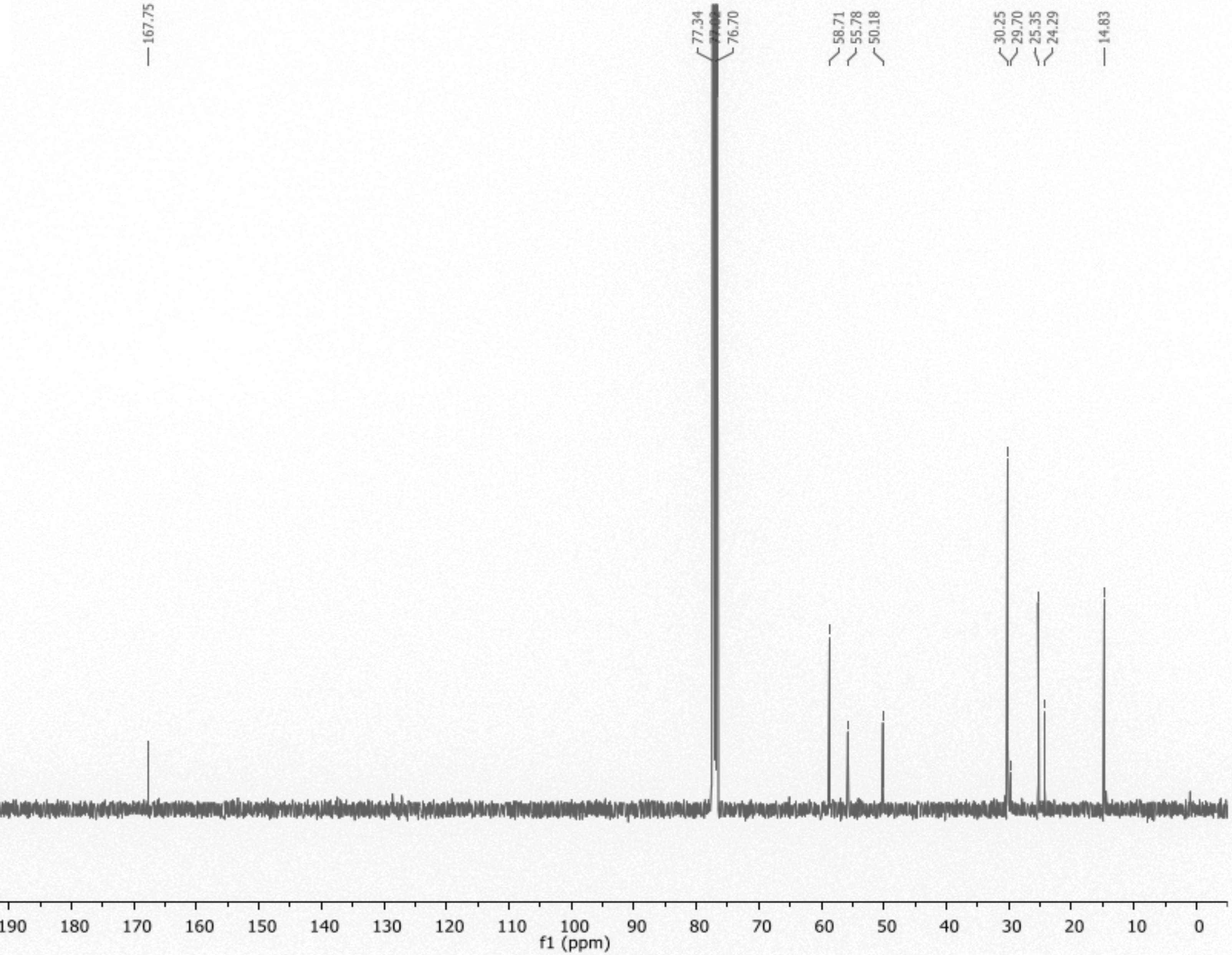

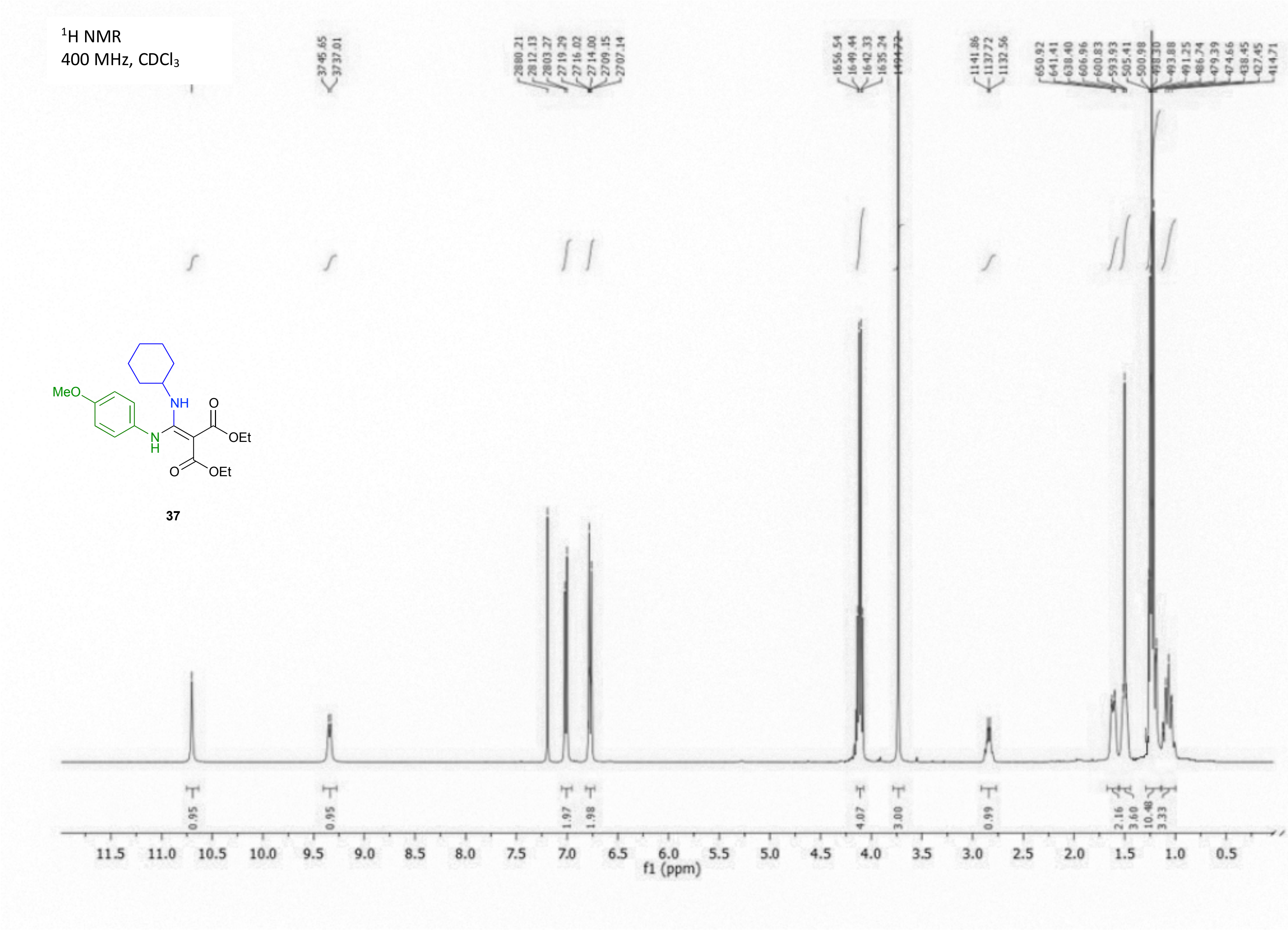

1H NMR
400 MHz, CDCl3
MeO
NH
O
OEt
N
H
O
OEt
37
0.95
0.95
1.97
1.98
4.07
3.00
0.99
2.16
3.60
10.48
3.33
f1 (ppm)

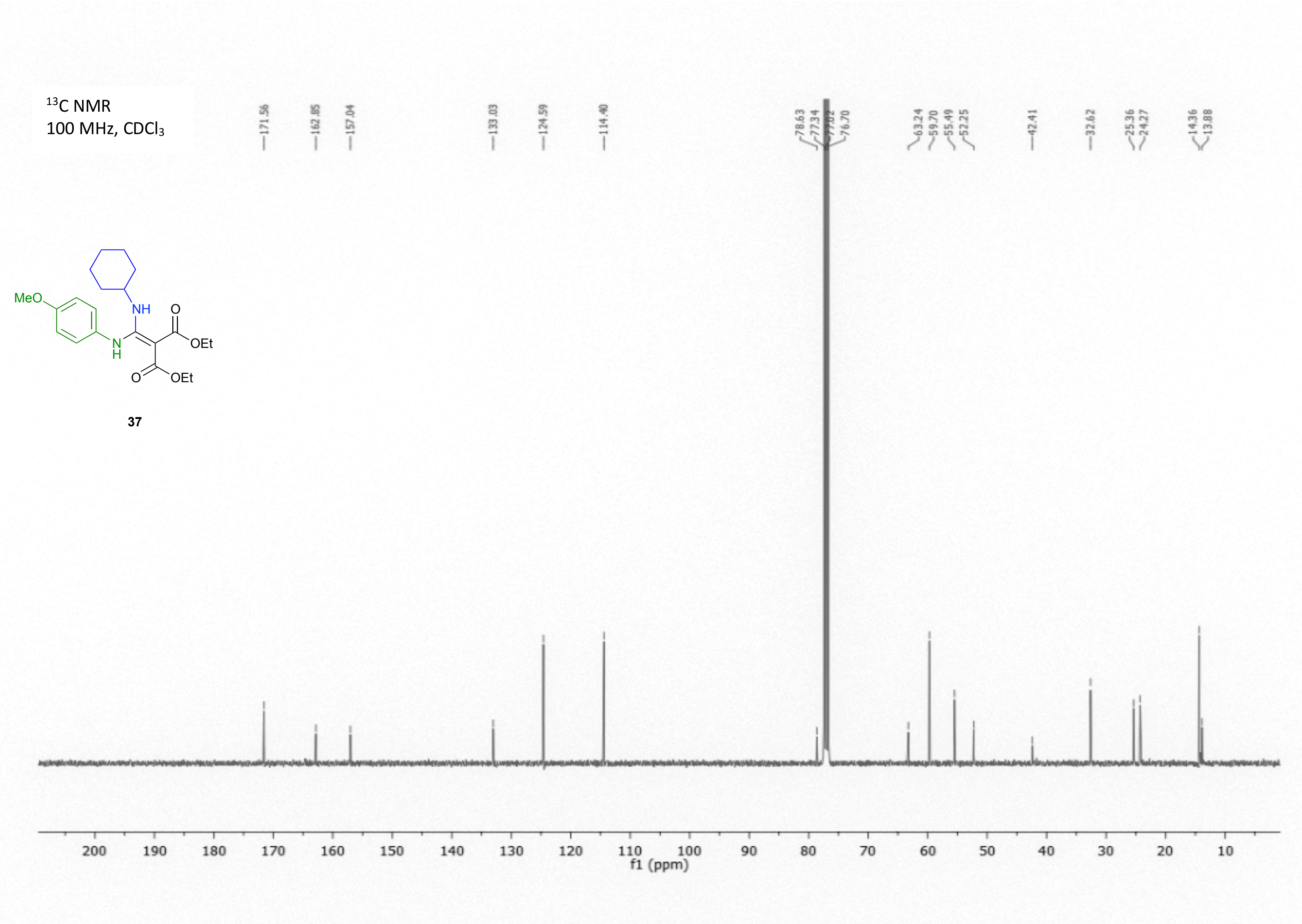

^{13}C NMR
100 MHz, $CDCl_3$
171.56
162.85
157.04
133.03
124.59
114.40
78.63
77.34
77.02
76.70
63.24
59.70
55.49
52.25
42.41
32.62
25.36
24.27
14.36
13.88
MeO
NH
N
H
O
OEt
O
OEt
37
200
190
180
170
160
150
140
130
120
110
100
90
80
70
60
50
40
30
20
10
f1 (ppm)

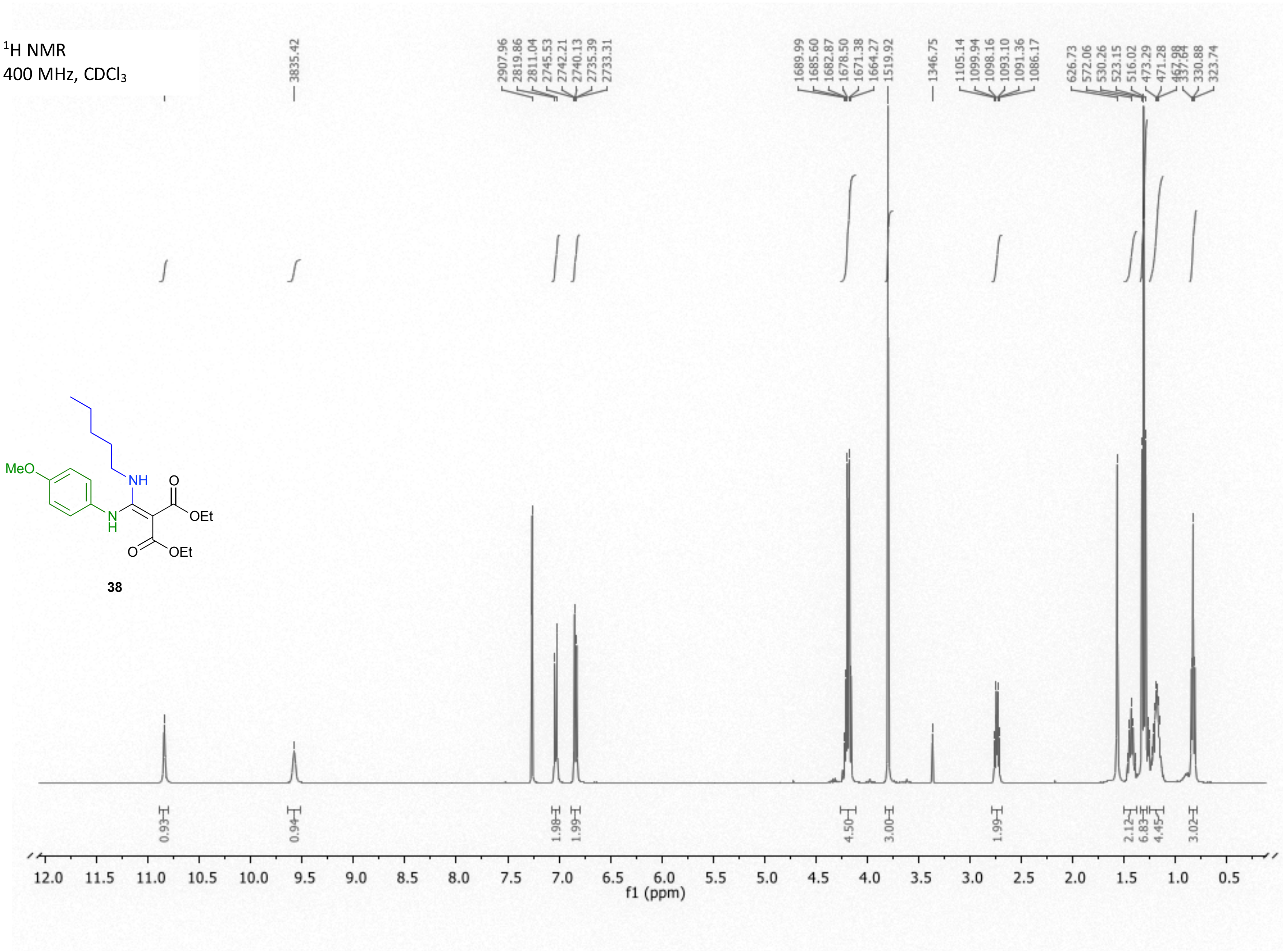
¹H NMR
400 MHz, CDCl₃
3835.42
2907.96
2819.86
2811.04
2745.53
2742.21
2740.13
2735.39
2733.31
1689.99
1685.60
1682.87
1678.50
1671.38
1664.27
1519.92
1346.75
1105.14
1099.94
1098.16
1093.10
1091.36
1086.17
626.73
572.06
530.26
523.15
516.02
473.29
471.28
330.88
323.74
MeO
NH
H
N
O
OEt
O
OEt
38
0.93
0.94
1.98
1.99
4.50
3.00
1.99
2.12
6.83
4.45
3.02
12.0
11.5
11.0
10.5
10.0
9.5
9.0
8.5
8.0
7.5
7.0
6.5
6.0
5.5
5.0
4.5
4.0
3.5
3.0
2.5
2.0
1.5
1.0
0.5
f1 (ppm)

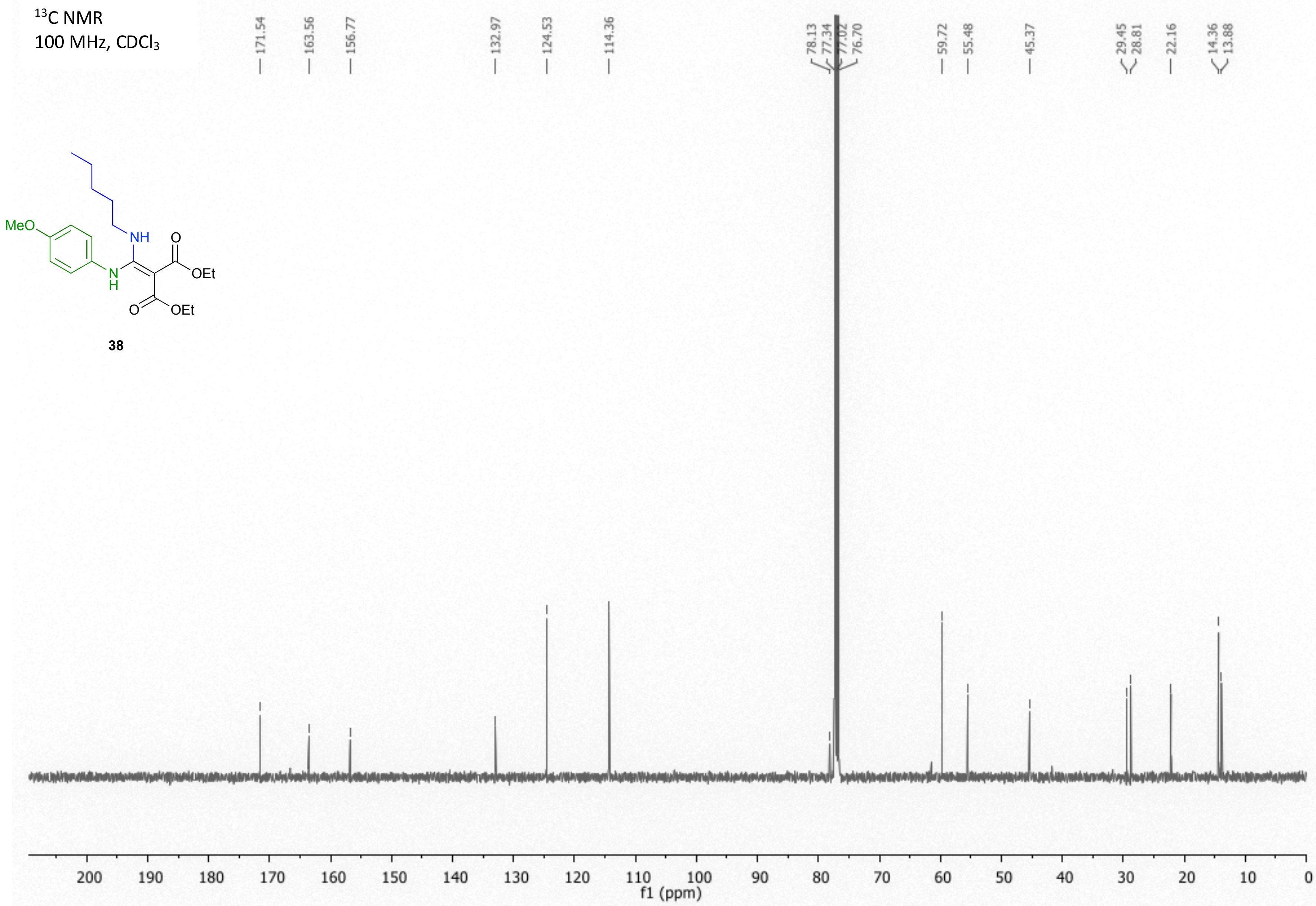

^{13}C NMR
100 MHz, CDCl$_3$
171.54
163.56
156.77
132.97
124.53
114.36
78.13
77.34
77.02
76.70
59.72
55.48
45.37
29.45
28.81
22.16
14.36
13.88
MeO
NH
O
OEt
N
H
O
OEt
38
200 190 180 170 160 150 140 130 120 110 100 90 80 70 60 50 40 30 20 10 0
f1 (ppm)

^{1}H NMR
400 MHz, DMSO-d$_6$

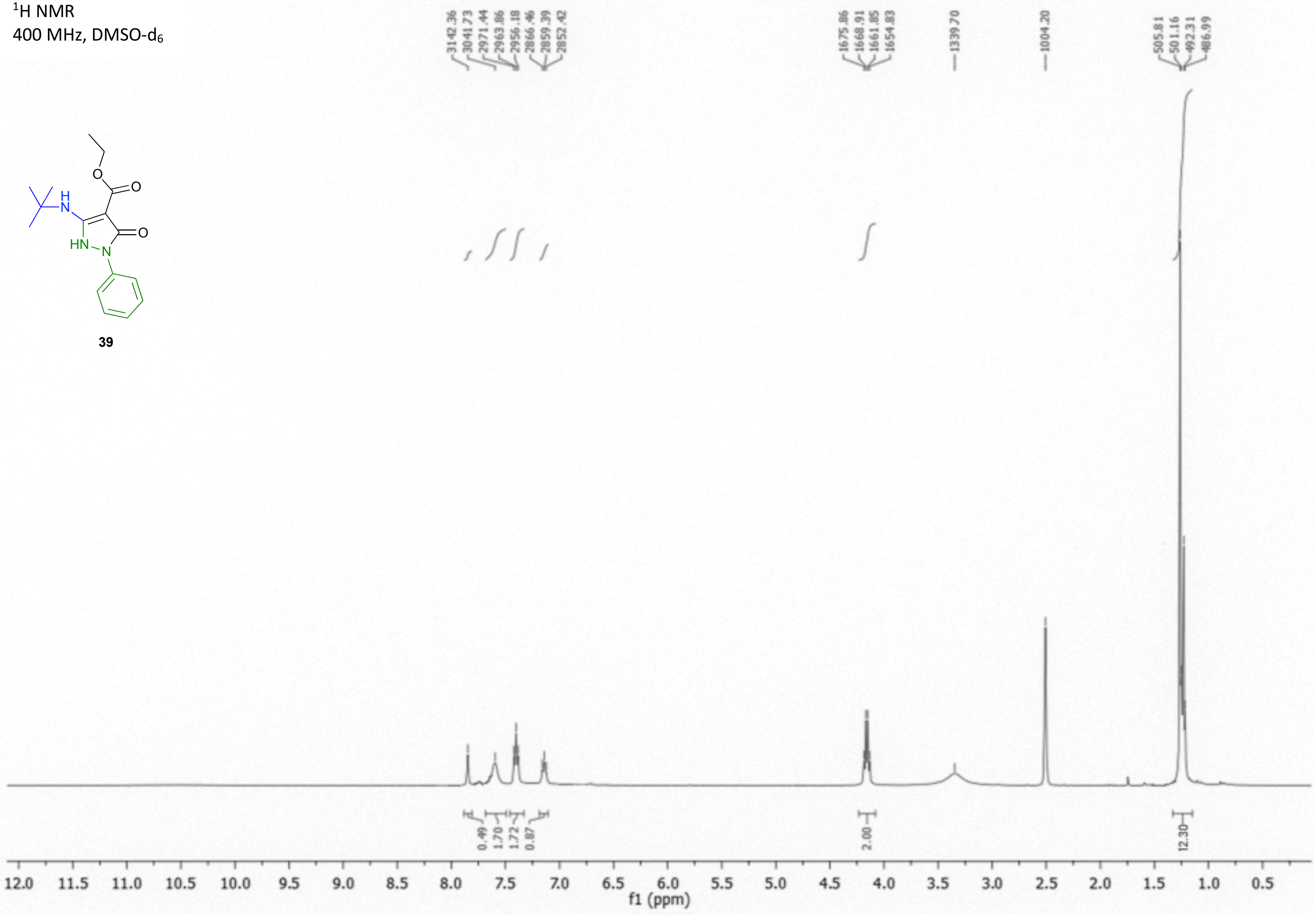

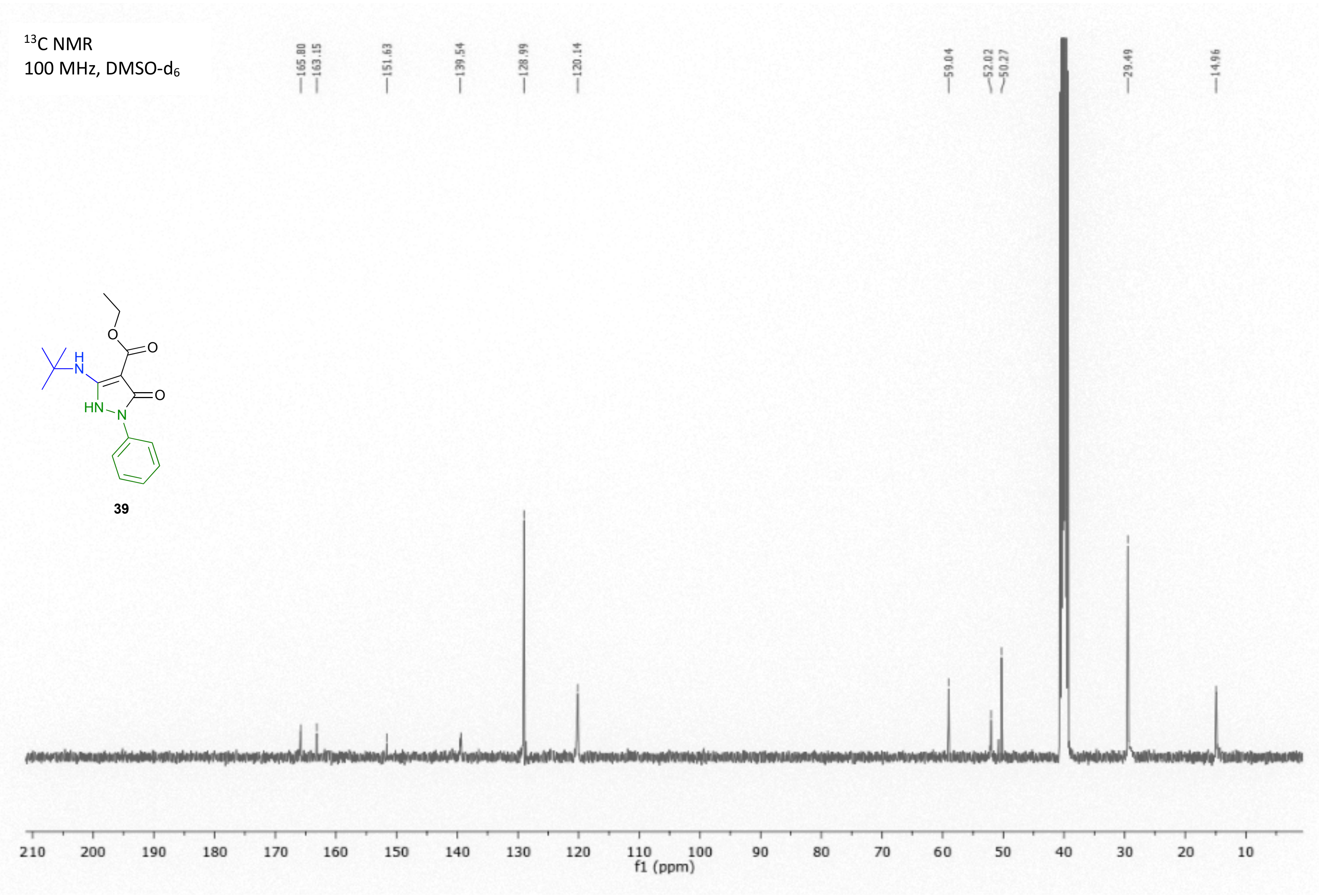

^{13}C NMR
100 MHz, DMSO-d$_6$
165.80
163.15
151.63
139.54
128.99
120.14
59.04
52.02
50.27
29.49
14.96
39
210 200 190 180 170 160 150 140 130 120 110 100 90 80 70 60 50 40 30 20 10
f1 (ppm)

^1H NMR
400 MHz, $CDCl_3$

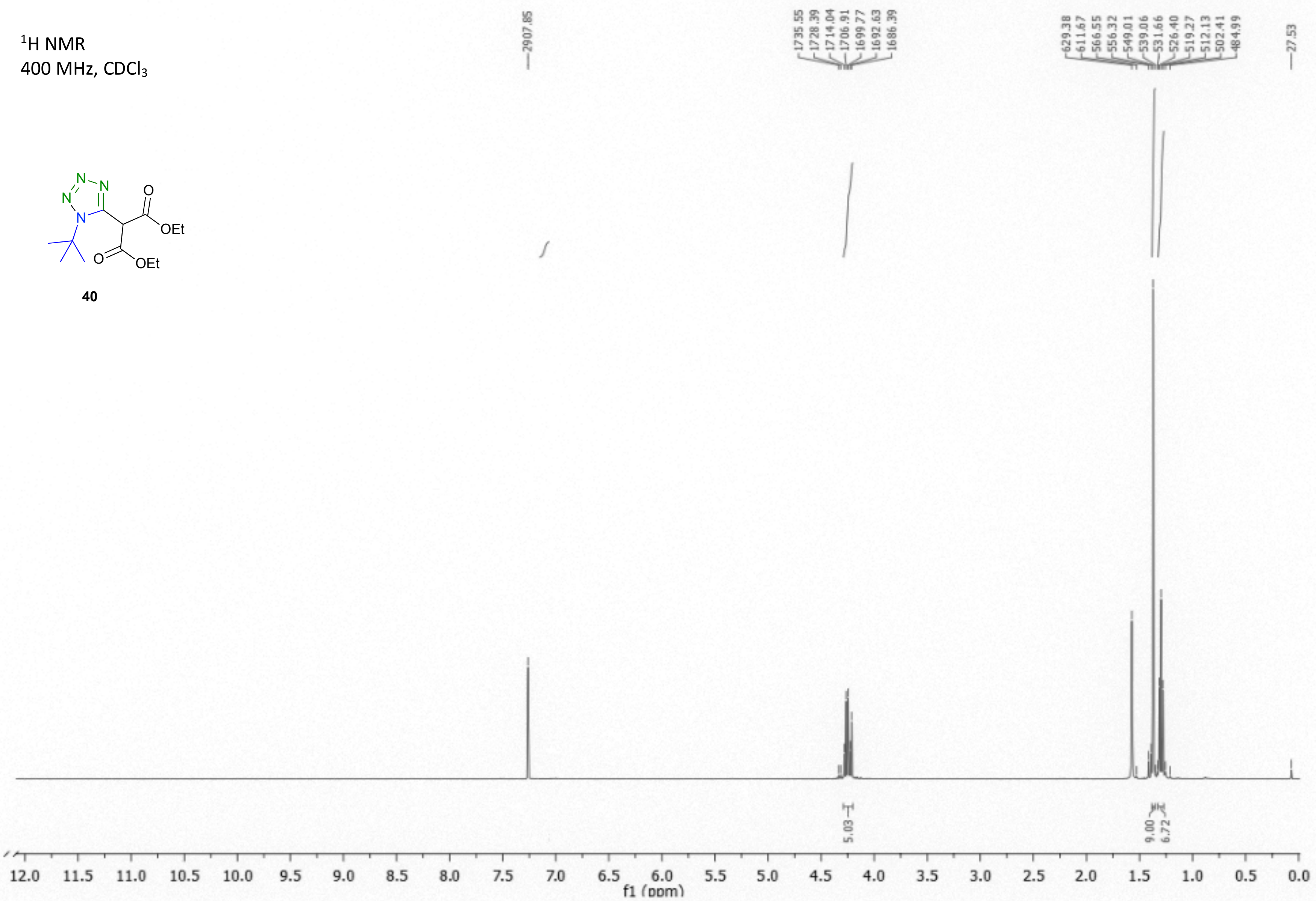

^{13}C NMR
100 MHz, $CDCl_3$

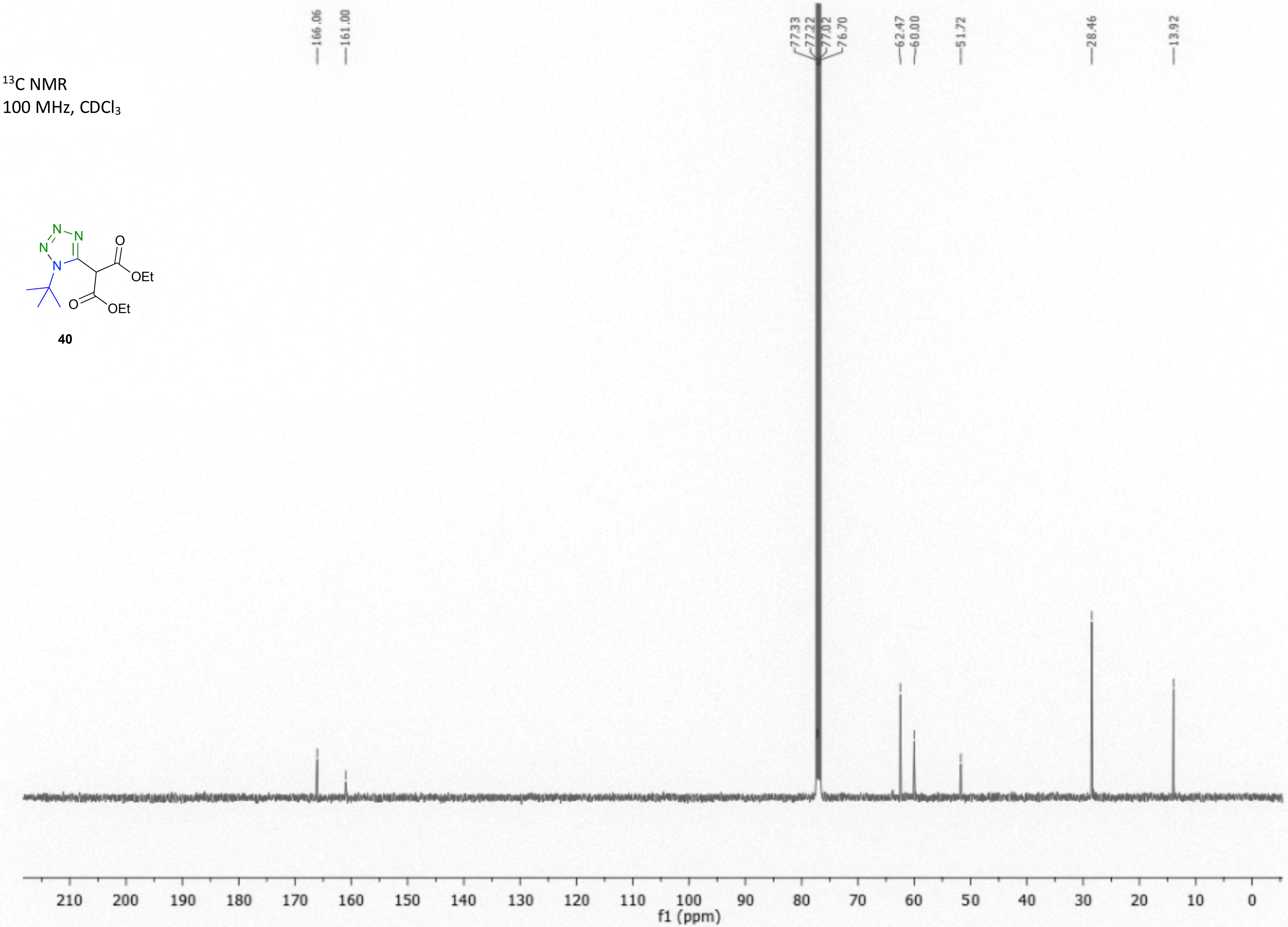

Supplement: Supplementary file 1 — jo0c01946_si_001.pdf [file jo0c01946_si_001.pdf]
